# Shared and specific associations of amygdala nuclei volumes with PTSD symptom domains and childhood trauma: An ENIGMA-PGC PTSD mega-analysis

**DOI:** 10.1038/s41380-026-03718-w

**Published:** 2026-07-02

**Authors:** Mary S. Mufford, Dennis van der Meer, C. Lexi Baird, Delin Sun, Kevin S. LaBar, Raj Ramesar, Ahmed Hussain, Chadi G. Abdallah, Elpiniki Andrew, Christopher L. Averill, Justin T. Baker, Lee A. Baugh, Jennifer U. Blackford, Jessica Bomyea, Richard A. Bryant, Christopher R. K. Ching, Kyle Choi, Andrew S. Cotton, Judith K. Daniels, Nicholas D. Davenport, Richard J. Davidson, Michael D. De Bellis, Emily L. Dennis, Maria Densmore, Terri A. deRoon-Cassini, Seth G. Disner, Stefan du Plessis, Amit Etkin, Negar Fani, Kelene A. Fercho, Jacklynn Fitzgerald, Elbert Geuze, Evan M. Gordon, Daniel W. Grupe, Ilan Harpaz-Rotem, Courtney C. Russel, Julia I. Herzog, David Hofmann, Anna R. Hudson, Jonathan Ipser, Neda Jahanshad, Tanja Jovanovic, Milissa L. Kaufman, Saskia B. J. Koch, Inga K. Koerte, Mayuresh S. Korgaonkar, John H. Krystal, Ruth Lanius, Christine Larson, Lauren A. M. Lebois, Ifat Levy, Gen Li, Israel Liberzon, Mark W. Logue, Vincent A. Magnotta, Antje Manthey, Adi Maron-Katz, Geoffrey May, Katie A. McLaughlin, Sven C. Mueller, Laura Nawijn, Steven M. Nelson, Yuval Neria, Richard W. J. Neufeld, Jack B. Nitschke, Erin N. O’Leary, Bunmi O. Olatunji, Miranda Olff, Elizabeth A. Olson, Matthew Peverill, K. Luan Phan, Evelyn Pineda, Kerry J. Ressler, Isabelle M. Rosso, Lauren E. Salminen, Kelly Meltzer, Christian Schmahl, Soraya Seedat, Caitlin A. Sharp, Martha E. Shenton, Anika Sierk, Raluca M. Simons, Jeffrey S. Simons, Scott R. Sponheim, Murray B. Stein, Jennifer S. Stevens, Thomas Straube, Benjamin Suarez-Jimenez, Jean Théberge, Sophia I. Thomopolous, Nic J. A. van der Wee, Steven J. A. van der Werff, Theo G. M. van Erp, Sanne J. H. van Rooij, Mirjam van Zuiden, Dick J. Veltman, Robert R. J. M. Vermeiren, Henrik Walter, Xin Wang, Li Wang, Hong Xie, Xi Zhu, Ye Zhu, Paul M. Thompson, Ole A. Andreassen, Dan J. Stein, Rajendra A. Morey, Shareefa Dalvie

**Affiliations:** 1 Division of Human Genetics, School of Pathology, Faculty of Health Sciences, National Health Laboratory Service, University of the Witwatersrand, Johannesburg, South Africa; 2 South African Medical Research Council Unit on Risk & Resilience in Mental Disorders, Department of Psychiatry and Neuroscience Institute, University of Cape Town, Cape Town, South Africa; 3 Centre for Precision Psychiatry, Division of Mental Health and Addiction, University of Oslo and Oslo University Hospital, Oslo, Norway; 4 School of Mental Health and Neuroscience, Faculty of Health, Medicine and Life Sciences, Maastricht University, Maastricht, Netherlands; 5 Brain Imaging Analysis Center, Duke University, Durham, NC USA; 6 Department of Veteran Affairs Mid-Atlantic Mental Illness Research, Education and Clinical Center, Durham, NC USA; 7 Department of Psychology, Pennsylvania State University, University Park, PA USA; 8 Department of Psychiatry and Behavioral Sciences, School of Medicine, Duke University, Durham, NC USA; 9 Center for Cognitive Neuroscience, Duke University, Durham, NC USA; 10 South African Medical Research Council Genomic and Precision Medicine Research Unit, Division of Human Genetics, Department of Pathology, Institute of Infectious Disease and Molecular Medicine, University of Cape Town, Cape Town, South Africa; 11 Department of Psychiatry, Baylor college of medicine, Houston, TX USA; 12 Department of Psychiatry, Yale University School of Medicine, New Haven, CT USA; 13 Department of Psychology, University of Sydney, Westmead, NSW Australia; 14 Core for Advanced MRI (CAMRI), Baylor College of Medicine, Houston, TX USA; 15 Institute for Technology in Psychiatry, McLean Hospital, Belmont, MA USA; 16 Massachusetts General Brigham Department of Psychiatry, Boston, MA USA; 17 Harvard Medical School, Boston, MA USA; 18 Division of Basic Biomedical Sciences, Sanford School of Medicine, University of South Dakota, Vermillion, SD USA; 19 Center for Brain and Behavior Research, University of South Dakota, Vermillion, SD USA; 20 Sioux Falls VA Health Care System, Sioux Falls, SD USA; 21 Munroe-Meyer Institute, University of Nebraska Medical Center, Omaha, NE USA; 22 Department of Psychiatry, University of California San Diego, San Diego, CA USA; 23 VA San Diego Center of Excellence for Stress and Mental Health, San Diego, CA USA; 24 School of Psychology, University of New South Wales, Sydney, NSW Australia; 25 Black Dog Institute, Sydney, Australia; 26 Imaging Genetics Center, Mark and Mary Stevens Neuroimaging and Informatics Institute, Keck School of Medicine of USC, University of Southern California, Marina del Rey, CA USA; 27 Health Services Research Center, University of California San Diego, San Diego, CA USA; 28 Department of Neurology, Johns Hopkins School of Medicine, Baltimore, MD USA; 29 Department of Clinical Psychology, University of Groningen, Groningen, Netherlands; 30 Traumacentrum Beilen, GGZ Drenthe, Beilen, Netherlands; 31 Research, Minneapolis VA Health Care System, Minneapolis, MN USA; 32 Department of Psychiatry and Behavioral Sciences, University of Minnesota Medical School, Minneapolis, MN USA; 33 Center for Healthy Minds, University of Wisconsin-Madison, Madison, WI USA; 34 Department of Psychiatry, University of Wisconsin-Madison, Madison, WI USA; 35 Department of Psychology, University of Wisconsin-Madison, Madison, WI USA; 36 Department of Neurology, University of Utah School of Medicine, Salt Lake City, UT USA; 37 George E. Wahlen Veterans Affairs Medical Center, Salt Lake City, UT USA; 38 Department of Psychiatry, Western University, London, ON Canada; 39 Division of Trauma and Acute Care Surgery, Department of Surgery, Medical College of Wisconsin, Milwaukee, WI USA; 40 Comprehensive Injury Center, Medical College of Wisconsin, Milwaukee, WI USA; 41 Mental Health Integrated Care Community, Minneapolis VA Health Care System, Minneapolis, MN USA; 42 Department of Psychiatry, Faculty of Medicine and Health Sciences, Stellenbosch University, Cape Town, South Africa; 43 South African Medical Research Council on the Genomics of Brain Disorders, Department of Psychiatry, Faculty of Medicine and Health Sciences, Stellenbosch University, Cape Town, South Africa; 44 Alto Neuroscience, Inc, Mountain View, CA USA; 45 Department of Psychiatry and Behavioral Sciences, Stanford University, Stanford, CA USA; 46 Department of Psychiatry and Behavioral Sciences, Emory University School of Medicine, Atlanta, GA USA; 47 Federal Aviation Administration, Washington, D.C., USA; 48 Department of Psychology, Marquette University, Milwaukee, WI USA; 49 Brain Research & Innovation Centre, Ministry of Defence, Utrecht, Netherlands; 50 Department of Psychiatry, Utrecht University Medical Center, Utrecht, Netherlands; 51 Mallinckrodt Institute of Radiology, Washington University School of Medicine, St. Louis, MO USA; 52 Division of Clinical Neuroscience, National Center for PTSD, West Haven, CT USA; 53 Department of Psychology, Yale University, New Haven, CT USA; 54 Department of Psychosomatic Medicine and Psychotherapy, Central Institute of Mental Health, Medical Faculty Mannheim, Heidelberg University, Heidelberg, Germany; 55 Institute of Medical Psychology and Systems Neuroscience, University of Münster, Münster, Germany; 56 Department of Experimental Clinical and Health Psychology, Ghent University, Ghent, Belgium; 57 Department of Psychiatry and Behavioral Neuroscience, Wayne State University School of Medicine, Detroit, MI USA; 58 Department of Psychiatry, Harvard Medical School, Boston, MA USA; 59 Division of Women’s Mental Health, McLean Hospital, Harvard University, Belmont, MA USA; 60 Department of Psychiatry, University of Amsterdam, Amsterdam, Netherlands; 61 Donders Institute for Brain, Cognition and Behavior, Centre for Cognitive Neuroimaging, Radboud University Nijmegen, Nijmegen, Netherlands; 62 Psychiatry Neuroimaging Laboratory, Brigham & Women’s Hospital, Boston, MA USA; 63 Department of Child and Adolescent Psychiatry, Psychosomatics, and Psychotherapy, Ludwig-Maximilians-Universität, Munich, Germany; 64 Brain Dynamics Centre, Westmead Institute of Medical Research, Sydney, NSW Australia; 65 Discipline of Psychiatry, Sydney Medical School, Faculty of Medicine and Health, The University of Sydney, Sydney, NSW Australia; 66 Department of Neuroscience, Western University, London, ON Canada; 67 Department of Psychology, University of Wisconsin-Milwaukee, Milwaukee, WI USA; 68 Division of Depression and Anxiety Disorders, McLean Hospital, Harvard University, Belmont, MA USA; 69 Department of Comparative Medicine, Yale School of Medicine, New Haven, CT USA; 70 Department of Neuroscience, Yale School of Medicine, New Haven, CT USA; 71 Laboratory for Traumatic Stress Studies, State Key Laboratory of Cognitive Science and Mental Health, Institute of Psychology, Chinese Academy of Sciences, Beijing, China; 72 Department of Psychology, University of Chinese Academy of Sciences, Beijing, China; 73 Department of Psychiatry and Behavioral Science. Texas A&M University College of Medicine, College Station, TX USA; 74 National Center for PTSD: Behavioral Sciences Division, VA Boston Healthcare System, Boston, MA USA; 75 Department of Psychiatry, Boston University Chobanian & Avedisian School of Medicine, Boston, MA USA; 76 Department of Medicine: Biomedical Genetics, Boston University Chobanian & Avedisian School of Medicine, Boston, MA USA; 77 Department of Radiology, Psychiatry, and Biomedical Engineering, University of Iowa, Iowa City, IA USA; 78 Health and Medical University Potsdam, Potsdam, Germany; 79 Veterans Integrated Service Network-17 Center of Excellence for Research on Returning War Veterans, Waco, TX USA; 80 Department of Psychology and Neuroscience, Baylor University, Waco, TX USA; 81 Center for Vital Longevity, School of Behavioral and Brain Sciences, University of Texas at Dallas, Dallas, TX USA; 82 Ballmer Institute for Children’s Behavioral Health, University of Oregon, Portland, OR USA; 83 Department of Psychology, Harvard University, Cambridge, MA USA; 84 Department of Clinical Psychology, Leiden University, Leiden, Netherlands; 85 Department of Pediatrics and Masonic Institute for the Developing Brain, University of Minnesota, Minneapolis, MN USA; 86 Department of Psychiatry, Columbia University Medical Center, New York, NY USA; 87 New York State Psychiatric Institute, New York, NY USA; 88 Department of Psychiatry, University of Toledo, Toledo, OH USA; 89 Department of Psychology, Vanderbilt University, Nashville, TN USA; 90 Department of Psychiatry, Amsterdam UMC, University of Amsterdam, Amsterdam, Netherlands; 91 ARQ National Psychotrauma Centre, Diemen, Netherlands; 92 Center for Depression, Anxiety, and Stress Research, McLean Hospital, Harvard University, Belmont, MA USA; 93 Department of Psychiatry & Behavioral Health, The Ohio State University, Columbus, OH USA; 94 Department of Neuroscience, University of Rochester Medical Center, Rochester, New York, USA; 95 Department of Psychiatry, McLean Hospital, Harvard Medical School, Belmont, MA USA; 96 Department of Radiology, University of Washington, Seattle, WA USA; 97 Massachusetts General Hospital, Boston, MA USA; 98 Department of Psychiatry and Psychotherapy, University Medical Centre Charité, Berlin, Germany; 99 Department of Psychology, University of South Dakota, Vermillion, SD USA; 100 Minneapolis VA Health Care System, Minneapolis, MN USA; 101 Department of Psychiatry, University of California San Diego, La Jolla, CA USA; 102 School of Public Health, University of California San Diego, La Jolla, CA USA; 103 Department of Psychiatry, Leiden University Medical Center, Leiden, Netherlands; 104 Leiden Institute for Brain and Cognition, Leiden, Netherlands; 105 Department of Psychiatry and Human Behavior, University of California Irvine, Irvine, CA USA; 106 Center for the Neurobiology of Learning and Memory, University of California Irvine, Irvine, CA USA; 107 Department of Clinical Psychology, Utrecht University, Utrecht, Netherlands; 108 Department of Psychiatry & Psychology, Leiden University Medical Center, Leiden, Netherlands; 109 Department of Psychiatry and Clinical Neuroscience, Charité – Universitätsmedizin Berlin, corporate member of Freie Universität Berlin, Humboldt-Universität zu Berlin, and Berlin Institute of Health, Berlin, Germany; 110 German Center for Mental Health, Partner Site Berlin-Potsdam, Berlin, Germany; 111 Department of Neuroscience and Psychiatry, University of Toledo, Toledo, OH USA; 112 Department of Bioengineering, University of Texas at Arlington, Arlington, TX USA; 113 KG Jebsen Centre for Neurodevelopmental Disorders, Oslo University Hospital, Oslo, Norway

**Keywords:** Neuroscience, Diagnostic markers

## Abstract

Posttraumatic stress disorder (PTSD) is a psychiatric condition that may develop after trauma exposure. PTSD is characterized by considerable clinical heterogeneity. The amygdala’s key role in fear conditioning makes it an important focus for investigating the neurobiology of PTSD. However, associations between amygdala volume and PTSD have been inconsistent. The amygdala consists of functionally distinct nuclei. Specific associations between amygdala nuclei volumes and PTSD may account for previous discrepancies between PTSD and whole amygdala volume. This study investigates the associations between amygdala nuclei volumes, PTSD diagnosis, severity, symptom cluster scores, age of onset and childhood trauma. Individuals with a PTSD diagnosis (*n* = 771) and controls (*n* = 1 081, 72% trauma-exposed) were sourced from the Enhancing Neuro-Imaging Genetics through Meta-Analysis and Psychiatric Genomics Consortium (mean age = 32.4 years, (SD = 13 years), 60% male). Nine amygdala nuclei volumes were compared to PTSD diagnosis, age of onset, overall severity, symptom cluster scores (re-experiencing, arousal, and avoidance/emotional numbing), and childhood trauma subscales. Analyses were performed using ordinary least-squares regression, corrected for age, sex, intracranial volume, and whole amygdala volume. PTSD diagnosis was not significantly associated with amygdala nuclei volumes. PTSD severity scores were associated with smaller right lateral nucleus volume (β = −0.26, p_BON_ = 0.01). Smaller right lateral nucleus volume was also associated with re-experiencing (β = −1.01, p_BON_ = 0.04) and arousal (β = −0.9, p_BON_ = 0.04), smaller left paralaminar nucleus volume was associated with re-experiencing (β = −0.1, p_BON_ = 0.04), smaller left corticoamygdaloid transition area volume was associated with avoidance (β = −0.31, p_BON_ = 0.02). Larger left and right central nucleus volumes were significantly associated with childhood physical abuse (β = 0.24, p_BON_ = 9 × 10^−3^) and neglect (β = 0.29, p_BON_ = 0.04), respectively. Differences in select amygdala nuclei volumes among adults are associated with PTSD severity, symptom cluster scores, and childhood physical abuse and neglect. These findings demonstrate nuclei-specific patterns consistent with their functional roles in fear learning and expression.

## Introduction

Posttraumatic stress disorder (PTSD) is a complex psychiatric disorder with onset after exposure to a traumatic event, resulting in continued distress and dysfunction [1]. The estimated lifetime prevalence of PTSD among individuals exposed to trauma is 5.6% [2]. The average age of onset is 23 years among adults [3]; however, PTSD can develop at any age [4]. In DSM-IV, PTSD was presented as three symptom clusters: 1) re-experiencing the traumatic event, for example, via intrusive thoughts or recollection of trauma memories; 2) avoidance of reminders of the traumatic event and emotional numbing; and 3) hyperarousal, which is characterized by hypervigilance and irritability [5]. The DSM-5 added a fourth symptom cluster related to negative alterations in mood and cognition [1].

Amygdala volume has been associated with individual differences in fearfulness [6], socio-emotional competencies [7], and perceived stress [8], making it of particular interest in PTSD research [9]. Animal models of chronic stress reveal dendritic remodeling, synaptic density changes, and region-specific hypertrophy and atrophy in the amygdala [10, 11], which likely underlie volume differences observed in humans. Further, human studies consistently show an inverse relationship between amygdala volume and activity, with smaller amygdala volumes associated with greater activation during stressful or emotional tasks [12, 13]. This suggests that volumetric measurements reflect stable structural alterations, offering complementary insights into long-term changes in neural activity [14]. This stability, along with its associations with relevant behavioral traits and neural activity, indicates that it would be useful to investigate amygdala volume in PTSD.

Associations between amygdala volume and PTSD have, however, been inconsistent, with larger [15], smaller [16], and no significant differences [17] reported. This lack of consistency in findings may result from studies investigating the amygdala as a homogenous structure instead of specific nuclei with distinct neurobiology [18]. The amygdala can be broadly grouped into three functional subfields: 1) the basolateral (BLA) subfield, which is involved in forming associations between environmental cues and threats [19]; 2) the centromedial (CeM) subfield, which orchestrates fear responses (e.g. freezing, fleeing); and 3) the cortical-like complexes involved in olfactory fear conditioning and social communication [20]. The human amygdala can be further subdivided into nine distinct nuclei, with recent evidence suggesting each nucleus has nuanced structural and functional differences beyond those captured in the three broader subfields [21].

This refined level of amygdala granularity, through segmentation of the amygdala into nine nuclei volumes, has been explored in relation to PTSD diagnosis and severity in three small cross-sectional studies [22]. One study, included in our analyses, focused on military veterans and compared individuals with PTSD (*n* = 149) to trauma-exposed control subjects without PTSD (*n* = 206) [16]. This study reported that PTSD severity was associated with smaller volumes in the left and right lateral and paralaminar nuclei and larger left and right central, medial, and cortical nuclei of the amygdala [23]. A subsequent study investigated young survivors of a terrorist attack (PTSD cases *n* = 45, trauma-exposed controls *n* = 54) and identified a significant association between smaller right lateral nucleus volume and PTSD severity [24]. The most recent study investigated adult survivors of a natural disaster (PTSD cases *n* = 69, trauma-exposed controls *n* = 76, unexposed controls *n* = 56) and associated smaller right cortical nucleus volume with higher PTSD severity scores amongst PTSD patients and the inverse association amongst trauma-exposed controls [25].

Trauma, particularly in early life, leaves a lasting neurobiological imprint on the brain, regardless of a subsequent PTSD diagnosis [26]. Childhood trauma may result in the onset of pediatric PTSD and other psychiatric conditions and has also been associated with increased risk, severity, and adverse outcomes amongst adult PTSD patients [27]. Human and animal models suggest that early-life trauma affects the neurodevelopment of communication between the prefrontal cortex, hippocampus, hypothalamus, and amygdala. Specifically, the BLA and CeM are altered in response to chronic stress; for example, there is an attenuation of spine retraction in the medial nucleus [28–33]. These alterations increase the likelihood of adverse behavioral and health outcomes [34]. Furthermore, larger whole amygdala volume and amygdala hyperreactivity after early-life trauma exposure persist for several years [35, 36].

This study aims to confirm previous associations between the nine amygdala nuclei volumes and PTSD diagnosis, severity, and symptom clusters based on relatively small individual study samples, in a well-powered, multi-center sample. Additionally, this study examines associations between amygdala nuclei volumes and the age of PTSD onset, childhood and lifetime trauma exposure. Given the findings from previous association studies and the current understanding of the functional roles of the amygdala nuclei, we speculate that there will be both overlapping and nuanced associations across the PTSD domains explored here that could provide valuable information about PTSD pathophysiology.

## Methods

### Participants and phenotype measures

Twenty-two cohorts contributing to the ENIGMA-PGC PTSD Working Group [37] provided demographic and clinical data and amygdala nuclei volumes for 2 030 participants (PTSD cases *n* = 880, controls *n* = 1 150), including military and civilian participants across diverse ancestries (cohort information is provided in Supplementary Table 1). Following imaging quality control described below, 1 852 individuals were included in the analysis (PTSD cases *n* = 771, controls *n* = 1 081). The age range of the cohort was 6–67 years (mean = 32.4 years, standard deviation (SD) = 13), and 60% of the participants were male. Participants’ clinical and demographic information by PTSD diagnosis are summarized in Table 1. The control group consisted of trauma-exposed (TC, *n* = 780, 72%) and trauma-unexposed (HC, *n* = 271, 25%) participants, as well as participants for whom trauma exposure was undetermined (*n* = 30, 3%). Using standard cut-offs, the lifetime trauma exposure among controls was assessed with the Life Events Checklist [38]. Childhood trauma was specifically defined with the Childhood Trauma Questionnaire (CTQ) [39] for a subset of the cohort (PTSD cases *n* = 187, Controls *n* = 246).

**Table 1:** Clinical and Demographic Features of Study Participants by PTSD Diagnostic Status.

	PTSD		AC		TC		HC		PTSD vs AC		PTSD vs TC		PTSD vs HC		TC vs HC	
	Mean	SE	Mean	SE	Mean	SE	Mean	SE	t or x²	*p*-Value	t or x²	*p*-Value	t or x²	*p*-Value	t or x²	*p*-Value
**n (%)**	771 (42%)^a^		1 081 (58%)^a^		780 (72%)^b^		271 (25%)^b^									
**Age, Years**	34	0.45	31.3	0.4	33.7	0.45	23.9	0.78	−4.57	**<0.001**	0.53	0.59	−11.23	**<0.001**	−10.84	**<0.001**
**Sex, Male, n (% within group)**	454 (59%)		666 (62%)		505 (65%)		142 (52%)		1.23	0.26	14.15	**<0.001**	14.15	**<0.001**	14.15	**<0.001**
**ICV (mm3)**	1516848	6518	1535285	5257	1530578	6272	1548818	9788	2.2	**0.03**	−1.518	0.13	2.72	**<0.001**	1.569	0.12
**PTSD Severity score (CAPS-IV)**	67.30	1.29	11.80	0.96	14.10	1.17	3.36	0.64	35.18	**<0.001**	53.19	**<0.001**	63.95	**<0.001**	10.76	**<0.001**
**CAPS-IV Re-experiencing**	20.00	0.58	2.22	0.38	2.69	0.49	0.82	0.33	25.50	**<0.001**	17.33	**<0.001**	19.19	**<0.001**	1.86	**0.04**
**CAPS-IV Avoidance**	25.10	0.80	3.31	0.57	3.94	0.73	1.39	0.48	21.97	**<0.001**	21.13	**<0.001**	23.68	**<0.001**	2.55	0.06
**CAPS-IV Arousal**	23.50	0.45	6.07	0.66	7.10	0.82	2.75	0.72	21.93	**<0.001**	16.40	**<0.001**	20.75	**<0.001**	4.35	**<0.001**
**CTQ Total**	67.00	1.68	41.80	0.90	42.80	1.09	38.70	1.44	14.05	**<0.001**	24.15	**<0.001**	28.32	**<0.001**	4.18	**0.03**
**CTQ Abuse**	39.10	1.19	21.50	0.56	22.40	0.69	18.70	0.74	14.37	**<0.001**	16.62	**<0.001**	20.35	**<0.001**	3.73	**<0.001**
**CTQ Neglect**	24.20	0.63	17.90	0.51	18.50	0.61	16.20	0.94	7.86	**<0.001**	5.70	**<0.001**	8.02	**<0.001**	2.32	**0.04**
**CTQ Emotional abuse**	15.30	0.47	7.94	0.25	8.23	0.30	7.00	0.40	14.85	**<0.001**	7.08	**<0.001**	8.31	**<0.001**	1.23	**0.02**
**CTQ Physical abuse**	11.00	0.42	7.35	0.23	7.69	0.29	6.45	0.34	8.22	**<0.001**	3.36	**<0.001**	4.60	**<0.001**	1.24	**0.01**
**CTQ Sexual abuse**	12.70	0.57	6.21	0.22	6.50	0.29	5.24	0.11	11.59	**<0.001**	6.18	**<0.001**	7.44	**<0.001**	1.26	**<0.001**
**CTQ Emotional neglect**	13.70	0.43	10.50	0.35	10.90	0.41	9.77	0.65	5.81	**<0.001**	2.89	**<0.001**	3.96	**<0.001**	1.08	0.16
**CTQ Physical neglect**	10.50	0.30	7.35	0.20	7.70	0.24	6.45	0.35	9.10	**<0.001**	2.81	**<0.001**	4.05	**<0.001**	1.24	**<0.001**

*PTSD*, Posttraumatic stress disorder; *AC*, all controls; *TC*, trauma-exposed controls; *HC*, trauma-unexposed controls; *t or z*, t or z test statistic; *CAPS*, Clinician-Administered PTSD Scale; *CTQ*, Child Trauma Questionnaire; *ICV*, intracranial volume.

^a^percentage of total cohort.

^b^percentage of controls.

Across sites, PTSD diagnosis was determined using the Clinician-Administered PTSD Scale (CAPS) for the fourth (49%) [40] or fifth (21%) [41] edition of the Diagnostic and Statistical Manual of Mental Disorders (DSM); the PTSD Checklist for DSM-5 for civilian (PCL-C, 6%) and military (PCL-M, 8%) traumatic experiences [42]; the Structured Clinical Interview for DSM-IV (SCID-IV, 7%) [43]; the Mini-International Neuropsychiatric Interview for DSM-IV (MINI, 5%) [44]; and the Davidson Self-rating PTSD Scale (DTS, 3%) [45]. PTSD age of onset was also captured using either the CAPS-IV or CAPS-5 and ranged from 5 to 60 years (*n* = 150, mean=25, SD = 12.6). PTSD severity and three symptom cluster scores (re-experiencing, avoidance/emotional numbing and arousal) were determined by CAPS-IV and using the shared questions with CAPS-5, which maintained high backwards compatibility with the CAPS-IV [41]. The fourth symptom cluster, negative alterations in mood and cognition, from the CAPS-5 was not included due to the low sample size.

### Quality control of MRI data

At each site, participants underwent high-resolution T1-weighted MRI scans. Each MRI scan was collected with participants’ written informed consent and with approval by local institutional review boards. Using FreeSurfer v6.1, amygdala volumes were segmented into nine nuclei per hemisphere, including the basal, lateral, accessory basal, central, medial, cortical, and paralaminar nuclei, along with the anterior amygdaloid and cortico-amygdaloid transition areas [21]. The segmentation was visually inspected according to ENIGMA protocols (https://enigma.ini.usc.edu/ongoing/enigma-hippocampal-subfields/). Scans that failed segmentation and participants with missing demographic information were excluded (*n* = 141). Further, scans 4 SD below the mean of the Euler number (*n* = 31) [46], intracranial volume (ICV) (*n* = 0), or any amygdala nucleus volume (*n* = 6) were excluded from further analyses (Supplementary Table T2). The distribution of amygdala nuclei volumes is shown in Supplementary Figure S1, and these volumes were normally distributed (Supplementary Table T3 and Supplementary Figure S2).

### Statistical analysis

**Fig. 1:**
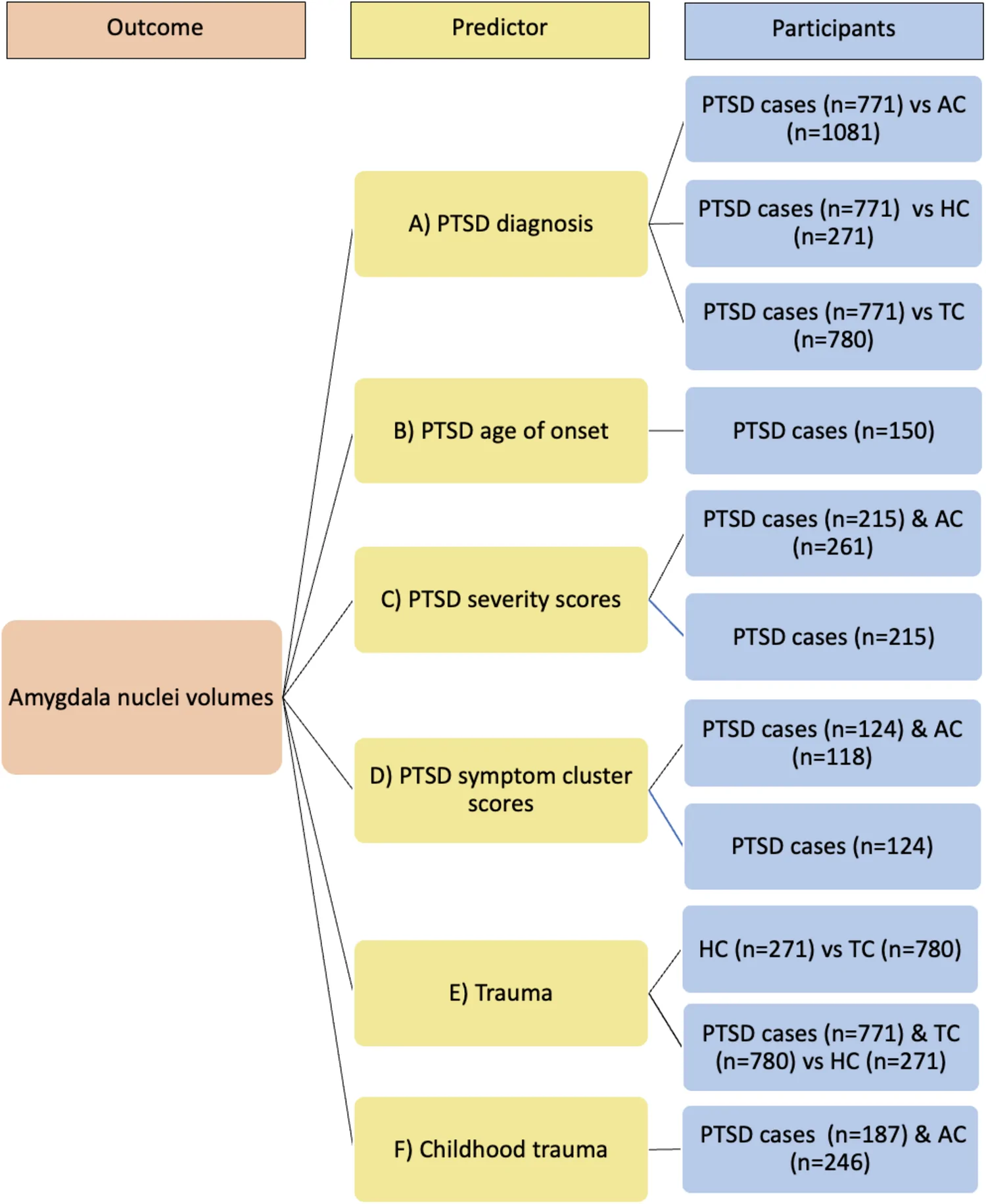
Summary of outcome, predictors and sample sizes used in each regression model. Linear regression models were performed using the outcomes (orange) and predictors (yellow) shown above. The top row of the figure describes the type of data contained in the corresponding color. Each row represents a separate model. PTSD = posttraumatic stress disorder, vs = versus (i.e., groups are compared to one another), &= analyses were conducted across both groups, AC = all controls, HC = trauma-unexposed controls, TC = trauma-exposed controls.

Clinical and demographic characteristics of the sample were analyzed in R (v3.6) [47]. T-tests were used for continuous variables (age in years, ICV, Childhood trauma subscales, PTSD severity and symptom scores) and chi-square tests for categorical variables (self-reported sex). Analyses of amygdala and amygdala nuclei volumes were conducted separately for the left and right hemispheres using ordinary least-squares regression in R (v3.6). Prior to the neuroimaging analyses, ComBat-GAM was used to harmonize the amygdala nuclei volumes across sites using age (years) as a smoothing term and self-reported sex, PTSD diagnosis, ICV, and whole amygdala volume as covariates, where appropriate [48]. The covariates included in each main model were self-reported sex, age in years, age^2^, whole amygdala volume and ICV. Whole amygdala volume was included as a covariate to isolate the unique contribution of each nucleus. Analyses using site and scanner as covariates instead of ComBat-GAM harmonization, and analyses without whole amygdala included as a covariate are available in Supplementary Tables T4 and T5, respectively. Figure 1 provides an overview of all analyses. The predictors for each model included: 1) PTSD diagnosis (Fig. 1, Box A); 2) PTSD age of onset (Fig. 1, Box B); 3) PTSD severity scores (Fig. 1, Box C); 4) PTSD symptom cluster scores (Fig. 1, Box D); 5) lifetime trauma exposure (Fig. 1, Box E); 6) and childhood trauma (Fig. 1, Box F). The model below demonstrates how each regression was conducted using the left hemisphere as an example:

$${{\rm{Left}}}\; {{\rm{amygdala}}}\; {{\rm{nucleus}}}\; {{\rm{volume}}} \sim {{\rm{Predictor}}}{\dagger} +{{\rm{Sex}}}+{{\rm{Age}}}+{{{\rm{Age}}}}^{2}+{{\rm{ICV}}}+{{\rm{Whole}}}\; {{\rm{left}}}\; {{\rm{amygdala}}}\; {{\rm{volume}}}$$

†Predictor = diagnosis, age of onset, symptom severity, symptom cluster scores, trauma, or childhood trauma

For PTSD diagnosis, separate analyses compared PTSD cases with all controls (AC), TC, or HC only. Analyses of PTSD severity, PTSD symptom cluster scores, and childhood trauma were conducted across all PTSD cases and all controls to accommodate the full spectrum of each measure [49]. Sensitivity analyses for PTSD severity and PTSD symptom cluster scores were also conducted in PTSD cases only. CTQ subscales, total scores and combined abuse and neglect were analyzed as linear scores. Each analysis included 20 separate tests (9 nuclei volumes and whole amygdala volume for left and right hemispheres). To ensure both robust and balanced control of Type 1 errors, we applied the Benjamini-Hochberg false-discovery rate (FDR) method [50] and the more stringent Bonferroni correction (BON) [51] for 20 tests to each analysis.

## Results

**Fig. 2:**
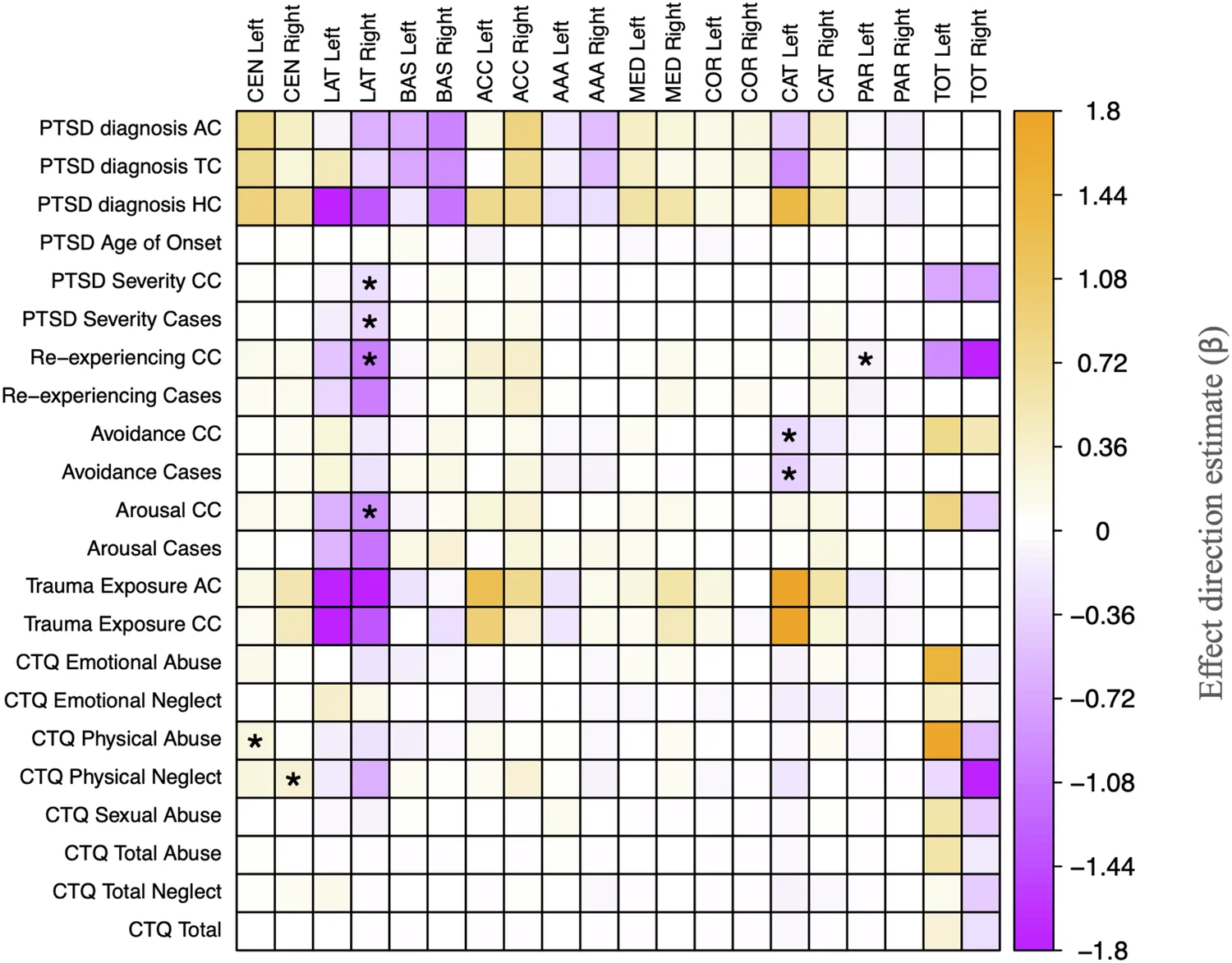
Comparison of amygdala nuclei volumes across PTSD diagnosis and related measures. The x-axis displays the amygdala nuclei for both the left and right hemispheres. The y-axis displays posttraumatic stress disorder (PTSD) diagnosis compared to all controls (AC), trauma-exposed controls (TC) and trauma-unexposed controls (HC); PTSD age of onset; PTSD severity scores as measured with the CAPS-IV compared across all cases and all controls (CC) or cases only; PTSD DSM-IV symptom cluster scores (re-experiencing, avoidance and arousal) compared across all cases and all controls (CC) or cases only, trauma exposure comparing trauma-exposed and unexposed controls (AC) or by comparing PTSD cases and trauma-exposed controls to trauma-unexposed controls, and Childhood Trauma Questionnaire (CTQ) derived subscales (emotional abuse and neglect; physical abuse and neglect; sexual abuse; combined abuse, combined neglect and total scores). The colour bars indicate the effect direction estimate (β), ranging from larger volumes in yellow to smaller volumes in purple The beta estimates have been capped at 1.8 and −1.8 for display purposes. Significant findings at p < 0.05 after Bonferroni correction are indicated by an asterisk (*). CEN= central nucleus, LAT= lateral nucleus, BAS= basal nucleus, ACC= accessory basal nucleus, AAA= anterior amygdaloid area, MED= medial nucleus, COR= cortical nucleus, CAT= corticoamygdaloid transition area, PAR= paralaminar nucleus.

A summary of effect sizes and significant findings for the main analyses are shown in Fig. 2, and complete statistics are available in Supplementary Table T6. The results of the main analyses remained consistent across both Bonferroni and FDR corrections for multiple testing, with no additional significant findings identified using the less stringent FDR approach. Analyses without ComBat-GAM harmonization, and instead using site and scanner as covariates, are summarized in Supplementary Figure S3 and Supplementary Table T4. Analyses without whole amygdala volume as a covariate are summarized in Supplementary Figure S4 and Supplementary Table T5.

### Clinical and demographic characterization of the sample

As expected, PTSD severity scores, symptom cluster scores, and childhood trauma scores were significantly higher among PTSD cases (t = 5.81–35.18, *p* < 0.001) (Table 1). PTSD cases were significantly older (t = −4.57, *p* < 0.001) and had significantly larger ICV than controls (t = 2.2, *p* = 0.03). TC also had significantly higher PTSD severity scores, symptom cluster scores (except for avoidance/numbing), and childhood trauma subscale scores (except for emotional neglect) than HC (t = 1.23–10.76, *p* < 0.04). The TC group also had significantly more male participants (z = 0.57, *p* = 1.22 × 10^−3^) and were older than HC (t = 4.38, *p* = 1,76 × 10^−6^).

### PTSD diagnosis and age of onset

Despite relatively large β estimates, none of the amygdala nuclei volumes were significantly associated with PTSD diagnosis after correction for multiple testing. At an uncorrected threshold of *p* < 0.05, PTSD diagnosis was associated with larger left central nucleus volume (β = 0.81, SE = 0.32, t = 2.51, *p* = 1.22 × 10^−2^), smaller right Anterior amygdaloid area (β = −0.51, SE = 0.25, t = −2, *p* = 4.53 × 10^−2^) and smaller left (β = −16.79, SE = 7.11, t = −2.36, *p* = 1.83 × 10^−2^) and right whole amygdala volume (β = −25.19, SE = 7.32, t = −3.44, *p* = 5.9 × 10^−3^) when comparing PTSD cases and all controls. When comparing PTSD cases and TC, PTSD diagnosis was associated with larger left (β = 0.83, SE = 0.36, t = 2.3, *p* = 0.02) and right central nucleus volume (β = 0.83, SE = 0.36, t = 2.3, *p* = 0.02) and smaller left (β = −16.79, SE = 7.11, t = −2.36, *p* = 1.83 × 10^−2^) and right whole amygdala volume (β = −25.19, SE = 7.32, t = −3.44, *p* = 5.9 × 10^−3^). When comparing PTSD cases and HC, PTSD diagnosis was associated with smaller right whole amygdala volume (β = −35.24, SE = 11.27, t = −3.13, *p* = 2.32 × 10^−2^). No significant differences in amygdala nuclei volumes were associated with PTSD age of onset.

### PTSD severity and symptom cluster scores

Higher PTSD severity scores were significantly associated with smaller right lateral nucleus volume across diagnostic status (β = −0.26, SE = 0.08, t = −3.44, p_BON_ = 0.01) and this effect was stronger amongst the cases only (β = −0.35, SE = 0.1, t = −3.64, p_BON_ = 7 × 10^−3^). Higher re-experiencing symptoms were also significantly associated with smaller right lateral nucleus volume (β = −1.01, SE = 0.33, t = −3.09, p_BON_ = 0.04) and left paralaminar nucleus volume (β = −0.1, SE = 0.03, t = −3.13, p_BON_ = 0.04) across diagnostic status, but was not observed amongst the cases only. Higher avoidance/emotional numbing symptom cluster scores were significantly associated with smaller left cortico-amygdaloid transition area volume (β = −0.31, SE = 0.09, t = −3.36, p_BON_ = 0.02) across diagnostic status and this effect was stronger amongst the cases only (β = −0.38, SE = 0.11, t = −3.56, p_BON_ = 0.01). Higher arousal symptom cluster scores were significantly associated with smaller right lateral nucleus volume (β = −0.9, SE = 0.29, t = −3.14, p_BON_ = 0.04), but was not observed amongst the cases only.

### Trauma exposure

Despite relatively large β estimates, associations with lifetime trauma exposure did not survive correction for multiple testing amongst controls or across diagnostic status. At an uncorrected threshold of *p* < 0.05, lifetime trauma exposure amongst controls (β = 2.25, SE = 0.86, t = 2.62, *p* = 9 × 10^−3^) and across PTSD diagnostic status (β = 2.23, SE = 0.83, t = 2.84, *p* = 4.5 × 10^−3^) was associated with larger left cortico-amygdaloid transition area volume.

### Childhood trauma

More severe physical abuse was associated with a larger left central nucleus volume (β = 0.24, SE = 0.07, t = 3.54, p_BON_ = 9.06 × 10^−3^) and more severe physical neglect was associated with a larger right central nucleus volume (β = 0.29, SE = 0.1, t = 3.04, p_BON_ = 4.96 × 10^−3^). No significant differences in amygdala nuclei volumes were detected for the remaining CTQ subscales (sexual abuse and neglect, emotional abuse and neglect, total score or combined abuse and neglect scores).

## Discussion

To the best of our knowledge, this is the most extensive investigation of the association of amygdala nuclei volumes with PTSD diagnosis, severity, and symptom dimensions and one of the first to examine associations with PTSD age of onset and exposure to childhood trauma. While there was no association between overall amygdala volume or amygdala nuclei volumes and PTSD diagnosis, PTSD symptom severity was associated with smaller right lateral nucleus volume. The lateral nucleus was also negatively associated with re-experiencing and hyperarousal symptoms. Further, paralaminar nucleus volume was also negatively associated with re-experiencing, and cortico-amygdaloid transition area volume was negatively associated with avoidance/emotional numbing symptoms. These results suggest unique and overlapping correlations between PTSD symptom dimensions and amygdala nuclei. Exposure to childhood physical abuse and neglect was positively associated with central nucleus volume in our sample of predominantly trauma-exposed individuals.

Significantly lower right lateral nucleus volume was associated with increased PTSD severity scores in this study. These findings corroborate those from two prior studies [23, 24], that were previously discussed. Discrepancies concerning other amygdala nuclei may be due to crucial differences in study design, such as the type of trauma, time since exposure, the age of the participants, the tools used to measure symptom scores and trauma exposure, and the difficulty in isolating trauma- and PTSD-specific effects. The relationship between PTSD severity and smaller lateral nucleus volume is particularly notable due to its role as a sensory hub for highly processed information from sensory association cortices involved in fear conditioning [52].

Specific nuclei volumes were associated with PTSD symptom cluster scores. Our findings suggest that 1) re-experiencing and arousal symptoms have an overlapping association with the amygdala through an association with the lateral nucleus; 2) avoidance/emotional numbing symptoms appear to have a comparatively distinct relation to amygdala pathways relative to the other symptom clusters, as it was the only symptom cluster associated with cortico-amygdaloid transition area volume, and 3) re-experiencing symptoms are associated with two distinct nuclei, lateral and paralaminar nucleus volumes, as compared to the single associations observed for the other symptom clusters. Re-experiencing involves interconnections between several prefrontal and sensory association cortices, and subcortical regions, which may require the involvement of more amygdala nuclei [53].

Animal models have implicated lateral nucleus activity in retrieving fear memories and in the recovery from fear [54]. Altered synaptic potentiation in the lateral nucleus in rats has also been shown to be involved in the disruption of memory reconsolidation, which stabilizes reactivated fear memories [55]. Animal models have identified several long-lasting structural changes in BLA neurons following chronic stress exposure; these changes are associated with subsequent exaggerated anxiety-like behaviors, including heightened anticipatory defensive responses [10, 11]. Further, hyperarousal has been associated with lower lateral nucleus volume in humans before [23]. Lower lateral nucleus volume has also been associated with panic disorder [56] and major depression [57], conditions which are highly comorbid with PTSD and have overlapping symptomatology [58]. These previous associations add support for the possible role of the lateral nucleus in both the re-experiencing and arousal symptom clusters of PTSD, identified here.

Less is known about the paralaminar nucleus, but the high prevalence of immature cells in this nucleus suggests it may be involved in neuroplasticity [59]. The paralaminar nucleus also has high concentrations of corticotropin-releasing hormone (CRH) receptors, which may be influenced by stress, particularly trauma [59]. Evidence suggests that the paralaminar nucleus is involved in contextual fear learning [59], which is the association of neutral stimuli with traumatic experiences [9]. The paralaminar nucleus’ possible susceptibility/ adaptation to stress and association with contextual fear learning may explain its role in mediating re-experiencing symptoms.

The relatively undifferentiated cortico-amygdaloid transition area is located between the amygdala and hippocampus and provides the primary intrinsic input to the lateral nucleus. The hippocampal projections to the cortico-amygdaloid transition area indicate that contextual emotional memories may modulate the lateral nucleus via the cortico-amygdaloid transition area [60]. These contextual memories may drive avoidance behaviors.

PTSD severity, symptom cluster scores and childhood trauma were significantly associated with different amygdala nuclei volumes, whereas associations with PTSD diagnosis did not survive multiple corrections. Recent genome-wide association studies of linear PTSD severity have also revealed significantly higher power for discovery and heritability as compared to binary case-control analyses [61, 62]. PTSD is a highly heterogeneous disorder, diagnosis with DSM-5 allows for over 600,000 different symptom combinations [63]. Due to stringent criteria for diagnosis that require a minimum number of symptoms from each cluster, individuals may have high symptom severity scores without a PTSD diagnosis or have a relatively low severity score but are nevertheless diagnosed with PTSD [63]. Taken together, our findings are in line with current trends in PTSD research.

We found that physical childhood trauma was associated with a larger central nucleus volume. The central nucleus is the hub for orchestrating behavioral and autonomic fear expression in response to internal systemic stressors, such as pain [20, 64]. Pain typically initiates activation of the right amygdala and the memory is then sustained in the left amygdala [65]. It is, therefore, possible that childhood physical abuse is localized as hypertrophy in the amygdala in adulthood. Regarding physical neglect, early institutional deprivation has been associated with larger right amygdala volume [66]. Our findings, therefore, suggest that the evolutionarily primitive central nucleus is a likely candidate for trauma-related changes, particularly in response to physical trauma types [67].

The associations between PTSD severity, symptom cluster scores and childhood trauma had differing effect directions, some being associated with larger nuclei volumes, whereas others were associated with smaller nuclei volumes. Whole amygdala volumes were not associated in any of these analyses. Therefore, by only examining the whole amygdala volume, these more refined patterns of change may be overlooked.

Although we have identified associations between amygdala nuclei volumes with PTSD and trauma phenotypes worthy of follow-up, this study has several limitations. All measures were collected retrospectively and are thus subject to reporting bias, often secondary to incomplete or inaccurate memory recall [68]. It is not possible to parse out childhood trauma exposure from trauma that was experienced in adulthood using the Life Events Checklist, so there may be an overlap between these measures. There is also variability in the tools used to determine PTSD diagnosis, as some of the questionnaires are clinician-administered and others are self-report scales. This study is also cross-sectional, and the findings should not be interpreted as developmental sequelae; i.e., we cannot determine whether the volumetric changes preceded or are a consequence of any of the PTSD-related measures investigated here. Further, the cross-sectional nature makes it challenging to disentangle trauma- and PTSD-specific effects, especially as no significant findings were identified between TC and HC. Future, well-powered longitudinal studies will be vital for refining the link between PTSD-related measures and the amygdala. The reliance of FreeSurfer segmentation on atlas priors, particularly when the algorithm has insufficient information from image contrast, may have diluted the sample heterogeneity, thereby obscuring clinical associations. Quantitative assessments of FreeSurfer’s amygdala nuclei segmentation also show that while numerical reliability is generally acceptable, only 44% of amygdala nuclei meet spatial reliability thresholds, with particularly low reliability in regions smaller than 200mm^3^, such as the paralaminar and central nuclei [69]. These findings underscore the need for caution when interpreting volumetric results from less reliable subregions. Moreover, focusing solely on amygdala volume does not capture the full complexity of brain structure and function in PTSD. Volumetric changes may reflect neural alterations, but do not provide detailed insights into the underlying mechanisms. Future, well-powered longitudinal studies incorporating multi-modal neuroimaging techniques may contribute to understanding the role of the amygdala and the specific neurobiological mechanisms involved in PTSD.

Keeping these limitations in perspective, our study has numerous strengths. Chiefly, our study contains a large cohort of over 1500 participants that represent diverse geography, demography (sex, age, ancestry), trauma type (childhood trauma, military, sexual violence, natural disasters, etc.), and clinical comorbidity. Such sample heterogeneity enhances the generalizability and reproducibility. The moderate effect sizes reported herein mean that the reported associations were detected with robust statistical power, given the current sample size. Finally, the harmonization of volume measures sourced from 22 sites with different MRI scanners was addressed robustly with ComBat-GAM [70].

Overall, we demonstrate a subregion-specific pattern of associations across amygdala nuclei volumes and PTSD severity, symptom clusters, and childhood trauma. This study highlights the importance of examining amygdala nuclei volumes to deepen our understanding of the amygdala’s role in PTSD and clarify associations with PTSD-related dimensions. With further replication and validation, alongside an exploration of the cellular mechanisms and functional consequences of volume differences in the identified nuclei, this approach could enhance our understanding of PTSD pathophysiology.

## Supplementary information

Supplementary Figures

Supplementary Tables in PDF

Supplementary Tables in excel
