# Supplementary material for: Shared and specific associations of amygdala nuclei volumes with PTSD symptom domains and childhood trauma: An ENIGMA-PGC PTSD mega-analysis
Source: Mol Psychiatry. 2026 Jul 2;31(11):6488–97. doi: 10.1038/s41380-026-03718-w (PMC13668723; doi:10.1038/s41380-026-03718-w)
Supplement: Supplementary file 1 [file 41380_2026_3718_MOESM1_ESM.pdf]

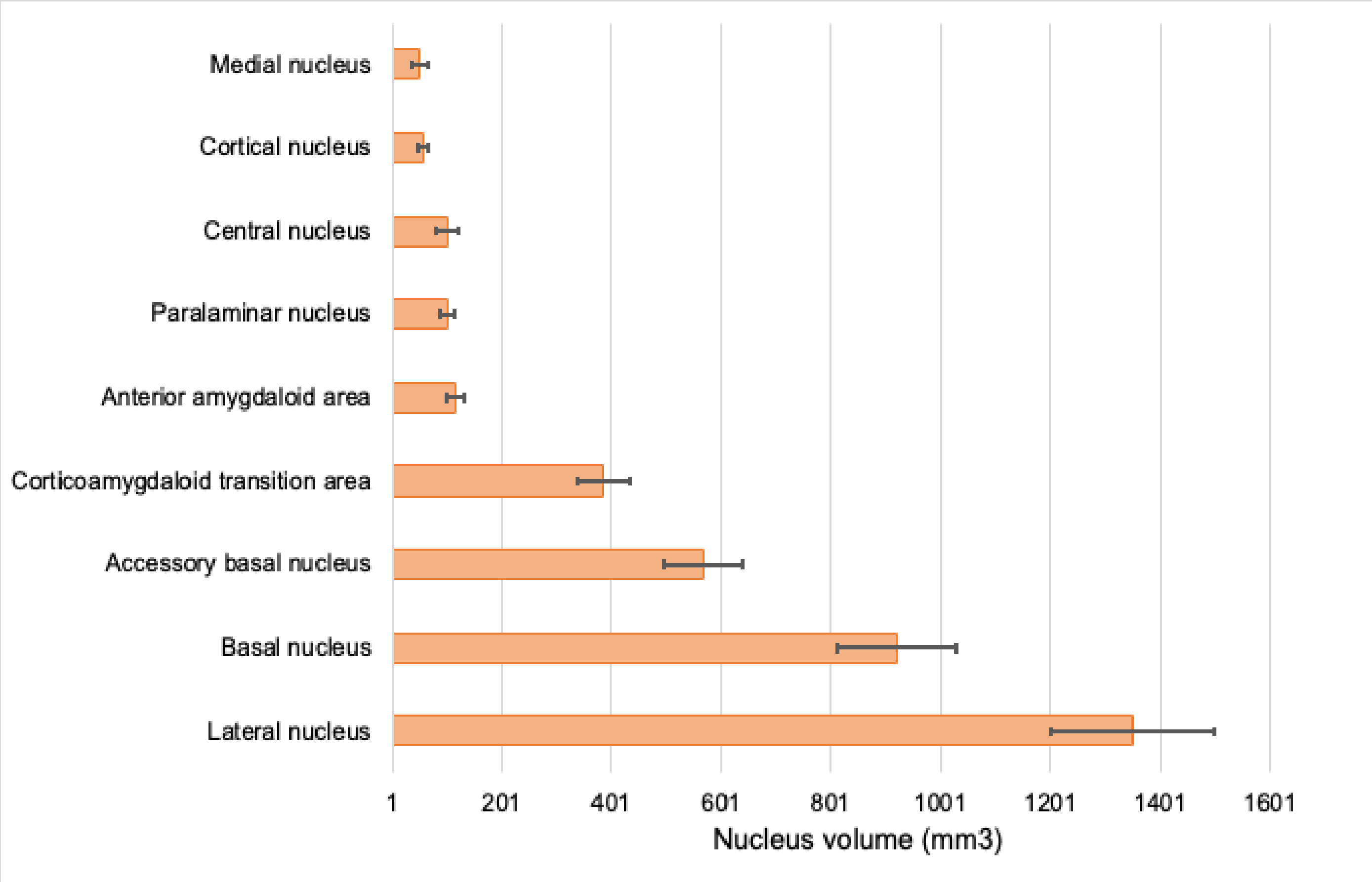

Figure S1: Distribution of amygdala nuclei volumes

The x-axis shows the volume in cubic millimetres per nucleus, as indicated on the y-axis. The orange bars represent the mean volume per nucleus across study participants and the grey error bars reflect the standard deviation

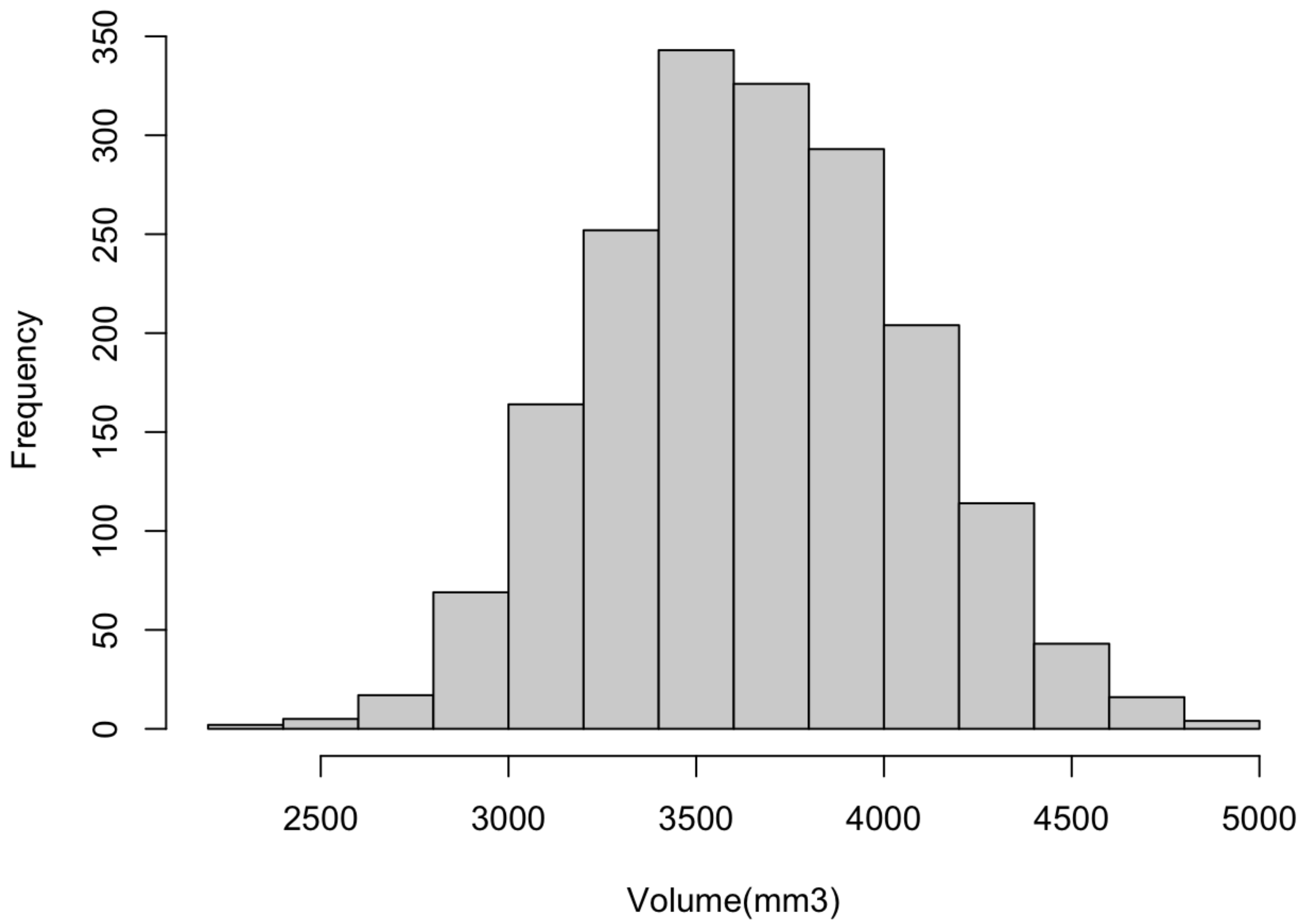

Figure S2: Distribution of whole amygdala volume

The x-axis shows amygdala volume in cubic millimetres. The y-axis shows the frequency

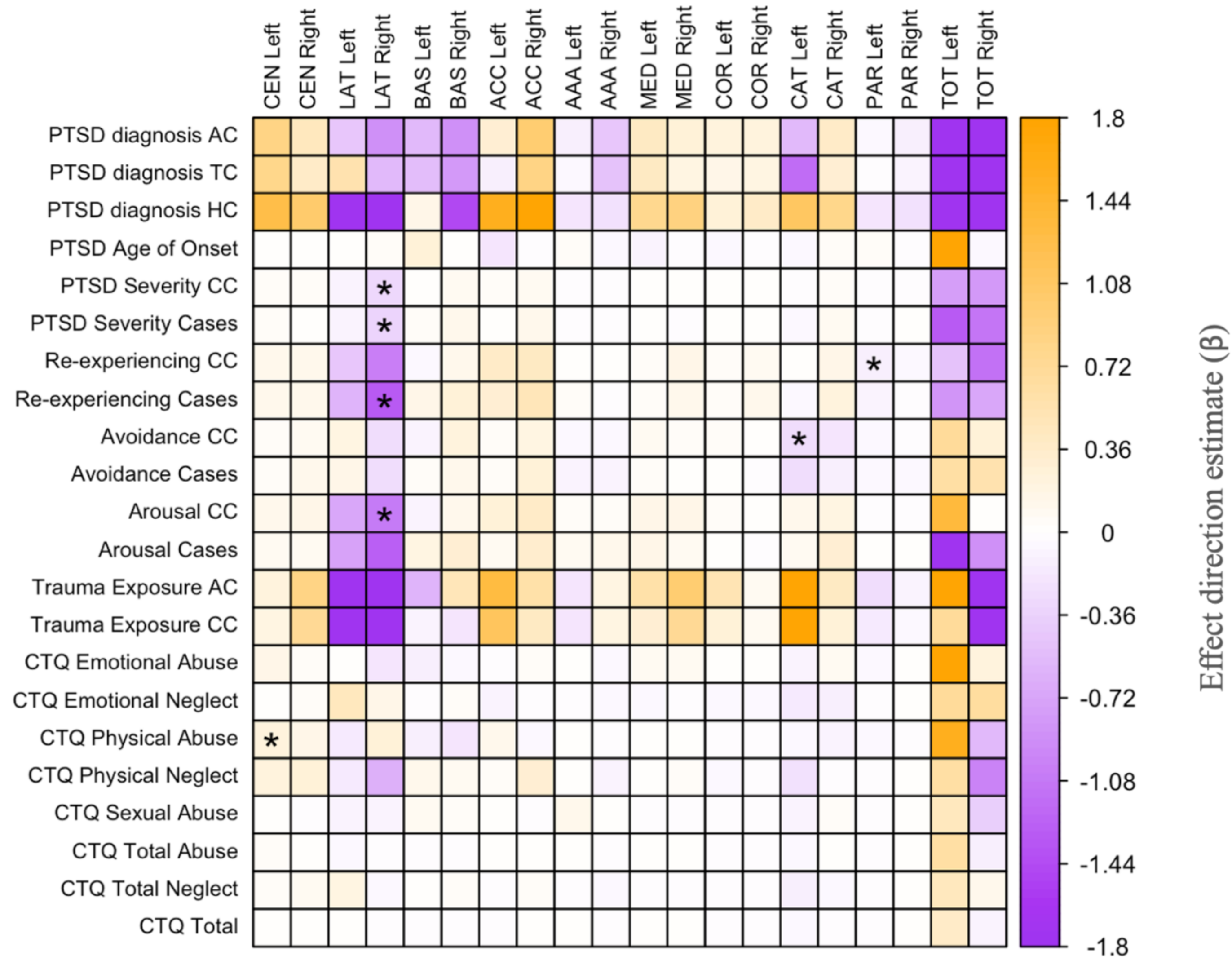

Figure S3: Comparison of amygdala nuclei volumes across PTSD diagnosis and related measures, without ComBat-GAM harmonization

The x-axis displays the amygdala nuclei for both the left and right hemispheres. The y-axis displays posttraumatic stress disorder (PTSD) diagnosis compared to all controls (AC), trauma-exposed controls (TC) and trauma-unexposed controls (HC); PTSD age of onset; PTSD severity scores as measured with the CAPS-IV; PTSD DSM-IV symptom cluster scores (re-experiencing, avoidance and arousal), and Childhood Trauma Questionnaire (CTQ) derived subscales (emotional abuse and neglect; physical abuse and neglect; sexual abuse; combined abuse, combined neglect and total scores). The colour bars indicate the effect direction estimate (β), ranging from larger volumes in blue to smaller volumes in red. The beta estimates have been capped at 1.8 and -1.8. Significant findings at a $p<0.05$ after FDR correction are indicated by an asterisk (*). CEN= central nucleus, LAT= lateral nucleus, BAS= basal nucleus, ACC= accessory basal nucleus, AAA= anterior amygdaloid area, MED= medial nucleus, COR= cortical nucleus, CAT= corticoamygdaloid transition area, PAR= paralaminar nucleus.

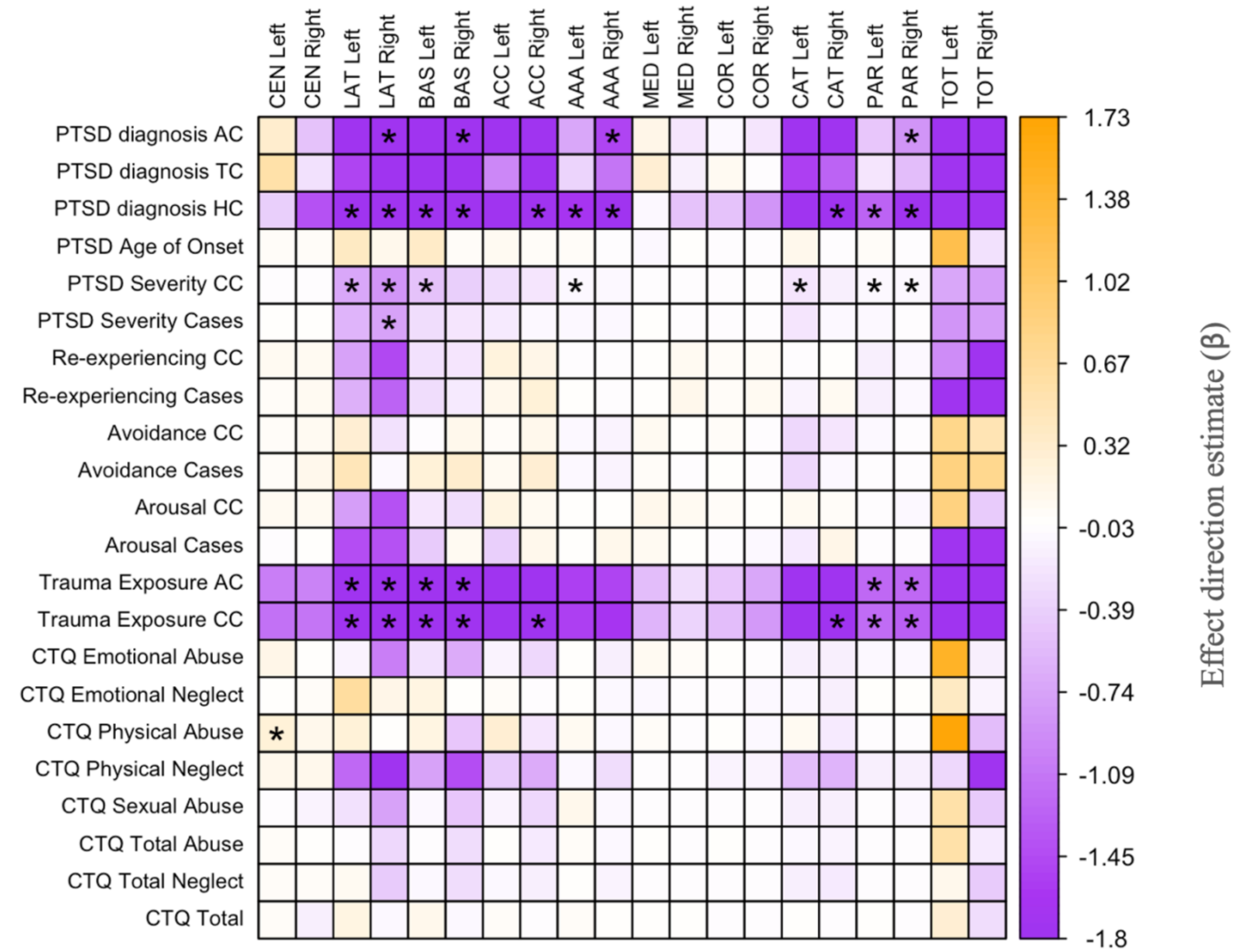

Figure S4: Comparison of amygdala nuclei volumes across PTSD diagnosis and related measures, without the correction for whole amygdala volume

The x-axis displays the amygdala nuclei for both the left and right hemispheres. The y-axis displays posttraumatic stress disorder (PTSD) diagnosis compared to all controls (AC), trauma-exposed controls (TC) and trauma-unexposed controls (HC); PTSD age of onset; PTSD severity scores as measured with the CAPS-IV; PTSD DSM-IV symptom cluster scores (re-experiencing, avoidance and arousal), and Childhood Trauma Questionnaire (CTQ) derived subscales (emotional abuse and neglect; physical abuse and neglect; sexual abuse; combined abuse, combined neglect and total scores). The colour bars indicate the effect direction estimate (β), ranging from larger volumes in blue to smaller volumes in red. The beta estimates have been capped at 1.8 and -1.8. Significant findings at a $p<0.05$ after FDR correction are indicated by an asterisk (*). CEN= central nucleus, LAT= lateral nucleus, BAS= basal nucleus, ACC= accessory basal nucleus, AAA= anterior amygdaloid area, MED= medial nucleus, COR= cortical nucleus, CAT= corticoamygdaloid transition area, PAR= paralaminar nucleus.
