# Supplementary material for: Shared and specific associations of amygdala nuclei volumes with PTSD symptom domains and childhood trauma: An ENIGMA-PGC PTSD mega-analysis
Source: Mol Psychiatry. 2026 Jul 2;31(11):6488–97. doi: 10.1038/s41380-026-03718-w (PMC13668723; doi:10.1038/s41380-026-03718-w)
Supplement: Supplementary file 2 [file 41380_2026_3718_MOESM2_ESM.pdf]

Table of contents:

Worksheet:	Short title	Description:
T1	Cohort information before and after quality control	Description of contributing cohorts before and after quality control
T2	Quality_control	The number of participants remaining after each step of quality control
T3	Amygdala_nuclei_volumes	Summary of amygdala nuclei volumes (mm^3) across PTSD cases and controls, prior to harmonization
T4	no_Combat~ICV+TOT+Site	Output from regression models for each predictor. Raw volumes ~ Predictor† + Sex + Age2 + Age + ICV + Whole left amygdala volume+Site
T5	Combat~ICV	Output from regression models for each predictor. ComBat-Gam harmonized volumes ~ Predictor† + Sex + Age2 + Age + ICV
T6	Combat~ICV+TOT	Output from regression models for each predictor. ComBat-Gam harmonized volumes ~ Predictor† + Sex + Age2 + Age + ICV + Whole left amygdala volume

Glossary (alphabetical)

AAA	Anterior Amygdaloid Area Volume	CAPS	Clinician-Administered PTSD Scale for the Diagnostic and Statistical Manual of Mental Disorders (version 4 or 5)
ABUSE	Sum of Emotional, Physical and Sexual Abuse scores	LAT	Lateral Nucleus Volume
AC	All controls	MED	Medial Nucleus Volume
ACC	Accessory Basal Nucleus Volume	NEGLECT	Sum of Emotional and Physical Neglect scores
adj R^2	Adjusted R Squared Value	P-value	Unadjusted Raw P-value
AIC	Akaike Information Criterion	PAR	Paralaminar Nucleus Volume
AOO	Age of Onset	pFDR	P-value Adjusted For Multiple Testing Using The False Discory Rate Method
BAS	Basal Nucleus Volume	PTSD	Post-traumatic Stress Disorder
Beta	Effect Estimate	ROI	Region of Interest
HC	Healthy trauma-unexposed controls	SD	Standard Deviation
CAT	Cortico-amygdaloid Transition Area Volume	SE	Standard Error
CEN	Central Nucleus Volume	t	T-test Statistic
COR	Cortical Nucleus Volume	TC	Trauma-exposed controls
CTQ	Childhood Trauma Questionaire	TOT	Whole Amygdala Volume
DF	Degrees of Freedom	TOTAL	Sum of all CTQ related Abuse and Neglect scores
DX	Diagnosis		

Table T1a: Cohort information before quality control

			Whole cohort		Cases		Controls					Age	
Site	Institution	Principal investigator	n	% male	n Cases	% male	n controls	% male	Trauma type	Scanner	Ancestry	Mean	SD
1	Duke University	Rajendra Morey	352	78.41	106	83.96	246	76.64	Military	GE MR750<br>GE EXCITE HD 3T<br>GE 4T LX Nvi<br>Philips Ingenia	Mixed (African and European)	39.22	10.23
2	Stanford University	Amit Etkin	171	53.20	82	54.90	89	51.70	Military (56%), Civilian (44%)	GE MR750	Mixed (Asian, European, Other)	35.20	10.80
3	University of Washington	Katie McLaughlin	149	75.00	33	54.55	116	49.14	Civilian	Siemens Tim Trio<br>Philips Achieva	Mixed (African, Asian, European, Mixed ancestry, Other)	12.82	2.65
4	University of South Dakota	Lee Baugh	123	80.49	78	78.21	45	84.44	Military	Siemens Skyra	Mixed (African, Asian, European, Mixed ancestry, Other)	29.14	7.01
5	Duke University-De Bellis	Michael De Bellis	117	46.61	32	46.88	85	46.51	Civilian	Siemens Tim Trio	Mixed (African, European, Other)	10.39	2.61
6	Utrecht University	Elbert Geuze	104	100.00	54	100.00	50	100.00	Military	Philips Achieva	European	36.13	9.84
7	University of the Chinese Academy of Sciences-Beijing	Li Wang	88	43.18	41	34.15	47	51.06	Civilian - Earthquake Survivors	Philips Achieva	Asian	48.14	9.97
8	University of Wisconsin at Milwaukee-Larson	Christine Larson	65	48.57	20	52.38	45	46.94	Civilian - Motor Vehicle Accident Survivors	GE MR750 Signa Excite	Mixed (African, Asian, European, Other)	33.29	10.77
9	University of Illinois-Chicago	K. Luan Phan	44	100.00	24	100.00	20	100.00	Military	GE Signa	Mixed (African, Asian, European)	31.94	8.27
10	Vanderbilt University	Jennifer Blackford	50	82.00	15	93.33	35	77.14	Military	Philips Intera	Mixed (African, European, Other)	31.34	4.63
11	Columbia University	Xi Zhu, Yuval Neria	82	34.88	49	34.62	33	35.29	Civilian	GE SIGNA EXCITE	Mixed (African, Asian, European, Other)	35.98	9.79
12	University of Western Ontario	Ruth Lanius	29	80.49	16	78.21	13	84.44	Civilian	Siemens Tim Trio	Mixed (African, Mixed ancestry, Other)	29.14	7.01
13	Harvard University-Rosso	Isabelle Rosso	95	64.29	21	42.31	74	47.67	Civilian	Siemens Tim Trio	Mixed (African, Asian, European, Other)	33.95	8.63
14	Waco Veteran's Affairs	Geoff May	98	88.78	67	88.06	31	90.32	Military	Philips Achieva	Mixed (African, Asian, European, Mixed ancestry, Other)	40.77	10.56
15	Harvard University-McLean	Kerry Ressler	67	0.00	47	0.00	20	0.00	Civilian	Siemens Tim Trio	Mixed (African, Asian, European, Other)	34.80	12.68
16	University of Wisconsin at Madison-Grupe	Dan Grupe	57	100.00	19	100.00	38	100.00	Military	GE X750 Discovery	Mixed (African, European, Other)	30.44	6.22
17	University of Mannheim	Christian Schmaal	49	0.00	49	0.00	0	0.00	Civilian	Siemens Tim Trio	European	35.86	11.64
18	Ghent University	Sven Müller	67	0.00	8	0.00	59	0.00	Civilian	Siemens Tim Trio	European	37.09	12.13
19	Minneapolis Veteran's Affairs	Seth Disner	66	92.42	36	97.22	30	86.67	Military	Siemens Tim Trio	Mixed (African, Asian, European, Mixed ancestry)	32.17	7.39
20	University of Groningen	Judith K. Daniels	40	0.00	40	0.00	0	0.00	Civilian	Siemens Tim Trio	European	38.18	9.69
21	University of Münster	Thomas Straube	47	17.46	21	0.00	26	26.19	Civilian - Intimate Partner Violence	Siemens Magnetom Prisma	European	26.71	7.11
22	Yale University	Ilan Harpaz-Rotem	70	82.19	22	82.61	48	82.00	Military	Siemens Tim Trio	Mixed (African, Asian, European, Other)	30.06	7.86
Total			2030	58.72	880	56.40	1150	60.60					

Tabel T1b: Cohort information after quality control

			Whole cohort		Cases		Controls					Age	
Site	Institution	Principal investigator	n	% male	n Cases	% male	n controls	% male	Trauma type	Scanner	Ancestry	Mean	SD
1	Duke University	Rajendra Morey	352	78.4	106	84	246	76	Military	GE MR750<br>GE EXCITE HD 3T<br>GE 4T LX Nvi<br>Philips Ingenia	Mixed (African and European)	39.2	10.2
2	Stanford University	Amit Etkin	62	69.4	14	100	48	60.4	Civilian	GE MR750	Mixed (Asian, European, Other)	32.5	10.4
3	University of Washington	Katie McLaughlin	148	50	33	54.5	115	48.7	Civilian	Siemens Tim Trio<br>Philips Achieva	Mixed (African, Asian, European, Mixed ancestry, Other)	12.8	2.67
4	University of South Dakota	Lee Baugh	121	80.2	76	77.6	45	84.4	Military	Siemens Skyra	Mixed (African, Asian, European, Mixed ancestry, Other)	29.1	7.06
5	Duke University-De Bellis	Michael De Bellis	112	47.3	28	50	84	46.4	Civilian	Siemens Tim Trio	Mixed (African, European, Other)	10.6	2.63
6	Utrecht University	Elbert Geuze	103	100	53	100	50	100	Military	Philips Achieva	European	36.1	9.69
7	University of the Chinese Academy of Sciences-Beijing	Li Wang	88	43.2	41	34.1	47	51.1	Civilian - Earthquake Survivors	Philips Achieva	Asian	48.1	9.97
8	University of Wisconsin at Milwaukee-Larson	Christine Larson	65	49.2	20	50	45	48.9	Civilian - Motor Vehicle Accident Survivors	GE MR750 Signa Excite	Mixed (African, Asian, European, Other)	33.2	11
9	University of Illinois-Chicago	K. Luan Phan	44	100	24	100	20	100	Military	GE Signa	Mixed (African, Asian, European)	31.7	8.22
10	Vanderbilt University	Jennifer Blackford	50	82	15	93.3	35	77.1	Military	Philips Intera	Mixed (African, European, Other)	31.3	4.63
11	Columbia University	Xi Zhu, Yuval Neria	56	23.2	32	21.9	24	25	Civilian	GE SIGNA EXCITE	Mixed (African, Asian, European, Other)	36.9	9.93
12	University of Western Ontario	Ruth Lanius	0	0.00	0	0.00	0	0.00	Civilian	Siemens Tim Trio	N/A	NA	NA
13	Harvard University-Rosso	Isabelle Rosso	95	46.3	21	42.9	74	47.3	Civilian	Siemens Tim Trio	Mixed (African, Asian, European, Other)	34.3	8.85
14	Waco Veteran's Affairs	Geoff May	98	88.8	67	88.1	31	90.3	Military	Philips Achieva	Mixed (African, Asian, European, Mixed ancestry, Other)	40.8	10.6
15	Harvard University-McLean	Kerry Ressler	65	0	46	0	19	0	Civilian	Siemens Tim Trio	Mixed (African, Asian, European, Other)	35	12.5
16	University of Wisconsin at Madison-Grupe	Dan Grupe	56	92.9	19	84.2	37	97.3	Military	GE X750 Discovery	Mixed (African, European, Other)	30.4	6.33
17	University of Mannheim	Christian Schmaal	49	0	49	0	0	0	Civilian	Siemens Tim Trio	European	35.9	11.6
18	Ghent University	Sven Müller	67	0	8	0	59	0	Civilian	Siemens Tim Trio	European	37.1	12.1
19	Minneapolis Veteran's Affairs	Seth Disner	66	92.4	36	97.2	30	86.7	Military	Siemens Tim Trio	Mixed (African, Asian, European, Mixed ancestry)	32.2	7.39
20	University of Groningen	Judith K. Daniels	40	0	40	0	0	0	Civilian	Siemens Tim Trio	European	38.2	9.69
21	University of Münster	Thomas Straube	47	10.6	21	0	26	19.2	Civilian - Intimate Partner Violence	Siemens Magnetom Prisma	European	26.9	7.13
22	Yale University	Ilan Harpaz-Rotem	68	83.8	22	86.4	46	82.6	Military	Siemens Tim Trio	Mixed (African, Asian, European, Other)	30.3	7.89
Total			1852	60.48	771	58.90	1081	61.60					

The number of participants remaining after quality control

Filtering steps	Excluded	Total	Cases	Controls
START:	-	2030	887	1143
Remove participants with missing covariates and failed amygdala nuclei segmentation	141	1889	795	1094
Remove participants with Euler number outliers (4SD)	31	1858	774	1084
Remove participants with ICV outliers (4SD)	0	1858	774	1084
Remove participants with amygdala nuclei volume outliers for each hemisphere (4SD)	6	1852	771	1081
Total	178	1852	771	1081

Summary of amygdala nuclei volumes (mm³) prior to harmonization

Hemisphere	ROI	Mean	Median	SD	Minimum	Maximum
Left	CEN	48.23	47.69	10.10	21.42	83.32
	LAT	662.36	659.76	77.19	376.73	927.20
	BAS	451.76	448.98	55.59	268.89	646.74
	ACC	277.50	276.13	36.98	167.93	395.36
	AAA	55.86	55.38	8.61	32.26	85.14
	MED	24.79	23.83	7.79	7.12	50.33
	COR	28.19	28.07	5.37	7.89	49.14
	CAT	190.81	189.87	26.20	117.79	275.79
	PAR	50.95	50.56	6.51	29.79	76.15
	TOT	1790.44	1781.89	206.92	1100.54	2451.64
Right	CEN	52.84	52.07	10.82	21.10	92.11
	LAT	688.72	686.37	78.90	425.55	958.08
	BAS	468.70	468.23	57.07	308.44	654.84
	ACC	290.48	290.69	37.74	170.36	412.36
	AAA	60.89	60.69	9.17	35.19	94.28
	MED	26.69	26.07	7.77	9.23	51.88
	COR	29.79	29.49	5.06	14.00	46.33
	CAT	195.47	193.87	25.13	128.37	291.32
	PAR	51.48	51.14	6.38	33.10	73.36
	TOT	1865.05	1863.62	214.16	1223.01	2509.08
Bilateral	CEN	101.07	99.98	19.16	44.60	161.67
	LAT	1351.08	1347.96	149.81	802.28	1842.52
	BAS	920.46	917.90	108.49	577.33	1301.58
	ACC	567.98	566.76	71.58	347.14	790.23
	AAA	116.74	116.11	16.51	70.99	178.68
	MED	51.47	50.33	14.29	20.60	98.40
	COR	57.98	57.69	9.65	29.74	92.18
	CAT	386.28	383.48	48.79	255.55	553.96
	PAR	102.43	101.94	12.33	62.90	145.84
	TOT	3655.49	3640.78	408.23	2329.68	4899.94

Table T4: Output from regression models for each predictor (Raw volumes ~ Predictor† + Sex + Age2 + Age + ICV + Whole left amygdala volume+Site)

	Participants	ROI	Hemisphere	Beta	SE	t	P-value	pBON	DF	adj R^2
PTSD Diagnosis	PTSD cases (n=771) and all controls (n=1081)	CEN	Left	0.83	0.36	2.32	2.03E-02	4.06E-01	1819.00	0.54
			Right	0.44	0.37	1.18	2.38E-01	4.77E+00	1819.00	0.57
		LAT	Left	-0.46	1.45	-0.31	7.54E-01	1.51E+01	1819.00	0.87
			Right	-0.90	1.43	-0.63	5.29E-01	1.06E+01	1819.00	0.88
		BAS	Left	-0.57	0.62	-0.92	3.59E-01	7.18E+00	1819.00	0.95
			Right	-0.87	0.64	-1.35	1.78E-01	3.55E+00	1819.00	0.95
		ACC	Left	0.30	0.70	0.44	6.62E-01	1.32E+01	1819.00	0.87
			Right	1.01	0.67	1.51	1.32E-01	2.63E+00	1819.00	0.89
		AAA	Left	-0.13	0.27	-0.47	6.39E-01	1.28E+01	1819.00	0.65
			Right	-0.44	0.28	-1.57	1.16E-01	2.32E+00	1819.00	0.66
		MED	Left	0.43	0.30	1.41	1.60E-01	3.19E+00	1819.00	0.45
			Right	0.27	0.30	0.89	3.72E-01	7.43E+00	1819.00	0.45
		COR	Left	0.22	0.19	1.20	2.29E-01	4.58E+00	1819.00	0.56
			Right	0.23	0.17	1.36	1.75E-01	3.51E+00	1819.00	0.60
		CAT	Left	-0.57	0.59	-0.97	3.30E-01	6.61E+00	1819.00	0.82
			Right	0.39	0.54	0.72	4.70E-01	9.40E+00	1819.00	0.83
		PAR	Left	-0.07	0.14	-0.48	6.33E-01	1.27E+01	1819.00	0.82
			Right	-0.12	0.14	-0.86	3.92E-01	7.84E+00	1819.00	0.82
		TOT	Left	-16.58	7.82	-2.12	3.41E-02	6.81E-01	1820.00	0.48
			Right	-24.07	8.02	-3.00	2.72E-03	5.44E-02	1820.00	0.49
	PTSD cases (n=771) and healthy controls (n=271)	CEN	Left	1.28	0.59	2.16	3.09E-02	6.17E-01	1040.00	0.53
			Right	1.04	0.59	1.75	8.07E-02	1.61E+00	1040.00	0.58
		LAT	Left	-4.76	2.35	-2.03	4.28E-02	8.56E-01	1040.00	0.88
			Right	-3.08	2.24	-1.38	1.69E-01	3.38E+00	1040.00	0.89
		BAS	Left	0.18	0.97	0.18	8.57E-01	1.71E+01	1040.00	0.96
			Right	-1.46	1.04	-1.40	1.62E-01	3.25E+00	1040.00	0.96
		ACC	Left	1.58	1.11	1.42	1.57E-01	3.14E+00	1040.00	0.87
			Right	1.87	1.05	1.79	7.42E-02	1.48E+00	1040.00	0.90
		AAA	Left	-0.20	0.43	-0.45	6.50E-01	1.30E+01	1040.00	0.66
			Right	-0.22	0.44	-0.49	6.23E-01	1.25E+01	1040.00	0.69
		MED	Left	0.79	0.49	1.61	1.08E-01	2.17E+00	1040.00	0.42
			Right	0.88	0.48	1.81	7.11E-02	1.42E+00	1040.00	0.43
		COR	Left	0.26	0.31	0.83	4.07E-01	8.13E+00	1040.00	0.52
			Right	0.38	0.27	1.39	1.64E-01	3.28E+00	1040.00	0.59
		CAT	Left	1.08	0.94	1.15	2.50E-01	5.00E+00	1040.00	0.82
			Right	0.82	0.86	0.96	3.35E-01	6.70E+00	1040.00	0.84
		PAR	Left	-0.20	0.22	-0.91	3.65E-01	7.29E+00	1040.00	0.84
			Right	-0.23	0.23	-0.97	3.32E-01	6.65E+00	1040.00	0.83
		TOT	Left	-18.80	12.85	-1.46	1.44E-01	2.87E+00	1041.00	0.48
			Right	-38.57	12.98	-2.97	3.04E-03	6.09E-02	1041.00	0.51
	PTSD cases (n=771) and trauma-exposed controls (n=780)	CEN	Left	0.80	0.38	2.09	3.66E-02	7.32E-01	1548.00	0.53
			Right	0.37	0.40	0.93	3.55E-01	7.10E+00	1548.00	0.57
		LAT	Left	0.56	1.55	0.36	7.17E-01	1.43E+01	1548.00	0.87
			Right	-0.54	1.53	-0.35	7.23E-01	1.45E+01	1548.00	0.88
		BAS	Left	-0.52	0.67	-0.78	4.36E-01	8.73E+00	1548.00	0.95
			Right	-0.82	0.68	-1.20	2.32E-01	4.64E+00	1548.00	0.95

	PTSD cases (n=771) and trauma-exposed controls (n=780)	ACC	Left	-0.14	0.74	-0.19	8.48E-01	1.70E+01	1548.00	0.87
			Right	0.85	0.71	1.20	2.32E-01	4.64E+00	1548.00	0.89
		AAA	Left	-0.04	0.28	-0.14	8.90E-01	1.78E+01	1548.00	0.64
			Right	-0.48	0.30	-1.60	1.09E-01	2.18E+00	1548.00	0.66
		MED	Left	0.40	0.32	1.26	2.08E-01	4.17E+00	1548.00	0.45
			Right	0.21	0.32	0.66	5.07E-01	1.01E+01	1548.00	0.47
		COR	Left	0.15	0.20	0.73	4.64E-01	9.27E+00	1548.00	0.57
			Right	0.20	0.18	1.12	2.62E-01	5.23E+00	1548.00	0.60
		CAT	Left	-1.19	0.62	-1.90	5.77E-02	1.15E+00	1548.00	0.82
			Right	0.30	0.58	0.51	6.07E-01	1.21E+01	1548.00	0.83
		PAR	Left	-0.02	0.15	-0.16	8.75E-01	1.75E+01	1548.00	0.82
			Right	-0.10	0.15	-0.63	5.28E-01	1.06E+01	1548.00	0.81
		TOT	Left	-16.15	8.28	-1.95	5.12E-02	1.02E+00	1549.00	0.48
			Right	-20.62	8.58	-2.40	1.63E-02	3.26E-01	1549.00	0.48
Trauma exposure	Healthy controls (n=271) and trauma-exposed controls (n=780)	CEN	Left	0.23	0.60	0.39	6.98E-01	1.40E+01	1020.00	0.55
			Right	0.84	0.63	1.33	1.83E-01	3.65E+00	1020.00	0.55
		LAT	Left	-4.07	2.44	-1.67	9.51E-02	1.90E+00	1020.00	0.86
			Right	-3.52	2.46	-1.43	1.52E-01	3.05E+00	1020.00	0.87
		BAS	Left	-0.61	1.06	-0.57	5.66E-01	1.13E+01	1020.00	0.95
			Right	0.50	1.09	0.46	6.46E-01	1.29E+01	1020.00	0.95
		ACC	Left	1.32	1.18	1.12	2.62E-01	5.23E+00	1020.00	0.87
			Right	0.60	1.14	0.52	6.01E-01	1.20E+01	1020.00	0.88
		AAA	Left	-0.18	0.45	-0.41	6.82E-01	1.36E+01	1020.00	0.64
			Right	0.19	0.49	0.40	6.92E-01	1.38E+01	1020.00	0.63
		MED	Left	0.60	0.52	1.15	2.49E-01	4.97E+00	1020.00	0.46
			Right	0.98	0.50	1.95	5.16E-02	1.03E+00	1020.00	0.46
		COR	Left	0.52	0.30	1.70	8.95E-02	1.79E+00	1020.00	0.58
			Right	0.08	0.28	0.27	7.90E-01	1.58E+01	1020.00	0.59
		CAT	Left	2.46	1.00	2.46	1.39E-02	2.78E-01	1020.00	0.81
			Right	0.42	0.92	0.46	6.49E-01	1.30E+01	1020.00	0.82
		PAR	Left	-0.26	0.25	-1.06	2.90E-01	5.81E+00	1020.00	0.80
			Right	-0.09	0.24	-0.37	7.12E-01	1.42E+01	1020.00	0.80
		TOT	Left	4.44	13.03	0.34	7.33E-01	1.47E+01	1021.00	0.47
			Right	-5.99	13.36	-0.45	6.54E-01	1.31E+01	1021.00	0.47
	PTSD cases (n=771) and trauma-exposed contols (n=780) vs healthy controls (n=271)	CEN	Left	0.18	0.55	0.33	7.43E-01	1.49E+01	1788.00	0.54
			Right	0.75	0.57	1.32	1.85E-01	3.71E+00	1788.00	0.57
		LAT	Left	-4.01	2.23	-1.80	7.28E-02	1.46E+00	1788.00	0.87
			Right	-2.18	2.19	-0.99	3.20E-01	6.40E+00	1788.00	0.88
		BAS	Left	-0.10	0.96	-0.11	9.13E-01	1.83E+01	1788.00	0.95
			Right	-0.18	0.99	-0.19	8.53E-01	1.71E+01	1788.00	0.95
		ACC	Left	1.15	1.07	1.07	2.86E-01	5.72E+00	1788.00	0.87
			Right	0.41	1.02	0.40	6.91E-01	1.38E+01	1788.00	0.89
		AAA	Left	-0.18	0.41	-0.44	6.57E-01	1.31E+01	1788.00	0.65
			Right	0.19	0.43	0.45	6.55E-01	1.31E+01	1788.00	0.66
		MED	Left	0.32	0.47	0.69	4.93E-01	9.86E+00	1788.00	0.45
			Right	0.75	0.46	1.63	1.02E-01	2.05E+00	1788.00	0.46
		COR	Left	0.27	0.29	0.92	3.56E-01	7.11E+00	1788.00	0.56
			Right	0.08	0.26	0.30	7.63E-01	1.53E+01	1788.00	0.60

		CAT	Left	2.54	0.90	2.82	4.93E-03	9.86E-02	1788.00	0.82
			Right	0.25	0.83	0.30	7.62E-01	1.52E+01	1788.00	0.83
		PAR	Left	-0.15	0.22	-0.69	4.90E-01	9.80E+00	1788.00	0.82
			Right	-0.07	0.22	-0.32	7.52E-01	1.50E+01	1788.00	0.82
		TOT	Left	0.69	12.08	0.06	9.54E-01	1.91E+01	1789.00	0.47
			Right	-13.30	12.37	-1.08	2.83E-01	5.65E+00	1789.00	0.48
PTSD age of onset	PTSD cases (n=150)	CEN	Left	0.01	0.06	0.12	9.06E-01	1.81E+01	134.00	0.57
			Right	0.01	0.06	0.20	8.45E-01	1.69E+01	134.00	0.62
		LAT	Left	0.03	0.26	0.12	9.04E-01	1.81E+01	134.00	0.87
			Right	0.04	0.28	0.14	8.92E-01	1.78E+01	134.00	0.87
		BAS	Left	0.26	0.12	2.22	2.82E-02	5.65E-01	134.00	0.95
			Right	0.00	0.12	0.03	9.80E-01	1.96E+01	134.00	0.95
		ACC	Left	-0.19	0.12	-1.54	1.26E-01	2.53E+00	134.00	0.87
			Right	-0.01	0.12	-0.06	9.50E-01	1.90E+01	134.00	0.88
		AAA	Left	0.05	0.05	0.92	3.58E-01	7.16E+00	134.00	0.61
			Right	-0.05	0.05	-0.85	3.95E-01	7.89E+00	134.00	0.65
		MED	Left	-0.08	0.06	-1.39	1.67E-01	3.34E+00	134.00	0.45
			Right	-0.02	0.05	-0.35	7.27E-01	1.45E+01	134.00	0.40
		COR	Left	-0.05	0.04	-1.36	1.78E-01	3.55E+00	134.00	0.54
			Right	-0.02	0.03	-0.57	5.69E-01	1.14E+01	134.00	0.65
		CAT	Left	-0.07	0.11	-0.60	5.52E-01	1.10E+01	134.00	0.79
			Right	0.04	0.10	0.39	7.00E-01	1.40E+01	134.00	0.83
		PAR	Left	0.04	0.02	1.63	1.06E-01	2.11E+00	134.00	0.83
			Right	0.00	0.03	-0.17	8.67E-01	1.73E+01	134.00	0.78
		TOT	Left	1.92	1.42	1.35	1.79E-01	3.57E+00	135.00	0.45
			Right	-0.06	1.45	-0.04	9.68E-01	1.94E+01	135.00	0.48
PTSD severity scores (CAPS-IV)	PTSD cases (n=215) and all controls (n=261)	CEN	Left	0.05	0.02	2.69	7.39E-03	1.48E-01	457.00	0.64
			Right	0.04	0.02	1.98	4.87E-02	9.75E-01	457.00	0.61
		LAT	Left	-0.09	0.08	-1.14	2.57E-01	5.14E+00	457.00	0.87
			Right	-0.29	0.08	-3.65	2.94E-04	5.89E-03	457.00	0.88
		BAS	Left	0.00	0.03	0.00	9.96E-01	1.99E+01	457.00	0.96
			Right	0.10	0.04	2.57	1.06E-02	2.11E-01	457.00	0.95
		ACC	Left	0.05	0.04	1.17	2.43E-01	4.86E+00	457.00	0.87
			Right	0.09	0.04	2.29	2.22E-02	4.45E-01	457.00	0.89
		AAA	Left	-0.01	0.01	-0.92	3.57E-01	7.14E+00	457.00	0.62
			Right	0.00	0.02	-0.27	7.86E-01	1.57E+01	457.00	0.57
		MED	Left	0.03	0.02	1.90	5.80E-02	1.16E+00	457.00	0.46
			Right	0.03	0.02	1.69	9.12E-02	1.82E+00	457.00	0.50
		COR	Left	0.01	0.01	1.01	3.13E-01	6.26E+00	457.00	0.56
			Right	0.01	0.01	1.18	2.38E-01	4.76E+00	457.00	0.64
		CAT	Left	-0.02	0.03	-0.67	5.04E-01	1.01E+01	457.00	0.81
			Right	0.04	0.03	1.12	2.65E-01	5.29E+00	457.00	0.82
		PAR	Left	-0.01	0.01	-1.82	6.92E-02	1.38E+00	457.00	0.82
			Right	0.00	0.01	-0.26	7.94E-01	1.59E+01	457.00	0.80
		TOT	Left	-0.78	0.45	-1.74	8.30E-02	1.66E+00	458.00	0.48
			Right	-0.80	0.44	-1.82	6.93E-02	1.39E+00	458.00	0.51
	PTSD cases (n=215)	CEN	Left	0.04	0.03	1.58	1.15E-01	2.30E+00	204.00	0.53
			Right	0.02	0.03	0.74	4.62E-01	9.23E+00	204.00	0.53

	PTSD cases (n=215)	LAT	Left	-0.09	0.11	-0.82	4.13E-01	8.26E+00	204.00	0.85
			Right	-0.33	0.10	-3.27	1.27E-03	2.54E-02	204.00	0.89
		BAS	Left	0.07	0.04	1.59	1.13E-01	2.27E+00	204.00	0.96
			Right	0.12	0.05	2.54	1.19E-02	2.38E-01	204.00	0.95
		ACC	Left	0.03	0.05	0.59	5.58E-01	1.12E+01	204.00	0.87
			Right	0.11	0.05	2.19	2.96E-02	5.91E-01	204.00	0.89
		AAA	Left	-0.01	0.02	-0.69	4.90E-01	9.80E+00	204.00	0.61
			Right	-0.01	0.02	-0.37	7.10E-01	1.42E+01	204.00	0.63
		MED	Left	0.01	0.03	0.51	6.12E-01	1.22E+01	204.00	0.34
			Right	-0.01	0.02	-0.22	8.26E-01	1.65E+01	204.00	0.41
		COR	Left	0.00	0.01	0.01	9.88E-01	1.98E+01	204.00	0.49
			Right	0.00	0.01	0.27	7.91E-01	1.58E+01	204.00	0.58
		CAT	Left	-0.05	0.05	-1.06	2.91E-01	5.82E+00	204.00	0.79
			Right	0.09	0.04	2.25	2.58E-02	5.16E-01	204.00	0.83
		PAR	Left	0.00	0.01	-0.38	7.03E-01	1.41E+01	204.00	0.81
			Right	0.01	0.01	0.68	4.98E-01	9.97E+00	204.00	0.79
		TOT	Left	-1.32	0.62	-2.15	3.30E-02	6.59E-01	205.00	0.41
			Right	-1.10	0.61	-1.78	7.60E-02	1.52E+00	205.00	0.45
PTSD subscale: Re-experiencing (CAPS-IV)	PTSD cases (n=124) and controls (n=118)	CEN	Left	0.13	0.08	1.61	1.08E-01	2.15E+00	229.00	0.53
			Right	0.14	0.08	1.74	8.31E-02	1.66E+00	229.00	0.57
		LAT	Left	-0.47	0.32	-1.45	1.49E-01	2.98E+00	229.00	0.87
			Right	-1.02	0.35	-2.92	3.85E-03	7.70E-02	229.00	0.86
		BAS	Left	-0.07	0.14	-0.49	6.24E-01	1.25E+01	229.00	0.95
			Right	0.12	0.16	0.76	4.46E-01	8.91E+00	229.00	0.94
		ACC	Left	0.37	0.16	2.31	2.20E-02	4.40E-01	229.00	0.87
			Right	0.41	0.16	2.48	1.40E-02	2.81E-01	229.00	0.87
		AAA	Left	0.01	0.06	0.24	8.11E-01	1.62E+01	229.00	0.63
			Right	0.02	0.07	0.25	8.01E-01	1.60E+01	229.00	0.56
		MED	Left	0.05	0.07	0.71	4.81E-01	9.63E+00	229.00	0.37
			Right	0.15	0.07	2.03	4.32E-02	8.64E-01	229.00	0.43
		COR	Left	0.05	0.04	1.25	2.11E-01	4.22E+00	229.00	0.50
			Right	0.07	0.04	1.95	5.23E-02	1.05E+00	229.00	0.59
		CAT	Left	0.03	0.13	0.26	7.97E-01	1.59E+01	229.00	0.83
			Right	0.16	0.14	1.13	2.59E-01	5.17E+00	229.00	0.81
		PAR	Left	-0.11	0.03	-3.31	1.07E-03	2.14E-02	229.00	0.81
			Right	-0.04	0.04	-1.19	2.37E-01	4.73E+00	229.00	0.79
		TOT	Left	-0.48	1.84	-0.26	7.93E-01	1.59E+01	230.00	0.45
			Right	-1.12	1.75	-0.64	5.25E-01	1.05E+01	230.00	0.52
	PTSD cases (n=124)	CEN	Left	0.14	0.10	1.41	1.60E-01	3.20E+00	115.00	0.49
			Right	0.14	0.10	1.43	1.56E-01	3.12E+00	115.00	0.62
		LAT	Left	-0.59	0.38	-1.55	1.25E-01	2.50E+00	115.00	0.86
			Right	-1.32	0.40	-3.27	1.44E-03	2.87E-02	115.00	0.86
		BAS	Left	0.16	0.16	0.97	3.34E-01	6.69E+00	115.00	0.96
			Right	0.28	0.18	1.51	1.35E-01	2.70E+00	115.00	0.94
		ACC	Left	0.30	0.18	1.66	9.93E-02	1.99E+00	115.00	0.88
			Right	0.48	0.19	2.53	1.26E-02	2.52E-01	115.00	0.88
		AAA	Left	0.05	0.08	0.60	5.47E-01	1.09E+01	115.00	0.56
			Right	-0.03	0.08	-0.37	7.15E-01	1.43E+01	115.00	0.59

	PTSD cases (n=124)	MED	Left	0.05	0.09	0.51	6.08E-01	1.22E+01	115.00	0.33
			Right	0.13	0.08	1.65	1.02E-01	2.04E+00	115.00	0.49
		COR	Left	0.05	0.05	1.07	2.87E-01	5.74E+00	115.00	0.50
			Right	0.11	0.04	2.61	1.03E-02	2.05E-01	115.00	0.68
		CAT	Left	-0.06	0.15	-0.43	6.66E-01	1.33E+01	115.00	0.85
			Right	0.23	0.16	1.46	1.47E-01	2.94E+00	115.00	0.83
		PAR	Left	-0.09	0.04	-2.38	1.90E-02	3.80E-01	115.00	0.84
			Right	-0.02	0.04	-0.41	6.85E-01	1.37E+01	115.00	0.78
		TOT	Left	-0.86	2.10	-0.41	6.81E-01	1.36E+01	116.00	0.48
			Right	-0.70	1.97	-0.36	7.23E-01	1.45E+01	116.00	0.57
PTSD subscale: Avoidance (CAPS-IV)	PTSD cases (n=124) and controls (n=118)	CEN	Left	0.07	0.06	1.25	2.13E-01	4.27E+00	229.00	0.53
			Right	0.11	0.06	1.90	5.85E-02	1.17E+00	229.00	0.57
		LAT	Left	0.19	0.23	0.81	4.17E-01	8.33E+00	229.00	0.87
			Right	-0.26	0.25	-1.06	2.91E-01	5.82E+00	229.00	0.85
		BAS	Left	-0.07	0.10	-0.73	4.69E-01	9.38E+00	229.00	0.95
			Right	0.22	0.11	1.95	5.25E-02	1.05E+00	229.00	0.94
		ACC	Left	0.06	0.11	0.55	5.83E-01	1.17E+01	229.00	0.86
			Right	0.18	0.12	1.57	1.18E-01	2.36E+00	229.00	0.86
		AAA	Left	-0.05	0.04	-1.22	2.23E-01	4.47E+00	229.00	0.63
			Right	-0.07	0.05	-1.37	1.71E-01	3.42E+00	229.00	0.56
		MED	Left	0.10	0.05	1.90	5.92E-02	1.18E+00	229.00	0.38
			Right	0.04	0.05	0.71	4.76E-01	9.52E+00	229.00	0.42
		COR	Left	0.05	0.03	1.53	1.27E-01	2.54E+00	229.00	0.50
			Right	0.00	0.03	0.12	9.04E-01	1.81E+01	229.00	0.59
		CAT	Left	-0.29	0.09	-3.23	1.44E-03	2.87E-02	229.00	0.84
			Right	-0.19	0.10	-1.89	5.95E-02	1.19E+00	229.00	0.81
		PAR	Left	-0.05	0.02	-2.10	3.68E-02	7.35E-01	229.00	0.81
			Right	-0.03	0.02	-1.05	2.97E-01	5.94E+00	229.00	0.79
		TOT	Left	0.68	1.29	0.53	5.97E-01	1.19E+01	230.00	0.45
			Right	0.25	1.23	0.21	8.37E-01	1.67E+01	230.00	0.51
	PTSD cases (n=124)	CEN	Left	0.05	0.07	0.73	4.67E-01	9.33E+00	115.00	0.49
			Right	0.14	0.07	2.10	3.83E-02	7.66E-01	115.00	0.63
		LAT	Left	0.15	0.27	0.57	5.72E-01	1.14E+01	115.00	0.86
			Right	-0.28	0.30	-0.95	3.46E-01	6.92E+00	115.00	0.85
		BAS	Left	0.04	0.11	0.34	7.31E-01	1.46E+01	115.00	0.96
			Right	0.12	0.13	0.92	3.59E-01	7.17E+00	115.00	0.94
		ACC	Left	0.07	0.13	0.53	5.98E-01	1.20E+01	115.00	0.88
			Right	0.28	0.13	2.06	4.17E-02	8.35E-01	115.00	0.88
		AAA	Left	-0.09	0.06	-1.60	1.13E-01	2.25E+00	115.00	0.57
			Right	-0.11	0.06	-1.91	5.92E-02	1.18E+00	115.00	0.60
		MED	Left	0.06	0.06	0.97	3.35E-01	6.70E+00	115.00	0.33
			Right	0.02	0.06	0.37	7.12E-01	1.42E+01	115.00	0.48
		COR	Left	0.03	0.04	0.85	3.98E-01	7.96E+00	115.00	0.50
			Right	-0.01	0.03	-0.43	6.70E-01	1.34E+01	115.00	0.66
		CAT	Left	-0.27	0.10	-2.73	7.28E-03	1.46E-01	115.00	0.86
			Right	-0.12	0.11	-1.04	3.00E-01	6.00E+00	115.00	0.83
		PAR	Left	-0.04	0.03	-1.51	1.35E-01	2.70E+00	115.00	0.83
			Right	-0.04	0.03	-1.32	1.89E-01	3.77E+00	115.00	0.79

		TOT	Left	0.63	1.47	0.43	6.68E-01	1.34E+01	116.00	0.48
			Right	0.55	1.39	0.40	6.91E-01	1.38E+01	116.00	0.57
PTSD subscale: Arousal (CAPS-IV)	PTSD cases (n=124) and controls (n=118)	CEN	Left	0.14	0.07	1.93	5.53E-02	1.11E+00	229.00	0.53
			Right	0.16	0.07	2.20	2.88E-02	5.76E-01	229.00	0.57
		LAT	Left	-0.71	0.29	-2.44	1.56E-02	3.11E-01	229.00	0.87
			Right	-1.08	0.31	-3.42	7.32E-04	1.46E-02	229.00	0.86
		BAS	Left	-0.09	0.13	-0.67	5.04E-01	1.01E+01	229.00	0.95
			Right	0.12	0.14	0.84	3.99E-01	7.99E+00	229.00	0.94
		ACC	Left	0.28	0.15	1.96	5.10E-02	1.02E+00	229.00	0.86
			Right	0.38	0.15	2.58	1.05E-02	2.11E-01	229.00	0.87
		AAA	Left	0.05	0.05	0.86	3.90E-01	7.80E+00	229.00	0.63
			Right	0.06	0.06	0.96	3.36E-01	6.71E+00	229.00	0.56
		MED	Left	0.15	0.07	2.21	2.82E-02	5.64E-01	229.00	0.38
			Right	0.16	0.06	2.49	1.36E-02	2.71E-01	229.00	0.44
		COR	Left	0.07	0.04	1.80	7.28E-02	1.46E+00	229.00	0.50
			Right	0.03	0.03	0.95	3.41E-01	6.82E+00	229.00	0.59
		CAT	Left	0.13	0.12	1.09	2.77E-01	5.53E+00	229.00	0.84
			Right	0.19	0.13	1.48	1.40E-01	2.80E+00	229.00	0.81
		PAR	Left	-0.02	0.03	-0.72	4.73E-01	9.46E+00	229.00	0.80
			Right	-0.03	0.03	-0.88	3.78E-01	7.56E+00	229.00	0.79
		TOT	Left	1.35	1.66	0.82	4.16E-01	8.32E+00	230.00	0.45
			Right	0.01	1.59	0.01	9.95E-01	1.99E+01	230.00	0.51
	PTSD cases (n=124)	CEN	Left	0.10	0.12	0.81	4.22E-01	8.43E+00	115.00	0.49
			Right	0.07	0.12	0.63	5.27E-01	1.05E+01	115.00	0.62
		LAT	Left	-0.74	0.47	-1.57	1.20E-01	2.40E+00	115.00	0.86
			Right	-1.29	0.51	-2.55	1.21E-02	2.42E-01	115.00	0.85
		BAS	Left	0.18	0.20	0.93	3.57E-01	7.14E+00	115.00	0.96
			Right	0.30	0.23	1.33	1.85E-01	3.71E+00	115.00	0.94
		ACC	Left	0.07	0.23	0.32	7.51E-01	1.50E+01	115.00	0.88
			Right	0.35	0.24	1.49	1.40E-01	2.80E+00	115.00	0.88
		AAA	Left	0.08	0.10	0.80	4.25E-01	8.50E+00	115.00	0.56
			Right	0.14	0.10	1.47	1.46E-01	2.91E+00	115.00	0.60
		MED	Left	0.14	0.11	1.31	1.92E-01	3.85E+00	115.00	0.34
			Right	0.10	0.10	0.97	3.33E-01	6.65E+00	115.00	0.49
		COR	Left	0.03	0.06	0.47	6.38E-01	1.28E+01	115.00	0.50
			Right	0.00	0.05	-0.08	9.36E-01	1.87E+01	115.00	0.66
		CAT	Left	0.10	0.18	0.54	5.91E-01	1.18E+01	115.00	0.85
			Right	0.31	0.20	1.58	1.17E-01	2.34E+00	115.00	0.83
		PAR	Left	0.03	0.05	0.72	4.71E-01	9.42E+00	115.00	0.83
			Right	0.02	0.05	0.36	7.22E-01	1.44E+01	115.00	0.78
		TOT	Left	-2.34	2.57	-0.91	3.65E-01	7.30E+00	116.00	0.48
			Right	-0.89	2.42	-0.37	7.13E-01	1.43E+01	116.00	0.57
Emotional Abuse	PTSD cases (n=187) and controls (n=246)	CEN	Left	0.15	0.06	2.56	0.01	2.17E-01	415.00	0.58
			Right	0.07	0.06	1.08	0.28	5.65E+00	415.00	0.60
		LAT	Left	0.01	0.28	0.03	0.98	1.95E+01	415.00	0.87
			Right	-0.18	0.25	-0.74	0.46	9.22E+00	415.00	0.90
		BAS	Left	-0.12	0.12	-1.03	0.30	6.03E+00	415.00	0.96
			Right	-0.07	0.12	-0.58	0.57	1.13E+01	415.00	0.95

Emotional Abuse	PTSD cases (n=187) and controls (n=246)	ACC	Left	-0.02	0.13	-0.16	0.87	1.74E+01	415.00	0.88
			Right	0.06	0.12	0.46	0.64	1.29E+01	415.00	0.90
		AAA	Left	0.01	0.05	0.23	0.82	1.63E+01	415.00	0.63
			Right	-0.06	0.05	-1.05	0.29	5.87E+00	415.00	0.65
		MED	Left	0.10	0.05	1.94	0.05	1.06E+00	415.00	0.36
			Right	0.08	0.05	1.61	0.11	2.17E+00	415.00	0.43
		COR	Left	0.01	0.03	0.37	0.71	1.43E+01	415.00	0.59
			Right	0.01	0.03	0.39	0.70	1.40E+01	415.00	0.67
		CAT	Left	-0.10	0.12	-0.89	0.37	7.49E+00	415.00	0.78
			Right	0.08	0.10	0.80	0.42	8.48E+00	415.00	0.81
		PAR	Left	-0.04	0.03	-1.57	0.12	2.35E+00	415.00	0.83
			Right	0.01	0.03	0.23	0.82	1.64E+01	415.00	0.78
		TOT	Left	1.82	1.51	1.21	0.23	4.53E+00	416.00	0.46
			Right	0.25	1.56	0.16	0.87	1.75E+01	416.00	0.46
Physical Abuse	PTSD cases (n=187) and controls (n=246)	CEN	Left	0.23	0.06	3.60	0.00	7.16E-03	415.00	0.58
			Right	0.16	0.07	2.29	0.02	4.48E-01	415.00	0.61
		LAT	Left	-0.17	0.30	-0.59	0.56	1.11E+01	415.00	0.87
			Right	0.26	0.27	0.96	0.34	6.70E+00	415.00	0.90
		BAS	Left	-0.12	0.13	-0.91	0.36	7.22E+00	415.00	0.96
			Right	-0.20	0.13	-1.54	0.12	2.49E+00	415.00	0.95
		ACC	Left	0.11	0.14	0.80	0.43	8.50E+00	415.00	0.88
			Right	-0.06	0.13	-0.47	0.64	1.27E+01	415.00	0.90
		AAA	Left	0.03	0.06	0.63	0.53	1.06E+01	415.00	0.63
			Right	-0.03	0.06	-0.53	0.60	1.20E+01	415.00	0.65
		MED	Left	0.03	0.06	0.61	0.54	1.09E+01	415.00	0.35
			Right	0.01	0.06	0.20	0.84	1.68E+01	415.00	0.43
		COR	Left	0.00	0.03	-0.06	0.95	1.91E+01	415.00	0.59
			Right	-0.03	0.03	-1.01	0.31	6.23E+00	415.00	0.67
		CAT	Left	-0.06	0.12	-0.50	0.62	1.23E+01	415.00	0.78
			Right	-0.09	0.11	-0.82	0.41	8.24E+00	415.00	0.81
		PAR	Left	-0.06	0.03	-2.00	0.05	9.24E-01	415.00	0.83
			Right	-0.01	0.03	-0.42	0.67	1.35E+01	415.00	0.78
		TOT	Left	1.56	1.61	0.97	0.33	6.69E+00	416.00	0.46
			Right	-0.56	1.68	-0.33	0.74	1.48E+01	416.00	0.46
Sexual Abuse	PTSD cases (n=187) and controls (n=246)	CEN	Left	0.01	0.05	0.18	0.85	1.71E+01	415.00	0.57
			Right	-0.01	0.06	-0.17	0.86	1.73E+01	415.00	0.60
		LAT	Left	-0.09	0.25	-0.36	0.72	1.45E+01	415.00	0.87
			Right	-0.09	0.22	-0.41	0.68	1.36E+01	415.00	0.90
		BAS	Left	0.10	0.11	0.91	0.36	7.23E+00	415.00	0.96
			Right	0.04	0.11	0.35	0.72	1.45E+01	415.00	0.95
		ACC	Left	0.01	0.12	0.07	0.94	1.88E+01	415.00	0.88
			Right	0.00	0.11	-0.01	0.99	1.98E+01	415.00	0.90
		AAA	Left	0.12	0.05	2.53	0.01	2.33E-01	415.00	0.64
			Right	0.02	0.05	0.48	0.63	1.26E+01	415.00	0.65
		MED	Left	-0.02	0.05	-0.46	0.64	1.29E+01	415.00	0.35
			Right	-0.01	0.05	-0.13	0.89	1.79E+01	415.00	0.43
		COR	Left	-0.03	0.03	-1.09	0.28	5.56E+00	415.00	0.60
			Right	0.00	0.02	-0.14	0.89	1.78E+01	415.00	0.67

		CAT	Left	-0.08	0.10	-0.75	0.45	9.10E+00	415.00	0.78
			Right	0.04	0.09	0.47	0.64	1.28E+01	415.00	0.81
		PAR	Left	-0.01	0.02	-0.51	0.61	1.23E+01	415.00	0.83
			Right	0.01	0.03	0.27	0.79	1.57E+01	415.00	0.78
		TOT	Left	0.47	1.36	0.34	0.73	1.46E+01	416.00	0.46
			Right	-0.36	1.41	-0.26	0.80	1.59E+01	416.00	0.46
Emotional Neglect	PTSD cases (n=187) and controls (n=246)	CEN	Left	0.00	0.06	0.03	0.97	1.95E+01	415.00	0.57
			Right	0.05	0.06	0.89	0.37	7.48E+00	415.00	0.60
		LAT	Left	0.45	0.26	1.71	0.09	1.77E+00	415.00	0.87
			Right	0.15	0.24	0.63	0.53	1.06E+01	415.00	0.90
		BAS	Left	-0.03	0.11	-0.25	0.80	1.60E+01	415.00	0.96
			Right	0.05	0.12	0.40	0.69	1.38E+01	415.00	0.95
		ACC	Left	-0.10	0.13	-0.79	0.43	8.62E+00	415.00	0.88
			Right	-0.02	0.11	-0.14	0.89	1.78E+01	415.00	0.90
		AAA	Left	-0.03	0.05	-0.52	0.60	1.21E+01	415.00	0.63
			Right	-0.05	0.05	-1.01	0.31	6.28E+00	415.00	0.65
		MED	Left	-0.07	0.05	-1.32	0.19	3.73E+00	415.00	0.36
			Right	-0.03	0.05	-0.63	0.53	1.06E+01	415.00	0.43
		COR	Left	-0.05	0.03	-1.58	0.11	2.29E+00	415.00	0.60
			Right	-0.05	0.03	-1.82	0.07	1.40E+00	415.00	0.67
		CAT	Left	-0.18	0.11	-1.60	0.11	2.20E+00	415.00	0.79
			Right	-0.12	0.10	-1.16	0.25	4.96E+00	415.00	0.81
		PAR	Left	-0.01	0.03	-0.23	0.82	1.63E+01	415.00	0.83
			Right	0.01	0.03	0.35	0.72	1.45E+01	415.00	0.78
		TOT	Left	0.72	1.44	0.50	0.62	1.24E+01	416.00	0.46
			Right	0.66	1.50	0.44	0.66	1.31E+01	416.00	0.46
Physical Neglect	PTSD cases (n=187) and controls (n=246)	CEN	Left	0.24	0.09	2.66	0.01	1.64E-01	415.00	0.58
			Right	0.29	0.09	3.02	0.00	5.33E-02	415.00	0.61
		LAT	Left	-0.16	0.41	-0.39	0.70	1.39E+01	415.00	0.87
			Right	-0.63	0.37	-1.71	0.09	1.77E+00	415.00	0.90
		BAS	Left	0.12	0.18	0.69	0.49	9.81E+00	415.00	0.96
			Right	0.09	0.18	0.49	0.62	1.25E+01	415.00	0.95
		ACC	Left	0.07	0.20	0.35	0.72	1.45E+01	415.00	0.88
			Right	0.30	0.18	1.71	0.09	1.78E+00	415.00	0.90
		AAA	Left	0.02	0.08	0.30	0.77	1.53E+01	415.00	0.63
			Right	-0.07	0.08	-0.93	0.36	7.10E+00	415.00	0.65
		MED	Left	0.03	0.08	0.36	0.72	1.44E+01	415.00	0.35
			Right	0.05	0.08	0.64	0.52	1.04E+01	415.00	0.43
		COR	Left	-0.05	0.05	-1.13	0.26	5.17E+00	415.00	0.60
			Right	-0.02	0.04	-0.49	0.62	1.25E+01	415.00	0.67
		CAT	Left	-0.24	0.17	-1.36	0.17	3.47E+00	415.00	0.79
			Right	-0.01	0.16	-0.03	0.97	1.95E+01	415.00	0.81
		PAR	Left	-0.03	0.04	-0.73	0.47	9.32E+00	415.00	0.83
			Right	0.00	0.04	0.04	0.97	1.94E+01	415.00	0.78
		TOT	Left	0.64	2.26	0.28	0.78	1.55E+01	416.00	0.46
			Right	-0.99	2.35	-0.42	0.67	1.35E+01	416.00	0.46
Total	PTSD cases (n=187) and controls (n=246)	CEN	Left	0.03	0.02	1.89	0.06	1.19E+00	415.00	0.57
			Right	0.03	0.02	1.50	0.13	2.67E+00	415.00	0.61

Total	PTSD cases (n=187) and controls (n=246)	LAT	Left	0.02	0.08	0.26	0.80	1.59E+01	415.00	0.87
			Right	-0.01	0.07	-0.18	0.85	1.71E+01	415.00	0.90
		BAS	Left	0.00	0.04	-0.08	0.93	1.87E+01	415.00	0.96
			Right	0.00	0.04	-0.02	0.99	1.98E+01	415.00	0.95
		ACC	Left	0.00	0.04	-0.12	0.90	1.80E+01	415.00	0.88
			Right	0.00	0.04	0.07	0.95	1.89E+01	415.00	0.90
		AAA	Left	0.02	0.02	1.01	0.31	6.22E+00	415.00	0.63
			Right	-0.02	0.02	-1.01	0.31	6.30E+00	415.00	0.65
		MED	Left	0.00	0.02	0.15	0.88	1.76E+01	415.00	0.35
			Right	0.01	0.02	0.45	0.65	1.31E+01	415.00	0.43
		COR	Left	-0.01	0.01	-1.27	0.21	4.11E+00	415.00	0.60
			Right	-0.01	0.01	-1.17	0.24	4.87E+00	415.00	0.67
		CAT	Left	-0.04	0.03	-1.29	0.20	3.98E+00	415.00	0.79
			Right	0.00	0.03	-0.08	0.93	1.87E+01	415.00	0.81
		PAR	Left	-0.01	0.01	-1.13	0.26	5.21E+00	415.00	0.83
			Right	0.00	0.01	0.48	0.63	1.26E+01	415.00	0.78
		TOT	Left	0.36	0.45	0.80	0.43	8.53E+00	416.00	0.46
			Right	-0.09	0.47	-0.19	0.85	1.70E+01	416.00	0.46
Combined Abuse	PTSD cases (n=187) and controls (n=246)	CEN	Left	0.06	0.02	2.47	0.01	2.80E-01	415.00	0.58
			Right	0.03	0.03	1.22	0.22	4.46E+00	415.00	0.61
		LAT	Left	-0.04	0.11	-0.37	0.71	1.42E+01	415.00	0.87
			Right	-0.01	0.10	-0.12	0.90	1.81E+01	415.00	0.90
		BAS	Left	-0.02	0.05	-0.35	0.72	1.45E+01	415.00	0.96
			Right	-0.03	0.05	-0.65	0.51	1.03E+01	415.00	0.95
		ACC	Left	0.01	0.05	0.27	0.79	1.58E+01	415.00	0.88
			Right	0.00	0.05	0.00	1.00	1.99E+01	415.00	0.90
		AAA	Left	0.03	0.02	1.46	0.14	2.87E+00	415.00	0.64
			Right	-0.01	0.02	-0.41	0.68	1.37E+01	415.00	0.65
		MED	Left	0.02	0.02	0.80	0.42	8.45E+00	415.00	0.35
			Right	0.01	0.02	0.67	0.51	1.01E+01	415.00	0.43
		COR	Left	0.00	0.01	-0.36	0.72	1.44E+01	415.00	0.59
			Right	0.00	0.01	-0.29	0.77	1.55E+01	415.00	0.67
		CAT	Left	-0.04	0.05	-0.89	0.38	7.53E+00	415.00	0.78
			Right	0.01	0.04	0.23	0.82	1.64E+01	415.00	0.81
		PAR	Left	-0.02	0.01	-1.62	0.11	2.13E+00	415.00	0.83
			Right	0.00	0.01	0.06	0.96	1.91E+01	415.00	0.78
		TOT	Left	0.62	0.61	1.01	0.31	6.26E+00	416.00	0.46
			Right	-0.11	0.63	-0.18	0.86	1.72E+01	416.00	0.46
Combined Neglect	PTSD cases (n=187) and controls (n=246)	CEN	Left	0.05	0.04	1.18	0.24	4.79E+00	415.00	0.57
			Right	0.08	0.04	1.93	0.05	1.09E+00	415.00	0.61
		LAT	Left	0.18	0.18	1.00	0.32	6.39E+00	415.00	0.87
			Right	-0.05	0.16	-0.31	0.75	1.51E+01	415.00	0.90
		BAS	Left	0.01	0.08	0.13	0.90	1.79E+01	415.00	0.96
			Right	0.04	0.08	0.49	0.63	1.25E+01	415.00	0.95
		ACC	Left	-0.03	0.09	-0.39	0.70	1.40E+01	415.00	0.88
			Right	0.05	0.08	0.65	0.52	1.03E+01	415.00	0.90
		AAA	Left	-0.01	0.03	-0.23	0.82	1.64E+01	415.00	0.63
			Right	-0.04	0.03	-1.10	0.27	5.47E+00	415.00	0.65

Combined Neglect	PTSD cases (n=187) and controls (n=246)	MED	Left	-0.03	0.03	-0.75	0.45	9.09E+00	415.00	0.35
			Right	-0.01	0.03	-0.15	0.88	1.76E+01	415.00	0.43
		COR	Left	-0.03	0.02	-1.58	0.11	2.29E+00	415.00	0.60
			Right	-0.03	0.02	-1.46	0.15	2.91E+00	415.00	0.67
		CAT	Left	-0.13	0.08	-1.70	0.09	1.81E+00	415.00	0.79
			Right	-0.06	0.07	-0.81	0.42	8.40E+00	415.00	0.81
		PAR	Left	-0.01	0.02	-0.48	0.63	1.26E+01	415.00	0.83
			Right	0.00	0.02	0.26	0.80	1.59E+01	415.00	0.78
		TOT	Left	0.46	0.99	0.46	0.64	1.29E+01	416.00	0.46
			Right	0.12	1.03	0.12	0.90	1.81E+01	416.00	0.46
* red highlight = P<0.05										

Table T5: Output from regression models for each predictor (ComBat-Gam harmonized volumes ~ Predictor† + Sex + Age2 + Age + ICV)

	Participants	ROI	Hemisphere	Beta	SE	t	P-value	pBON	DF	adj R^2
PTSD Diagnosis	PTSD cases (n=771) and all controls (n=1081)	CEN	Left	0.35	0.40	0.88	3.81E-01	7.62E+00	1842.00	0.19
			Right	-0.49	0.42	-1.16	2.44E-01	4.89E+00	1842.00	0.22
		LAT	Left	-5.25	2.95	-1.78	7.55E-02	1.51E+00	1842.00	0.37
			Right	-10.22	3.04	-3.36	7.89E-04	1.58E-02	1842.00	0.37
		BAS	Left	-4.61	2.08	-2.21	2.70E-02	5.40E-01	1842.00	0.38
			Right	-8.20	2.14	-3.82	1.35E-04	2.70E-03	1842.00	0.38
		ACC	Left	-2.25	1.40	-1.61	1.07E-01	2.14E+00	1842.00	0.33
			Right	-3.61	1.42	-2.54	1.10E-02	2.20E-01	1842.00	0.36
		AAA	Left	-0.69	0.35	-1.95	5.15E-02	1.03E+00	1842.00	0.22
			Right	-1.49	0.38	-3.94	8.34E-05	1.67E-03	1842.00	0.23
		MED	Left	0.16	0.30	0.54	5.92E-01	1.18E+01	1842.00	0.10
			Right	-0.19	0.30	-0.62	5.37E-01	1.07E+01	1842.00	0.11
		COR	Left	-0.05	0.21	-0.23	8.21E-01	1.64E+01	1842.00	0.17
			Right	-0.19	0.19	-1.00	3.17E-01	6.35E+00	1842.00	0.23
		CAT	Left	-2.01	0.96	-2.08	3.74E-02	7.49E-01	1842.00	0.33
			Right	-2.39	0.95	-2.52	1.19E-02	2.38E-01	1842.00	0.34
		PAR	Left	-0.46	0.24	-1.86	6.28E-02	1.26E+00	1842.00	0.37
			Right	-0.83	0.24	-3.47	5.35E-04	1.07E-02	1842.00	0.36
		TOT	Left	-16.79	7.11	-2.36	1.83E-02	3.66E-01	1842.00	0.42
			Right	-25.19	7.32	-3.44	5.90E-03	1.18E-01	1842.00	0.42
	PTSD cases (n=771) and healthy controls (n=271)	CEN	Left	-0.37	0.61	-0.61	5.45E-01	1.09E+01	1062.00	0.20
			Right	-1.39	0.64	-2.18	2.97E-02	5.94E-01	1062.00	0.23
		LAT	Left	-17.31	4.50	-3.85	1.28E-04	2.55E-03	1062.00	0.40
			Right	-24.15	4.57	-5.28	1.56E-07	3.12E-06	1062.00	0.41
		BAS	Left	-11.08	3.14	-3.53	4.41E-04	8.82E-03	1062.00	0.42
			Right	-18.35	3.25	-5.64	2.18E-08	4.36E-07	1062.00	0.42
		ACC	Left	-5.95	2.11	-2.82	4.87E-03	9.75E-02	1062.00	0.35
			Right	-9.82	2.12	-4.62	4.26E-06	8.52E-05	1062.00	0.40
		AAA	Left	-1.65	0.54	-3.07	2.20E-03	4.40E-02	1062.00	0.24
			Right	-2.62	0.57	-4.60	4.77E-06	9.54E-05	1062.00	0.26
		MED	Left	-0.06	0.46	-0.14	8.89E-01	1.78E+01	1062.00	0.10
			Right	-0.48	0.46	-1.04	3.00E-01	5.99E+00	1062.00	0.12
		COR	Left	-0.49	0.32	-1.51	1.32E-01	2.63E+00	1062.00	0.17
			Right	-0.85	0.29	-2.92	3.56E-03	7.12E-02	1062.00	0.26
		CAT	Left	-3.08	1.47	-2.09	3.67E-02	7.34E-01	1062.00	0.34
			Right	-6.15	1.43	-4.30	1.86E-05	3.73E-04	1062.00	0.38
		PAR	Left	-1.23	0.37	-3.34	8.62E-04	1.72E-02	1062.00	0.42
			Right	-1.85	0.37	-5.00	6.62E-07	1.32E-05	1062.00	0.40
		TOT	Left	-16.18	11.08	-1.46	1.45E-01	2.89E+00	1062.00	0.43
			Right	-35.24	11.27	-3.13	1.82E-02	3.64E-01	1062.00	0.44
	PTSD cases (n=771) and trauma-exposed controls (n=780)	CEN	Left	0.60	0.42	1.43	1.54E-01	3.09E+00	1571.00	0.20
			Right	-0.24	0.44	-0.53	5.95E-01	1.19E+01	1571.00	0.22
		LAT	Left	-1.50	3.12	-0.48	6.32E-01	1.26E+01	1571.00	0.37
			Right	-5.92	3.24	-1.83	6.77E-02	1.35E+00	1571.00	0.36
		BAS	Left	-2.21	2.21	-1.00	3.17E-01	6.33E+00	1571.00	0.38
			Right	-5.17	2.28	-2.26	2.38E-02	4.76E-01	1571.00	0.37

	PTSD cases (n=771) and trauma-exposed controls (n=780)	ACC	Left	-0.96	1.48	-0.65	5.16E-01	1.03E+01	1571.00	0.34
			Right	-1.85	1.51	-1.23	2.21E-01	4.41E+00	1571.00	0.36
		AAA	Left	-0.33	0.37	-0.87	3.82E-01	7.64E+00	1571.00	0.22
			Right	-1.10	0.40	-2.74	6.27E-03	1.25E-01	1571.00	0.22
		MED	Left	0.31	0.32	0.97	3.33E-01	6.65E+00	1571.00	0.10
			Right	-0.12	0.32	-0.36	7.20E-01	1.44E+01	1571.00	0.11
		COR	Left	0.08	0.22	0.34	7.31E-01	1.46E+01	1571.00	0.17
			Right	-0.01	0.21	-0.06	9.51E-01	1.90E+01	1571.00	0.23
		CAT	Left	-1.53	1.02	-1.50	1.34E-01	2.69E+00	1571.00	0.32
			Right	-1.25	1.01	-1.24	2.16E-01	4.31E+00	1571.00	0.34
		PAR	Left	-0.18	0.26	-0.70	4.83E-01	9.65E+00	1571.00	0.37
			Right	-0.53	0.26	-2.07	3.85E-02	7.70E-01	1571.00	0.35
		TOT	Left	-16.11	7.50	-2.15	3.19E-02	6.38E-01	1571.00	0.42
			Right	-22.36	7.77	-2.88	4.07E-03	8.15E-02	1571.00	0.42
Trauma exposure	Healthy controls (n=271) and trauma-exposed controls (n=780)	CEN	Left	-1.04	0.64	-1.62	1.05E-01	2.09E+00	1041.00	0.18
			Right	-0.98	0.68	-1.45	1.49E-01	2.97E+00	1041.00	0.20
		LAT	Left	-16.61	4.66	-3.56	3.84E-04	7.67E-03	1041.00	0.34
			Right	-18.48	4.80	-3.85	1.25E-04	2.49E-03	1041.00	0.34
		BAS	Left	-10.65	3.33	-3.20	1.43E-03	2.86E-02	1041.00	0.36
			Right	-12.03	3.36	-3.58	3.66E-04	7.31E-03	1041.00	0.36
		ACC	Left	-5.27	2.25	-2.34	1.92E-02	3.85E-01	1041.00	0.31
			Right	-6.79	2.26	-3.00	2.73E-03	5.46E-02	1041.00	0.34
		AAA	Left	-1.52	0.55	-2.74	6.33E-03	1.27E-01	1041.00	0.22
			Right	-1.48	0.60	-2.48	1.33E-02	2.65E-01	1041.00	0.21
		MED	Left	-0.51	0.49	-1.04	2.99E-01	5.98E+00	1041.00	0.10
			Right	-0.28	0.49	-0.57	5.70E-01	1.14E+01	1041.00	0.11
		COR	Left	-0.45	0.34	-1.34	1.82E-01	3.64E+00	1041.00	0.16
			Right	-0.70	0.31	-2.26	2.41E-02	4.81E-01	1041.00	0.22
		CAT	Left	-1.91	1.54	-1.24	2.15E-01	4.31E+00	1041.00	0.31
			Right	-4.15	1.51	-2.75	6.08E-03	1.22E-01	1041.00	0.32
		PAR	Left	-1.21	0.39	-3.13	1.82E-03	3.63E-02	1041.00	0.35
			Right	-1.21	0.37	-3.26	1.14E-03	2.28E-02	1041.00	0.34
		TOT	Left	-2.10	11.16	-0.19	8.51E-01	1.70E+01	1041.00	0.41
			Right	-10.84	11.49	-0.94	3.46E-01	6.92E+00	1041.00	0.41
	PTSD cases (n=771) and trauma-exposed contols (n=780) vs healthy controls (n=271)	CEN	Left	-1.14	0.62	-1.84	6.54E-02	1.31E+00	1811.00	0.19
			Right	-1.11	0.65	-1.71	8.71E-02	1.74E+00	1811.00	0.22
		LAT	Left	-16.66	4.57	-3.64	2.78E-04	5.56E-03	1811.00	0.37
			Right	-18.44	4.71	-3.92	9.27E-05	1.85E-03	1811.00	0.37
		BAS	Left	-10.45	3.23	-3.23	1.24E-03	2.49E-02	1811.00	0.38
			Right	-13.10	3.31	-3.96	7.94E-05	1.59E-03	1811.00	0.38
		ACC	Left	-5.53	2.17	-2.55	1.07E-02	2.14E-01	1811.00	0.33
			Right	-7.68	2.19	-3.50	4.68E-04	9.36E-03	1811.00	0.36
		AAA	Left	-1.53	0.54	-2.80	5.11E-03	1.02E-01	1811.00	0.23
			Right	-1.63	0.58	-2.79	5.35E-03	1.07E-01	1811.00	0.23
		MED	Left	-0.59	0.47	-1.25	2.10E-01	4.21E+00	1811.00	0.10
			Right	-0.35	0.47	-0.73	4.63E-01	9.26E+00	1811.00	0.11
		COR	Left	-0.52	0.33	-1.58	1.14E-01	2.29E+00	1811.00	0.17
			Right	-0.80	0.30	-2.66	7.81E-03	1.56E-01	1811.00	0.24

		CAT	Left	-1.85	1.50	-1.24	2.16E-01	4.32E+00	1811.00	0.33
			Right	-4.76	1.47	-3.25	1.19E-03	2.39E-02	1811.00	0.34
		PAR	Left	-1.17	0.38	-3.09	2.04E-03	4.08E-02	1811.00	0.37
			Right	-1.29	0.37	-3.49	4.86E-04	9.71E-03	1811.00	0.36
		TOT	Left	-3.02	11.05	-0.27	7.85E-01	1.57E+01	1811.00	0.42
			Right	-12.91	11.35	-1.14	2.56E-01	5.11E+00	1811.00	0.42
PTSD age of onset	PTSD cases (n=150)	CEN	Left	0.05	0.06	0.73	4.67E-01	9.35E+00	141.00	0.18
			Right	0.04	0.07	0.66	5.08E-01	1.02E+01	141.00	0.24
		LAT	Left	0.43	0.48	0.90	3.68E-01	7.37E+00	141.00	0.36
			Right	0.13	0.49	0.28	7.82E-01	1.56E+01	141.00	0.41
		BAS	Left	0.39	0.32	1.23	2.21E-01	4.43E+00	141.00	0.40
			Right	0.04	0.32	0.12	9.02E-01	1.80E+01	141.00	0.44
		ACC	Left	0.10	0.22	0.47	6.42E-01	1.28E+01	141.00	0.34
			Right	0.06	0.22	0.28	7.79E-01	1.56E+01	141.00	0.42
		AAA	Left	0.05	0.05	0.96	3.39E-01	6.79E+00	141.00	0.28
			Right	-0.03	0.06	-0.41	6.80E-01	1.36E+01	141.00	0.26
		MED	Left	-0.04	0.05	-0.70	4.84E-01	9.69E+00	141.00	0.10
			Right	0.00	0.05	0.04	9.72E-01	1.94E+01	141.00	0.10
		COR	Left	-0.02	0.04	-0.55	5.80E-01	1.16E+01	141.00	0.16
			Right	-0.01	0.03	-0.28	7.83E-01	1.57E+01	141.00	0.26
		CAT	Left	0.14	0.15	0.94	3.47E-01	6.94E+00	141.00	0.34
			Right	0.00	0.15	-0.03	9.78E-01	1.96E+01	141.00	0.39
		PAR	Left	0.04	0.04	1.16	2.48E-01	4.95E+00	141.00	0.41
			Right	0.00	0.03	-0.12	9.01E-01	1.80E+01	141.00	0.38
		TOT	Left	1.24	1.15	1.08	2.81E-01	5.62E+00	141.00	0.38
			Right	-0.24	1.18	-0.21	8.36E-01	1.67E+01	141.00	0.38
PTSD severity scores (CAPS-IV)	PTSD cases (n=215) and all controls (n=261)	CEN	Left	-0.02	0.02	-0.82	4.11E-01	8.22E+00	465.00	0.15
			Right	-0.02	0.02	-0.81	4.19E-01	8.37E+00	465.00	0.24
		LAT	Left	-0.69	0.18	-3.91	1.06E-04	2.13E-03	465.00	0.35
			Right	-0.86	0.18	-4.86	1.59E-06	3.18E-05	465.00	0.39
		BAS	Left	-0.48	0.13	-3.80	1.64E-04	3.27E-03	465.00	0.37
			Right	-0.37	0.13	-2.99	2.93E-03	5.85E-02	465.00	0.38
		ACC	Left	-0.26	0.08	-3.03	2.59E-03	5.19E-02	465.00	0.30
			Right	-0.20	0.08	-2.43	1.56E-02	3.12E-01	465.00	0.34
		AAA	Left	-0.07	0.02	-3.50	5.02E-04	1.00E-02	465.00	0.21
			Right	-0.07	0.02	-2.98	3.03E-03	6.06E-02	465.00	0.19
		MED	Left	-0.01	0.02	-0.56	5.77E-01	1.15E+01	465.00	0.05
			Right	-0.01	0.02	-0.39	6.95E-01	1.39E+01	465.00	0.09
		COR	Left	-0.02	0.01	-1.84	6.65E-02	1.33E+00	465.00	0.12
			Right	-0.02	0.01	-1.71	8.82E-02	1.76E+00	465.00	0.18
		CAT	Left	-0.21	0.06	-3.57	3.92E-04	7.84E-03	465.00	0.31
			Right	-0.14	0.06	-2.50	1.29E-02	2.58E-01	465.00	0.30
		PAR	Left	-0.06	0.01	-4.40	1.33E-05	2.65E-04	465.00	0.38
			Right	-0.05	0.01	-3.29	1.09E-03	2.18E-02	465.00	0.37
		TOT	Left	-0.70	0.42	-1.66	9.74E-02	1.95E+00	465.00	0.41
			Right	-0.78	0.43	-1.83	6.76E-02	1.35E+00	465.00	0.42
	PTSD cases (n=215)	CEN	Left	0.01	0.03	0.30	7.64E-01	1.53E+01	205.00	0.15
			Right	0.00	0.03	0.10	9.20E-01	1.84E+01	205.00	0.23

	PTSD cases (n=215)	LAT	Left	-0.59	0.24	-2.51	1.28E-02	2.57E-01	205.00	0.36
			Right	-0.73	0.23	-3.14	1.91E-03	3.82E-02	205.00	0.45
		BAS	Left	-0.28	0.17	-1.67	9.59E-02	1.92E+00	205.00	0.39
			Right	-0.20	0.17	-1.18	2.41E-01	4.82E+00	205.00	0.41
		ACC	Left	-0.15	0.11	-1.41	1.59E-01	3.18E+00	205.00	0.37
			Right	-0.06	0.11	-0.52	6.06E-01	1.21E+01	205.00	0.38
		AAA	Left	-0.07	0.03	-2.31	2.20E-02	4.40E-01	205.00	0.20
			Right	-0.05	0.03	-1.68	9.42E-02	1.88E+00	205.00	0.21
		MED	Left	0.00	0.02	0.08	9.38E-01	1.88E+01	205.00	0.08
			Right	0.00	0.02	-0.07	9.42E-01	1.88E+01	205.00	0.13
		COR	Left	-0.01	0.02	-0.66	5.13E-01	1.03E+01	205.00	0.17
			Right	0.00	0.02	-0.27	7.85E-01	1.57E+01	205.00	0.24
		CAT	Left	-0.18	0.08	-2.42	1.62E-02	3.24E-01	205.00	0.35
			Right	-0.05	0.08	-0.60	5.52E-01	1.10E+01	205.00	0.32
		PAR	Left	-0.04	0.02	-2.21	2.80E-02	5.61E-01	205.00	0.39
			Right	-0.03	0.02	-1.41	1.61E-01	3.22E+00	205.00	0.41
		TOT	Left	-0.83	0.59	-1.42	1.57E-01	3.13E+00	205.00	0.41
			Right	-0.77	0.59	-1.29	1.99E-01	3.99E+00	205.00	0.43
PTSD subscale: Re-experiencing (CAPS-IV)	PTSD cases (n=124) and controls (n=118)	CEN	Left	0.09	0.10	0.95	3.41E-01	6.82E+00	233.00	0.18
			Right	0.10	0.09	1.12	2.65E-01	5.29E+00	233.00	0.28
		LAT	Left	-0.73	0.74	-0.99	3.25E-01	6.50E+00	233.00	0.35
			Right	-1.47	0.71	-2.07	3.94E-02	7.88E-01	233.00	0.43
		BAS	Left	-0.24	0.50	-0.49	6.23E-01	1.25E+01	233.00	0.42
			Right	-0.20	0.48	-0.42	6.72E-01	1.34E+01	233.00	0.47
		ACC	Left	0.22	0.33	0.68	4.98E-01	9.96E+00	233.00	0.36
			Right	0.18	0.32	0.56	5.78E-01	1.16E+01	233.00	0.44
		AAA	Left	0.00	0.09	-0.01	9.92E-01	1.98E+01	233.00	0.23
			Right	-0.02	0.09	-0.18	8.55E-01	1.71E+01	233.00	0.24
		MED	Left	0.03	0.08	0.43	6.65E-01	1.33E+01	233.00	0.07
			Right	0.10	0.07	1.34	1.83E-01	3.65E+00	233.00	0.15
		COR	Left	0.04	0.05	0.80	4.24E-01	8.48E+00	233.00	0.14
			Right	0.05	0.05	1.11	2.69E-01	5.39E+00	233.00	0.25
		CAT	Left	-0.01	0.24	-0.04	9.67E-01	1.93E+01	233.00	0.35
			Right	0.02	0.23	0.10	9.24E-01	1.85E+01	233.00	0.39
		PAR	Left	-0.12	0.06	-2.07	3.94E-02	7.89E-01	233.00	0.41
			Right	-0.07	0.06	-1.18	2.41E-01	4.82E+00	233.00	0.44
		TOT	Left	-0.92	1.85	-0.50	6.19E-01	1.24E+01	233.00	0.40
			Right	-1.84	1.87	-0.98	3.26E-01	6.52E+00	233.00	0.42
	PTSD cases (n=124)	CEN	Left	0.07	0.12	0.56	5.78E-01	1.16E+01	116.00	0.17
			Right	0.11	0.11	1.01	3.16E-01	6.32E+00	116.00	0.33
		LAT	Left	-0.64	0.85	-0.76	4.50E-01	9.00E+00	116.00	0.40
			Right	-1.25	0.77	-1.62	1.08E-01	2.16E+00	116.00	0.54
		BAS	Left	-0.27	0.58	-0.46	6.45E-01	1.29E+01	116.00	0.46
			Right	-0.15	0.56	-0.26	7.94E-01	1.59E+01	116.00	0.51
		ACC	Left	0.11	0.37	0.29	7.70E-01	1.54E+01	116.00	0.43
			Right	0.28	0.37	0.77	4.45E-01	8.91E+00	116.00	0.49
		AAA	Left	0.01	0.11	0.11	9.11E-01	1.82E+01	116.00	0.20
			Right	0.00	0.11	-0.04	9.70E-01	1.94E+01	116.00	0.27

	PTSD cases (n=124)	MED	Left	0.02	0.09	0.20	8.40E-01	1.68E+01	116.00	0.08
			Right	0.13	0.09	1.56	1.20E-01	2.41E+00	116.00	0.17
		COR	Left	0.05	0.06	0.77	4.41E-01	8.82E+00	116.00	0.18
			Right	0.09	0.05	1.60	1.12E-01	2.24E+00	116.00	0.33
		CAT	Left	-0.10	0.26	-0.38	7.02E-01	1.40E+01	116.00	0.40
			Right	0.11	0.27	0.40	6.93E-01	1.39E+01	116.00	0.42
		PAR	Left	-0.12	0.07	-1.80	7.48E-02	1.50E+00	116.00	0.47
			Right	-0.05	0.06	-0.83	4.10E-01	8.20E+00	116.00	0.49
		TOT	Left	-2.68	2.18	-1.23	2.21E-01	4.42E+00	116.00	0.43
			Right	-2.80	2.20	-1.27	2.06E-01	4.12E+00	116.00	0.45
PTSD subscale: Avoidance (CAPS-IV)	PTSD cases (n=124) and controls (n=118)	CEN	Left	0.06	0.07	0.87	3.87E-01	7.74E+00	233.00	0.18
			Right	0.09	0.06	1.43	1.54E-01	3.08E+00	233.00	0.28
		LAT	Left	0.31	0.51	0.60	5.51E-01	1.10E+01	233.00	0.35
			Right	-0.23	0.50	-0.47	6.37E-01	1.27E+01	233.00	0.42
		BAS	Left	-0.03	0.35	-0.08	9.38E-01	1.88E+01	233.00	0.42
			Right	0.13	0.33	0.39	7.00E-01	1.40E+01	233.00	0.47
		ACC	Left	0.07	0.23	0.29	7.73E-01	1.55E+01	233.00	0.36
			Right	0.11	0.22	0.52	6.06E-01	1.21E+01	233.00	0.44
		AAA	Left	-0.06	0.06	-0.94	3.47E-01	6.94E+00	233.00	0.24
			Right	-0.07	0.06	-1.15	2.50E-01	4.99E+00	233.00	0.24
		MED	Left	0.08	0.06	1.43	1.53E-01	3.07E+00	233.00	0.08
			Right	0.03	0.05	0.52	6.00E-01	1.20E+01	233.00	0.14
		COR	Left	0.04	0.04	1.03	3.05E-01	6.09E+00	233.00	0.14
			Right	0.00	0.03	-0.10	9.18E-01	1.84E+01	233.00	0.25
		CAT	Left	-0.29	0.16	-1.80	7.29E-02	1.46E+00	233.00	0.36
			Right	-0.20	0.16	-1.23	2.21E-01	4.43E+00	233.00	0.39
		PAR	Left	-0.04	0.04	-1.07	2.85E-01	5.70E+00	233.00	0.40
			Right	-0.03	0.04	-0.72	4.71E-01	9.43E+00	233.00	0.43
		TOT	Left	0.81	1.28	0.63	5.28E-01	1.06E+01	233.00	0.40
			Right	0.51	1.30	0.39	6.96E-01	1.39E+01	233.00	0.42
	PTSD cases (n=124)	CEN	Left	0.06	0.08	0.71	4.77E-01	9.55E+00	116.00	0.17
			Right	0.12	0.07	1.58	1.16E-01	2.33E+00	116.00	0.34
		LAT	Left	0.50	0.60	0.83	4.06E-01	8.12E+00	116.00	0.40
			Right	-0.06	0.55	-0.11	9.09E-01	1.82E+01	116.00	0.53
		BAS	Left	0.28	0.41	0.69	4.89E-01	9.77E+00	116.00	0.46
			Right	0.35	0.39	0.90	3.70E-01	7.41E+00	116.00	0.51
		ACC	Left	0.10	0.26	0.40	6.89E-01	1.38E+01	116.00	0.43
			Right	0.32	0.26	1.25	2.15E-01	4.30E+00	116.00	0.49
		AAA	Left	-0.06	0.08	-0.71	4.79E-01	9.57E+00	116.00	0.20
			Right	-0.08	0.08	-1.01	3.13E-01	6.26E+00	116.00	0.28
		MED	Left	0.04	0.06	0.69	4.90E-01	9.80E+00	116.00	0.08
			Right	0.00	0.06	-0.01	9.90E-01	1.98E+01	116.00	0.16
		COR	Left	0.02	0.04	0.51	6.14E-01	1.23E+01	116.00	0.18
			Right	-0.01	0.04	-0.21	8.37E-01	1.67E+01	116.00	0.31
		CAT	Left	-0.31	0.18	-1.69	9.38E-02	1.88E+00	116.00	0.41
			Right	-0.07	0.19	-0.35	7.23E-01	1.45E+01	116.00	0.42
		PAR	Left	-0.01	0.05	-0.30	7.61E-01	1.52E+01	116.00	0.46
			Right	-0.01	0.05	-0.18	8.60E-01	1.72E+01	116.00	0.49

		TOT	Left	0.88	1.54	0.57	5.68E-01	1.14E+01	116.00	0.42
			Right	0.79	1.56	0.51	6.13E-01	1.23E+01	116.00	0.44
PTSD subscale: Arousal (CAPS-IV)	PTSD cases (n=124) and controls (n=118)	CEN	Left	0.10	0.09	1.19	2.35E-01	4.70E+00	233.00	0.18
			Right	0.09	0.08	1.11	2.69E-01	5.38E+00	233.00	0.28
		LAT	Left	-0.78	0.65	-1.20	2.31E-01	4.62E+00	233.00	0.35
			Right	-1.40	0.62	-2.25	2.55E-02	5.09E-01	233.00	0.44
		BAS	Left	-0.20	0.44	-0.47	6.41E-01	1.28E+01	233.00	0.42
			Right	-0.28	0.42	-0.67	5.01E-01	1.00E+01	233.00	0.47
		ACC	Left	0.18	0.29	0.64	5.26E-01	1.05E+01	233.00	0.36
			Right	0.09	0.28	0.32	7.48E-01	1.50E+01	233.00	0.44
		AAA	Left	0.02	0.08	0.25	8.01E-01	1.60E+01	233.00	0.23
			Right	0.01	0.08	0.10	9.20E-01	1.84E+01	233.00	0.24
		MED	Left	0.11	0.07	1.53	1.27E-01	2.54E+00	233.00	0.08
			Right	0.10	0.07	1.52	1.30E-01	2.60E+00	233.00	0.15
		COR	Left	0.05	0.05	1.08	2.82E-01	5.65E+00	233.00	0.14
			Right	0.00	0.04	0.02	9.86E-01	1.97E+01	233.00	0.25
		CAT	Left	0.11	0.21	0.51	6.09E-01	1.22E+01	233.00	0.36
			Right	0.04	0.20	0.18	8.61E-01	1.72E+01	233.00	0.39
		PAR	Left	-0.03	0.05	-0.56	5.77E-01	1.15E+01	233.00	0.40
			Right	-0.06	0.05	-1.15	2.52E-01	5.03E+00	233.00	0.44
		TOT	Left	0.90	1.62	0.55	5.81E-01	1.16E+01	233.00	0.40
			Right	-0.42	1.64	-0.25	8.00E-01	1.60E+01	233.00	0.42
	PTSD cases (n=124)	CEN	Left	0.00	0.15	-0.02	9.84E-01	1.97E+01	116.00	0.16
			Right	0.00	0.13	0.02	9.87E-01	1.97E+01	116.00	0.32
		LAT	Left	-1.42	1.04	-1.37	1.73E-01	3.47E+00	116.00	0.40
			Right	-1.39	0.95	-1.46	1.47E-01	2.94E+00	116.00	0.54
		BAS	Left	-0.42	0.71	-0.59	5.59E-01	1.12E+01	116.00	0.46
			Right	0.08	0.68	0.12	9.06E-01	1.81E+01	116.00	0.51
		ACC	Left	-0.36	0.46	-0.80	4.26E-01	8.52E+00	116.00	0.43
			Right	0.13	0.45	0.29	7.76E-01	1.55E+01	116.00	0.49
		AAA	Left	0.01	0.14	0.04	9.66E-01	1.93E+01	116.00	0.20
			Right	0.12	0.14	0.84	4.03E-01	8.06E+00	116.00	0.28
		MED	Left	0.09	0.11	0.82	4.14E-01	8.29E+00	116.00	0.08
			Right	0.03	0.11	0.27	7.91E-01	1.58E+01	116.00	0.16
		COR	Left	-0.02	0.07	-0.26	7.96E-01	1.59E+01	116.00	0.17
			Right	-0.04	0.07	-0.56	5.74E-01	1.15E+01	116.00	0.31
		CAT	Left	-0.18	0.33	-0.55	5.80E-01	1.16E+01	116.00	0.40
			Right	0.15	0.33	0.44	6.57E-01	1.31E+01	116.00	0.42
		PAR	Left	-0.02	0.09	-0.25	8.03E-01	1.61E+01	116.00	0.46
			Right	0.00	0.08	-0.04	9.66E-01	1.93E+01	116.00	0.49
		TOT	Left	-2.93	2.68	-1.09	2.76E-01	5.52E+00	116.00	0.43
			Right	-1.71	2.72	-0.63	5.30E-01	1.06E+01	116.00	0.44
Emotional Abuse	PTSD cases (n=187) and controls (n=246)	CEN	Left	0.14	0.08	1.81	0.07	1.41E+00	422.00	0.16
			Right	0.00	0.09	0.00	1.00	2.00E+01	422.00	0.20
		LAT	Left	-0.11	0.63	-0.17	0.87	1.74E+01	422.00	0.28
			Right	-1.01	0.64	-1.58	0.12	2.30E+00	422.00	0.31
		BAS	Left	-0.22	0.46	-0.47	0.64	1.27E+01	422.00	0.28
			Right	-0.65	0.45	-1.43	0.15	3.06E+00	422.00	0.32

Emotional Abuse	PTSD cases (n=187) and controls (n=246)	ACC	Left	-0.09	0.30	-0.31	0.76	1.52E+01	422.00	0.24
			Right	-0.32	0.31	-1.03	0.30	6.09E+00	422.00	0.27
		AAA	Left	0.01	0.07	0.14	0.89	1.78E+01	422.00	0.17
			Right	-0.13	0.08	-1.70	0.09	1.79E+00	422.00	0.19
		MED	Left	0.09	0.06	1.54	0.13	2.50E+00	422.00	0.09
			Right	0.05	0.06	0.75	0.45	9.02E+00	422.00	0.07
		COR	Left	0.01	0.04	0.17	0.86	1.73E+01	422.00	0.12
			Right	-0.02	0.04	-0.58	0.57	1.13E+01	422.00	0.16
		CAT	Left	-0.14	0.20	-0.68	0.50	9.93E+00	422.00	0.24
			Right	-0.14	0.21	-0.70	0.49	9.75E+00	422.00	0.26
		PAR	Left	-0.06	0.05	-1.05	0.29	5.86E+00	422.00	0.28
			Right	-0.05	0.05	-0.98	0.33	6.59E+00	422.00	0.30
		TOT	Left	1.50	1.44	1.04	0.30	5.96E+00	422.00	0.37
			Right	-0.14	1.49	-0.09	0.93	1.85E+01	422.00	0.37
Physical Abuse	PTSD cases (n=187) and controls (n=246)	CEN	Left	0.27	0.09	3.19	1.54E-03	3.07E-02	422.00	0.17
			Right	0.13	0.09	1.44	0.15	3.03E+00	422.00	0.20
		LAT	Left	0.27	0.69	0.39	0.70	1.40E+01	422.00	0.28
			Right	0.02	0.70	0.02	0.98	1.96E+01	422.00	0.30
		BAS	Left	0.18	0.50	0.36	0.72	1.43E+01	422.00	0.28
			Right	-0.43	0.49	-0.88	0.38	7.60E+00	422.00	0.32
		ACC	Left	0.31	0.33	0.95	0.34	6.85E+00	422.00	0.24
			Right	-0.21	0.34	-0.63	0.53	1.06E+01	422.00	0.27
		AAA	Left	0.09	0.08	1.05	0.29	5.85E+00	422.00	0.17
			Right	-0.05	0.08	-0.59	0.56	1.11E+01	422.00	0.19
		MED	Left	0.05	0.07	0.79	0.43	8.57E+00	422.00	0.09
			Right	-0.01	0.07	-0.14	0.89	1.78E+01	422.00	0.07
		COR	Left	0.02	0.05	0.49	0.62	1.24E+01	422.00	0.12
			Right	-0.05	0.04	-1.10	0.27	5.41E+00	422.00	0.17
		CAT	Left	0.07	0.22	0.33	0.74	1.49E+01	422.00	0.24
			Right	-0.17	0.22	-0.78	0.44	8.71E+00	422.00	0.26
		PAR	Left	-0.03	0.06	-0.54	0.59	1.18E+01	422.00	0.28
			Right	-0.04	0.05	-0.66	0.51	1.02E+01	422.00	0.30
		TOT	Left	1.73	1.57	1.10	0.27	5.42E+00	422.00	0.37
			Right	-0.53	1.62	-0.32	0.75	1.49E+01	422.00	0.37
Sexual Abuse	PTSD cases (n=187) and controls (n=246)	CEN	Left	-0.01	0.07	-0.11	0.91	1.82E+01	422.00	0.15
			Right	-0.08	0.08	-1.06	0.29	5.81E+00	422.00	0.20
		LAT	Left	-0.22	0.57	-0.39	0.70	1.40E+01	422.00	0.28
			Right	-0.73	0.57	-1.28	0.20	4.01E+00	422.00	0.31
		BAS	Left	-0.07	0.41	-0.17	0.86	1.73E+01	422.00	0.28
			Right	-0.44	0.41	-1.09	0.27	5.49E+00	422.00	0.32
		ACC	Left	-0.09	0.27	-0.31	0.75	1.51E+01	422.00	0.24
			Right	-0.32	0.28	-1.17	0.24	4.87E+00	422.00	0.27
		AAA	Left	0.11	0.07	1.62	0.11	2.10E+00	422.00	0.17
			Right	-0.04	0.07	-0.58	0.56	1.12E+01	422.00	0.19
		MED	Left	-0.03	0.05	-0.53	0.60	1.20E+01	422.00	0.09
			Right	-0.04	0.06	-0.62	0.54	1.07E+01	422.00	0.07
		COR	Left	-0.03	0.04	-0.90	0.37	7.38E+00	422.00	0.13
			Right	-0.03	0.04	-0.85	0.40	7.96E+00	422.00	0.16

		CAT	Left	-0.12	0.18	-0.67	0.50	1.00E+01	422.00	0.24
			Right	-0.13	0.18	-0.73	0.47	9.35E+00	422.00	0.26
		PAR	Left	-0.03	0.05	-0.61	0.54	1.08E+01	422.00	0.28
			Right	-0.04	0.04	-0.85	0.40	7.91E+00	422.00	0.30
		TOT	Left	0.59	1.29	0.46	0.65	1.29E+01	422.00	0.37
			Right	-0.40	1.34	-0.30	0.76	1.52E+01	422.00	0.37
Emotional Neglect	PTSD cases (n=187) and controls (n=246)	CEN	Left	0.02	0.07	0.33	0.74	1.48E+01	422.00	0.15
			Right	0.06	0.08	0.72	0.47	9.45E+00	422.00	0.20
		LAT	Left	0.67	0.58	1.15	0.25	4.99E+00	422.00	0.28
			Right	0.16	0.59	0.28	0.78	1.56E+01	422.00	0.30
		BAS	Left	0.19	0.42	0.45	0.66	1.31E+01	422.00	0.28
			Right	0.02	0.42	0.04	0.96	1.93E+01	422.00	0.32
		ACC	Left	0.06	0.28	0.21	0.83	1.67E+01	422.00	0.24
			Right	-0.03	0.28	-0.10	0.92	1.84E+01	422.00	0.27
		AAA	Left	0.03	0.07	0.46	0.64	1.29E+01	422.00	0.17
			Right	-0.05	0.07	-0.68	0.50	9.94E+00	422.00	0.19
		MED	Left	-0.06	0.06	-1.02	0.31	6.18E+00	422.00	0.09
			Right	-0.02	0.06	-0.33	0.74	1.49E+01	422.00	0.07
		COR	Left	-0.03	0.04	-0.68	0.49	9.89E+00	422.00	0.13
			Right	-0.04	0.04	-1.00	0.32	6.34E+00	422.00	0.17
		CAT	Left	-0.06	0.19	-0.31	0.76	1.52E+01	422.00	0.24
			Right	-0.12	0.19	-0.63	0.53	1.06E+01	422.00	0.26
		PAR	Left	0.01	0.05	0.22	0.83	1.66E+01	422.00	0.27
			Right	0.01	0.05	0.19	0.85	1.69E+01	422.00	0.30
		TOT	Left	0.41	1.32	0.31	0.76	1.51E+01	422.00	0.37
			Right	-0.08	1.36	-0.06	0.95	1.90E+01	422.00	0.37
Physical Neglect	PTSD cases (n=187) and controls (n=246)	CEN	Left	0.14	0.11	1.19	0.23	4.66E+00	422.00	0.16
			Right	0.11	0.12	0.92	0.36	7.19E+00	422.00	0.20
		LAT	Left	-1.21	0.90	-1.33	0.18	3.66E+00	422.00	0.28
			Right	-2.58	0.91	-2.85	4.55E-03	9.10E-02	422.00	0.32
		BAS	Left	-0.75	0.66	-1.14	0.25	5.10E+00	422.00	0.28
			Right	-1.42	0.65	-2.20	2.87E-02	5.74E-01	422.00	0.32
		ACC	Left	-0.42	0.43	-0.97	0.33	6.67E+00	422.00	0.24
			Right	-0.65	0.44	-1.48	0.14	2.77E+00	422.00	0.27
		AAA	Left	-0.06	0.11	-0.60	0.55	1.10E+01	422.00	0.17
			Right	-0.28	0.11	-2.50	1.27E-02	2.55E-01	422.00	0.20
		MED	Left	-0.02	0.09	-0.22	0.83	1.66E+01	422.00	0.09
			Right	-0.02	0.09	-0.27	0.79	1.57E+01	422.00	0.07
		COR	Left	-0.09	0.06	-1.44	0.15	3.00E+00	422.00	0.13
			Right	-0.10	0.06	-1.72	0.09	1.73E+00	422.00	0.17
		CAT	Left	-0.52	0.29	-1.80	0.07	1.47E+00	422.00	0.25
			Right	-0.59	0.29	-2.03	4.33E-02	8.66E-01	422.00	0.27
		PAR	Left	-0.12	0.08	-1.58	0.11	2.28E+00	422.00	0.28
			Right	-0.14	0.07	-1.98	4.87E-02	9.75E-01	422.00	0.30
		TOT	Left	-0.30	2.06	-0.14	0.89	1.77E+01	422.00	0.37
			Right	-2.02	2.13	-0.95	0.34	6.85E+00	422.00	0.38
Total	PTSD cases (n=187) and controls (n=246)	CEN	Left	0.04	0.02	1.91	0.06	1.14E+00	422.00	0.16
			Right	-0.11	0.18	-0.60	0.55	1.09E+01	422.00	0.31

Total	PTSD cases (n=187) and controls (n=246)	LAT	Left	0.19	0.18	1.08	0.28	5.58E+00	422.00	0.28
			Right	-0.06	0.13	-0.51	0.61	1.23E+01	422.00	0.32
		BAS	Left	0.12	0.13	0.91	0.36	7.29E+00	422.00	0.28
			Right	-0.05	0.09	-0.54	0.59	1.18E+01	422.00	0.27
		ACC	Left	0.07	0.09	0.78	0.44	8.71E+00	422.00	0.24
			Right	-0.02	0.02	-0.98	0.33	6.60E+00	422.00	0.19
		AAA	Left	0.04	0.02	1.87	0.06	1.25E+00	422.00	0.18
			Right	0.01	0.02	0.60	0.55	1.10E+01	422.00	0.20
		MED	Left	0.01	0.02	0.30	0.76	1.52E+01	422.00	0.09
			Right	0.00	0.02	0.16	0.88	1.75E+01	422.00	0.07
		COR	Left	0.00	0.01	-0.26	0.80	1.59E+01	422.00	0.12
			Right	-0.01	0.01	-1.07	0.28	5.68E+00	422.00	0.17
		CAT	Left	0.01	0.06	0.19	0.85	1.70E+01	422.00	0.24
			Right	-0.03	0.06	-0.51	0.61	1.23E+01	422.00	0.26
		PAR	Left	0.00	0.02	0.22	0.82	1.65E+01	422.00	0.27
			Right	0.00	0.01	-0.11	0.91	1.82E+01	422.00	0.30
		TOT	Left	0.30	0.41	0.74	0.46	9.24E+00	422.00	0.37
			Right	-0.27	0.42	-0.64	0.52	1.05E+01	422.00	0.37
Combined Abuse	PTSD cases (n=187) and controls (n=246)	CEN	Left	0.06	0.03	1.86	0.06	1.20E+00	422.00	0.16
			Right	0.00	0.03	0.05	0.96	1.91E+01	422.00	0.20
		LAT	Left	-0.03	0.26	-0.10	0.92	1.84E+01	422.00	0.28
			Right	-0.31	0.26	-1.21	0.23	4.55E+00	422.00	0.31
		BAS	Left	-0.02	0.19	-0.13	0.89	1.78E+01	422.00	0.28
			Right	-0.26	0.18	-1.40	0.16	3.24E+00	422.00	0.32
		ACC	Left	0.01	0.12	0.09	0.93	1.86E+01	422.00	0.24
			Right	-0.15	0.13	-1.18	0.24	4.80E+00	422.00	0.27
		AAA	Left	0.04	0.03	1.18	0.24	4.80E+00	422.00	0.17
			Right	-0.04	0.03	-1.17	0.24	4.86E+00	422.00	0.19
		MED	Left	0.02	0.02	0.68	0.50	1.00E+01	422.00	0.09
			Right	0.00	0.03	-0.03	0.98	1.96E+01	422.00	0.07
		COR	Left	0.00	0.02	-0.15	0.88	1.76E+01	422.00	0.12
			Right	-0.02	0.02	-1.03	0.31	6.12E+00	422.00	0.17
		CAT	Left	-0.04	0.08	-0.46	0.65	1.30E+01	422.00	0.24
			Right	-0.07	0.08	-0.90	0.37	7.38E+00	422.00	0.26
		PAR	Left	-0.02	0.02	-0.90	0.37	7.40E+00	422.00	0.28
			Right	-0.02	0.02	-1.02	0.31	6.13E+00	422.00	0.30
		TOT	Left	0.61	0.58	1.04	0.30	6.00E+00	422.00	0.37
			Right	-0.18	0.60	-0.29	0.77	1.54E+01	422.00	0.37
Combined Neglect	PTSD cases (n=187) and controls (n=246)	CEN	Left	0.04	0.05	0.75	0.46	9.12E+00	422.00	0.16
			Right	0.05	0.05	0.89	0.38	7.50E+00	422.00	0.20
		LAT	Left	0.08	0.39	0.20	0.84	1.68E+01	422.00	0.28
			Right	-0.41	0.40	-1.04	0.30	5.98E+00	422.00	0.31
		BAS	Left	-0.06	0.29	-0.19	0.85	1.69E+01	422.00	0.28
			Right	-0.26	0.28	-0.92	0.36	7.16E+00	422.00	0.32
		ACC	Left	-0.05	0.19	-0.28	0.78	1.56E+01	422.00	0.24
			Right	-0.14	0.19	-0.71	0.48	9.54E+00	422.00	0.27
		AAA	Left	0.00	0.05	0.06	0.96	1.91E+01	422.00	0.17
			Right	-0.07	0.05	-1.55	0.12	2.45E+00	422.00	0.19

Combined Neglect	PTSD cases (n=187) and controls (n=246)	MED	Left	-0.03	0.04	-0.79	0.43	8.64E+00	422.00	0.09
			Right	-0.01	0.04	-0.34	0.73	1.47E+01	422.00	0.07
		COR	Left	-0.03	0.03	-1.09	0.28	5.51E+00	422.00	0.13
			Right	-0.04	0.02	-1.43	0.15	3.08E+00	422.00	0.17
		CAT	Left	-0.13	0.13	-0.99	0.32	6.49E+00	422.00	0.24
			Right	-0.17	0.13	-1.31	0.19	3.84E+00	422.00	0.26
		PAR	Left	-0.02	0.03	-0.54	0.59	1.18E+01	422.00	0.28
			Right	-0.02	0.03	-0.73	0.47	9.37E+00	422.00	0.30
		TOT	Left	0.13	0.90	0.15	0.88	1.76E+01	422.00	0.37
			Right	-0.42	0.93	-0.45	0.65	1.30E+01	422.00	0.37
* red highlight = P<0.05											

Table T6: Output from regression models for each predictor (ComBat-Gam harmonized volumes ~ Predictor† + Sex + Age2 + Age + ICV + Whole amygdala volume)											
	Participants	ROI	Hemisphere	Beta	SE	t	P-value	pBON	DF	adj R^2
PTSD Diagnosis	PTSD cases (n=771) and all controls (n=1081)	CEN	Left	0.81	0.32	2.51	1.22E-02	2.44E-01	1841.00	0.47
			Right	0.40	0.34	1.19	2.35E-01	4.70E+00	1841.00	0.50
		LAT	Left	-0.08	1.33	-0.06	9.51E-01	1.90E+01	1841.00	0.87
			Right	-0.62	1.32	-0.47	6.35E-01	1.27E+01	1841.00	0.88
		BAS	Left	-0.68	0.56	-1.21	2.26E-01	4.51E+00	1841.00	0.96
			Right	-0.98	0.59	-1.67	9.49E-02	1.90E+00	1841.00	0.95
		ACC	Left	0.19	0.64	0.29	7.70E-01	1.54E+01	1841.00	0.86
			Right	0.88	0.61	1.44	1.50E-01	2.99E+00	1841.00	0.88
		AAA	Left	-0.19	0.24	-0.77	4.40E-01	8.80E+00	1841.00	0.63
			Right	-0.51	0.25	-2.00	4.53E-02	9.05E-01	1841.00	0.65
		MED	Left	0.42	0.27	1.55	1.22E-01	2.44E+00	1841.00	0.27
			Right	0.28	0.27	1.04	3.00E-01	6.00E+00	1841.00	0.29
		COR	Left	0.20	0.17	1.20	2.29E-01	4.59E+00	1841.00	0.47
			Right	0.23	0.15	1.50	1.33E-01	2.67E+00	1841.00	0.53
		CAT	Left	-0.43	0.53	-0.81	4.16E-01	8.32E+00	1841.00	0.79
			Right	0.44	0.50	0.89	3.73E-01	7.46E+00	1841.00	0.82
		PAR	Left	-0.05	0.13	-0.38	7.02E-01	1.40E+01	1841.00	0.82
			Right	-0.13	0.13	-0.97	3.34E-01	6.67E+00	1841.00	0.81
		TOT	Left	-16.79	7.11	-2.36	1.83E-02	3.66E-01	1842.00	0.42
			Right	-25.19	7.32	-3.44	5.90E-03	1.18E-01	1842.00	0.42
	PTSD cases (n=771) and healthy controls (n=271)	CEN	Left	0.90	0.50	1.79	7.38E-02	1.48E+00	1061.00	0.47
			Right	0.72	0.52	1.40	1.62E-01	3.23E+00	1061.00	0.51
		LAT	Left	-2.88	2.04	-1.41	1.58E-01	3.16E+00	1061.00	0.88
			Right	-1.33	1.97	-0.68	4.99E-01	9.98E+00	1061.00	0.89
		BAS	Left	-0.21	0.84	-0.25	8.06E-01	1.61E+01	1061.00	0.96
			Right	-1.11	0.91	-1.22	2.23E-01	4.45E+00	1061.00	0.96
		ACC	Left	0.79	0.97	0.82	4.13E-01	8.25E+00	1061.00	0.87
			Right	0.78	0.92	0.84	3.99E-01	7.97E+00	1061.00	0.89
		AAA	Left	-0.25	0.37	-0.68	4.97E-01	9.93E+00	1061.00	0.64
			Right	-0.28	0.39	-0.73	4.66E-01	9.31E+00	1061.00	0.67
		MED	Left	0.63	0.42	1.51	1.32E-01	2.63E+00	1061.00	0.26
			Right	0.58	0.42	1.36	1.75E-01	3.49E+00	1061.00	0.27
		COR	Left	0.19	0.27	0.70	4.86E-01	9.73E+00	1061.00	0.45
			Right	0.12	0.24	0.52	6.02E-01	1.20E+01	1061.00	0.53
		CAT	Left	1.34	0.81	1.65	9.93E-02	1.99E+00	1061.00	0.80
			Right	0.59	0.75	0.79	4.31E-01	8.62E+00	1061.00	0.83
		PAR	Left	-0.10	0.19	-0.54	5.92E-01	1.18E+01	1061.00	0.84
			Right	-0.14	0.20	-0.68	4.98E-01	9.97E+00	1061.00	0.82
		TOT	Left	-16.18	11.08	-1.46	1.45E-01	2.89E+00	1062.00	0.43
			Right	-35.24	11.27	-3.13	1.82E-02	3.64E-01	1062.00	0.44
	PTSD cases (n=771) and trauma-exposed controls (n=780)	CEN	Left	0.78	0.34	2.27	2.32E-02	4.64E-01	1570.00	0.47
			Right	0.28	0.36	0.78	4.35E-01	8.70E+00	1570.00	0.50
		LAT	Left	0.50	1.40	0.36	7.22E-01	1.44E+01	1570.00	0.87
			Right	-0.29	1.40	-0.21	8.34E-01	1.67E+01	1570.00	0.88
		BAS	Left	-0.69	0.60	-1.16	2.47E-01	4.94E+00	1570.00	0.95
			Right	-0.93	0.62	-1.50	1.34E-01	2.67E+00	1570.00	0.95

	PTSD cases (n=771) and trauma-exposed controls (n=780)	ACC	Left	-0.02	0.67	-0.03	9.76E-01	1.95E+01	1570.00	0.86
			Right	0.78	0.65	1.20	2.29E-01	4.58E+00	1570.00	0.88
		AAA	Left	-0.13	0.26	-0.52	6.05E-01	1.21E+01	1570.00	0.63
			Right	-0.52	0.27	-1.95	5.14E-02	1.03E+00	1570.00	0.65
		MED	Left	0.41	0.29	1.42	1.56E-01	3.12E+00	1570.00	0.27
			Right	0.16	0.29	0.53	5.93E-01	1.19E+01	1570.00	0.28
		COR	Left	0.17	0.18	0.97	3.32E-01	6.65E+00	1570.00	0.47
			Right	0.24	0.16	1.45	1.48E-01	2.95E+00	1570.00	0.53
		CAT	Left	-0.92	0.56	-1.63	1.03E-01	2.06E+00	1570.00	0.79
			Right	0.41	0.53	0.77	4.42E-01	8.85E+00	1570.00	0.82
		PAR	Left	-0.03	0.14	-0.18	8.53E-01	1.71E+01	1570.00	0.82
			Right	-0.11	0.14	-0.83	4.06E-01	8.13E+00	1570.00	0.81
		TOT	Left	-16.11	7.50	-2.15	3.19E-02	6.38E-01	1571.00	0.42
			Right	-22.36	7.77	-2.88	4.07E-03	8.15E-02	1571.00	0.42
Trauma exposure	Healthy controls (n=271) and trauma-exposed controls (n=780)	CEN	Left	0.21	0.51	0.41	6.85E-01	1.37E+01	1040.00	0.48
			Right	0.55	0.54	1.02	3.07E-01	6.13E+00	1040.00	0.50
		LAT	Left	-3.10	2.11	-1.47	1.43E-01	2.85E+00	1040.00	0.87
			Right	-2.54	2.14	-1.19	2.35E-01	4.69E+00	1040.00	0.87
		BAS	Left	-0.25	0.92	-0.27	7.86E-01	1.57E+01	1040.00	0.95
			Right	-0.06	0.94	-0.06	9.48E-01	1.90E+01	1040.00	0.95
		ACC	Left	1.23	1.03	1.20	2.30E-01	4.61E+00	1040.00	0.86
			Right	0.76	0.98	0.77	4.42E-01	8.84E+00	1040.00	0.88
		AAA	Left	-0.24	0.39	-0.60	5.47E-01	1.09E+01	1040.00	0.62
			Right	0.12	0.41	0.29	7.70E-01	1.54E+01	1040.00	0.63
		MED	Left	0.22	0.44	0.49	6.25E-01	1.25E+01	1040.00	0.29
			Right	0.59	0.43	1.37	1.71E-01	3.42E+00	1040.00	0.31
		COR	Left	0.24	0.26	0.92	3.60E-01	7.20E+00	1040.00	0.50
			Right	0.03	0.24	0.11	9.09E-01	1.82E+01	1040.00	0.53
		CAT	Left	2.25	0.86	2.62	9.00E-03	1.80E-01	1040.00	0.79
			Right	0.60	0.80	0.74	4.58E-01	9.17E+00	1040.00	0.81
		PAR	Left	-0.16	0.21	-0.74	4.59E-01	9.19E+00	1040.00	0.81
			Right	-0.06	0.21	-0.30	7.63E-01	1.53E+01	1040.00	0.80
		TOT	Left	-2.10	11.16	-0.19	8.51E-01	1.70E+01	1041.00	0.41
			Right	-10.84	11.49	-0.94	3.46E-01	6.92E+00	1041.00	0.41
	PTSD cases (n=771) and trauma-exposed contols (n=780) vs healthy controls (n=271)	CEN	Left	0.09	0.50	0.17	8.66E-01	1.73E+01	1810.00	0.47
			Right	0.47	0.52	0.91	3.64E-01	7.27E+00	1810.00	0.50
		LAT	Left	-2.90	2.06	-1.41	1.60E-01	3.19E+00	1810.00	0.87
			Right	-1.33	2.04	-0.65	5.14E-01	1.03E+01	1810.00	0.88
		BAS	Left	0.03	0.88	0.03	9.77E-01	1.95E+01	1810.00	0.95
			Right	-0.27	0.91	-0.29	7.71E-01	1.54E+01	1810.00	0.95
		ACC	Left	0.96	0.99	0.97	3.30E-01	6.61E+00	1810.00	0.86
			Right	0.30	0.94	0.31	7.54E-01	1.51E+01	1810.00	0.88
		AAA	Left	-0.21	0.38	-0.54	5.88E-01	1.18E+01	1810.00	0.63
			Right	0.11	0.39	0.29	7.75E-01	1.55E+01	1810.00	0.65
		MED	Left	0.10	0.43	0.25	8.06E-01	1.61E+01	1810.00	0.28
			Right	0.50	0.42	1.17	2.41E-01	4.83E+00	1810.00	0.29
		COR	Left	0.15	0.26	0.57	5.70E-01	1.14E+01	1810.00	0.47
			Right	-0.05	0.24	-0.20	8.41E-01	1.68E+01	1810.00	0.53

		CAT	Left	2.35	0.83	2.84	4.50E-03	9.00E-02	1810.00	0.80
			Right	0.28	0.77	0.36	7.20E-01	1.44E+01	1810.00	0.82
		PAR	Left	-0.09	0.20	-0.45	6.53E-01	1.31E+01	1810.00	0.82
			Right	-0.04	0.20	-0.20	8.45E-01	1.69E+01	1810.00	0.81
		TOT	Left	-3.02	11.05	-0.27	7.85E-01	1.57E+01	1811.00	0.42
			Right	-12.91	11.35	-1.14	2.56E-01	5.11E+00	1811.00	0.42
PTSD age of onset	PTSD cases (n=150)	CEN	Left	0.01	0.05	0.16	8.71E-01	1.74E+01	140.00	0.52
			Right	0.04	0.05	0.69	4.89E-01	9.78E+00	140.00	0.54
		LAT	Left	0.03	0.21	0.13	8.97E-01	1.79E+01	140.00	0.88
			Right	0.05	0.22	0.22	8.25E-01	1.65E+01	140.00	0.87
		BAS	Left	0.11	0.10	1.10	2.72E-01	5.43E+00	140.00	0.95
			Right	-0.02	0.09	-0.22	8.29E-01	1.66E+01	140.00	0.95
		ACC	Left	-0.08	0.10	-0.79	4.33E-01	8.66E+00	140.00	0.86
			Right	0.02	0.10	0.23	8.16E-01	1.63E+01	140.00	0.88
		AAA	Left	0.02	0.04	0.42	6.77E-01	1.35E+01	140.00	0.63
			Right	-0.04	0.04	-0.84	4.00E-01	8.00E+00	140.00	0.68
		MED	Left	-0.06	0.05	-1.29	1.98E-01	3.97E+00	140.00	0.30
			Right	0.00	0.04	-0.04	9.70E-01	1.94E+01	140.00	0.21
		COR	Left	-0.04	0.03	-1.34	1.81E-01	3.62E+00	140.00	0.44
			Right	-0.01	0.02	-0.55	5.84E-01	1.17E+01	140.00	0.59
		CAT	Left	0.03	0.09	0.32	7.51E-01	1.50E+01	140.00	0.76
			Right	-0.03	0.08	-0.34	7.32E-01	1.46E+01	140.00	0.81
		PAR	Left	0.01	0.02	0.68	4.97E-01	9.94E+00	140.00	0.81
			Right	-0.01	0.02	-0.46	6.43E-01	1.29E+01	140.00	0.77
		TOT	Left	1.24	1.15	1.08	2.81E-01	5.62E+00	141.00	0.38
			Right	-0.24	1.18	-0.21	8.36E-01	1.67E+01	141.00	0.38
PTSD severity scores (CAPS-IV)	PTSD cases (n=215) and all controls (n=261)	CEN	Left	0.04	0.02	2.32	2.09E-02	4.18E-01	464.00	0.52
			Right	0.03	0.02	1.61	1.09E-01	2.17E+00	464.00	0.49
		LAT	Left	-0.07	0.08	-0.86	3.92E-01	7.84E+00	464.00	0.88
			Right	-0.26	0.08	-3.44	6.27E-04	1.25E-02	464.00	0.89
		BAS	Left	0.00	0.03	-0.02	9.82E-01	1.96E+01	464.00	0.96
			Right	0.07	0.03	2.15	3.21E-02	6.43E-01	464.00	0.95
		ACC	Left	0.04	0.04	1.08	2.80E-01	5.60E+00	464.00	0.86
			Right	0.08	0.04	2.23	2.60E-02	5.20E-01	464.00	0.88
		AAA	Left	-0.01	0.01	-0.88	3.78E-01	7.55E+00	464.00	0.64
			Right	-0.01	0.02	-0.35	7.26E-01	1.45E+01	464.00	0.63
		MED	Left	0.02	0.02	1.44	1.51E-01	3.03E+00	464.00	0.26
			Right	0.02	0.02	1.47	1.43E-01	2.85E+00	464.00	0.28
		COR	Left	0.01	0.01	0.77	4.42E-01	8.83E+00	464.00	0.46
			Right	0.01	0.01	0.94	3.48E-01	6.96E+00	464.00	0.53
		CAT	Left	-0.02	0.03	-0.51	6.11E-01	1.22E+01	464.00	0.79
			Right	0.04	0.03	1.39	1.66E-01	3.32E+00	464.00	0.82
		PAR	Left	-0.02	0.01	-1.99	4.72E-02	9.45E-01	464.00	0.83
			Right	0.00	0.01	-0.29	7.72E-01	1.54E+01	464.00	0.81
		TOT	Left	-0.70	0.42	-1.66	9.74E-02	1.95E+00	465.00	0.41
			Right	-0.78	0.43	-1.83	6.76E-02	1.35E+00	465.00	0.42
	PTSD cases (n=215)	CEN	Left	0.05	0.03	1.98	4.89E-02	9.78E-01	204.00	0.46
			Right	0.03	0.03	1.26	2.10E-01	4.19E+00	204.00	0.46

	PTSD cases (n=215)	LAT	Left	-0.13	0.10	-1.31	1.90E-01	3.81E+00	204.00	0.88
			Right	-0.35	0.10	-3.64	3.50E-04	7.00E-03	204.00	0.91
		BAS	Left	0.07	0.04	1.60	1.11E-01	2.23E+00	204.00	0.96
			Right	0.09	0.04	2.12	3.50E-02	7.00E-01	204.00	0.96
		ACC	Left	0.06	0.05	1.16	2.48E-01	4.96E+00	204.00	0.88
			Right	0.12	0.05	2.61	9.67E-03	1.93E-01	204.00	0.89
		AAA	Left	-0.02	0.02	-1.05	2.95E-01	5.91E+00	204.00	0.64
			Right	-0.01	0.02	-0.48	6.32E-01	1.26E+01	204.00	0.68
		MED	Left	0.02	0.02	0.99	3.24E-01	6.48E+00	204.00	0.22
			Right	0.02	0.02	0.78	4.34E-01	8.68E+00	204.00	0.29
		COR	Left	0.01	0.01	0.75	4.52E-01	9.04E+00	204.00	0.46
			Right	0.01	0.01	1.15	2.53E-01	5.05E+00	204.00	0.56
		CAT	Left	-0.05	0.04	-1.13	2.59E-01	5.18E+00	204.00	0.80
			Right	0.07	0.04	1.84	6.76E-02	1.35E+00	204.00	0.82
		PAR	Left	-0.01	0.01	-0.71	4.79E-01	9.57E+00	204.00	0.84
			Right	0.00	0.01	0.18	8.57E-01	1.71E+01	204.00	0.83
		TOT	Left	-0.83	0.59	-1.42	1.57E-01	3.13E+00	205.00	0.41
			Right	-0.77	0.59	-1.29	1.99E-01	3.99E+00	205.00	0.43
PTSD subscale: Re-experiencing (CAPS-IV)	PTSD cases (n=124) and controls (n=118)	CEN	Left	0.12	0.08	1.49	1.38E-01	2.75E+00	232.00	0.48
			Right	0.13	0.08	1.64	1.03E-01	2.06E+00	232.00	0.43
		LAT	Left	-0.47	0.33	-1.45	1.48E-01	2.96E+00	232.00	0.87
			Right	-1.01	0.33	-3.09	2.21E-03	4.42E-02	232.00	0.88
		BAS	Left	-0.06	0.14	-0.43	6.70E-01	1.34E+01	232.00	0.95
			Right	0.13	0.15	0.87	3.85E-01	7.70E+00	232.00	0.95
		ACC	Left	0.34	0.16	2.11	3.60E-02	7.20E-01	232.00	0.85
			Right	0.38	0.15	2.48	1.37E-02	2.74E-01	232.00	0.87
		AAA	Left	0.02	0.06	0.39	7.00E-01	1.40E+01	232.00	0.63
			Right	0.03	0.07	0.46	6.49E-01	1.30E+01	232.00	0.61
		MED	Left	0.05	0.07	0.68	4.99E-01	9.98E+00	232.00	0.26
			Right	0.12	0.07	1.78	7.63E-02	1.53E+00	232.00	0.28
		COR	Left	0.05	0.04	1.26	2.10E-01	4.20E+00	232.00	0.43
			Right	0.07	0.04	1.91	5.77E-02	1.15E+00	232.00	0.51
		CAT	Left	0.07	0.13	0.49	6.28E-01	1.26E+01	232.00	0.79
			Right	0.16	0.13	1.21	2.26E-01	4.53E+00	232.00	0.80
		PAR	Left	-0.10	0.03	-3.13	1.97E-03	3.94E-02	232.00	0.82
			Right	-0.03	0.03	-0.99	3.21E-01	6.42E+00	232.00	0.81
		TOT	Left	-0.92	1.85	-0.50	6.19E-01	1.24E+01	233.00	0.40
			Right	-1.84	1.87	-0.98	3.26E-01	6.52E+00	233.00	0.42
	PTSD cases (n=124)	CEN	Left	0.09	0.10	0.92	3.61E-01	7.22E+00	115.00	0.40
			Right	0.12	0.10	1.27	2.05E-01	4.11E+00	115.00	0.44
		LAT	Left	-0.33	0.39	-0.85	3.97E-01	7.94E+00	115.00	0.87
			Right	-1.01	0.39	-2.60	1.05E-02	2.10E-01	115.00	0.88
		BAS	Left	-0.04	0.17	-0.23	8.17E-01	1.63E+01	115.00	0.96
			Right	0.04	0.17	0.25	8.06E-01	1.61E+01	115.00	0.95
		ACC	Left	0.24	0.18	1.32	1.91E-01	3.82E+00	115.00	0.86
			Right	0.39	0.18	2.15	3.34E-02	6.68E-01	115.00	0.87
		AAA	Left	0.05	0.08	0.56	5.76E-01	1.15E+01	115.00	0.59
			Right	0.02	0.08	0.31	7.59E-01	1.52E+01	115.00	0.64

	PTSD cases (n=124)	MED	Left	0.03	0.09	0.32	7.47E-01	1.49E+01	115.00	0.14
			Right	0.15	0.08	1.81	7.27E-02	1.45E+00	115.00	0.28
		COR	Left	0.06	0.05	1.13	2.60E-01	5.20E+00	115.00	0.38
			Right	0.10	0.04	2.24	2.71E-02	5.42E-01	115.00	0.56
		CAT	Left	-0.01	0.16	-0.09	9.30E-01	1.86E+01	115.00	0.78
			Right	0.18	0.15	1.18	2.41E-01	4.81E+00	115.00	0.80
		PAR	Left	-0.10	0.04	-2.59	1.09E-02	2.19E-01	115.00	0.83
			Right	-0.03	0.04	-0.89	3.77E-01	7.54E+00	115.00	0.81
		TOT	Left	-2.68	2.18	-1.23	2.21E-01	4.42E+00	116.00	0.43
			Right	-2.80	2.20	-1.27	2.06E-01	4.12E+00	116.00	0.45
PTSD subscale: Avoidance (CAPS-IV)	PTSD cases (n=124) and controls (n=118)	CEN	Left	0.06	0.06	1.01	3.14E-01	6.27E+00	232.00	0.48
			Right	0.09	0.05	1.69	9.27E-02	1.85E+00	232.00	0.43
		LAT	Left	0.26	0.23	1.15	2.52E-01	5.04E+00	232.00	0.87
			Right	-0.17	0.23	-0.73	4.68E-01	9.36E+00	232.00	0.88
		BAS	Left	-0.06	0.10	-0.61	5.43E-01	1.09E+01	232.00	0.95
			Right	0.18	0.10	1.73	8.53E-02	1.71E+00	232.00	0.95
		ACC	Left	0.05	0.11	0.41	6.79E-01	1.36E+01	232.00	0.85
			Right	0.14	0.11	1.34	1.83E-01	3.66E+00	232.00	0.87
		AAA	Left	-0.06	0.04	-1.47	1.44E-01	2.89E+00	232.00	0.63
			Right	-0.07	0.05	-1.46	1.45E-01	2.90E+00	232.00	0.61
		MED	Left	0.08	0.05	1.55	1.23E-01	2.46E+00	232.00	0.26
			Right	0.03	0.05	0.64	5.24E-01	1.05E+01	232.00	0.27
		COR	Left	0.03	0.03	1.19	2.36E-01	4.72E+00	232.00	0.43
			Right	0.00	0.03	-0.02	9.87E-01	1.97E+01	232.00	0.51
		CAT	Left	-0.31	0.09	-3.36	9.02E-04	1.80E-02	232.00	0.80
			Right	-0.18	0.09	-1.93	5.54E-02	1.11E+00	232.00	0.80
		PAR	Left	-0.05	0.02	-2.05	4.11E-02	8.21E-01	232.00	0.81
			Right	-0.02	0.02	-1.02	3.09E-01	6.19E+00	232.00	0.81
		TOT	Left	0.81	1.28	0.63	5.28E-01	1.06E+01	233.00	0.40
			Right	0.51	1.30	0.39	6.96E-01	1.39E+01	233.00	0.42
	PTSD cases (n=124)	CEN	Left	0.04	0.07	0.57	5.71E-01	1.14E+01	115.00	0.40
			Right	0.10	0.07	1.55	1.24E-01	2.47E+00	115.00	0.45
		LAT	Left	0.27	0.27	0.98	3.29E-01	6.58E+00	115.00	0.87
			Right	-0.25	0.28	-0.89	3.74E-01	7.48E+00	115.00	0.88
		BAS	Left	0.11	0.12	0.99	3.26E-01	6.52E+00	115.00	0.96
			Right	0.20	0.12	1.68	9.49E-02	1.90E+00	115.00	0.95
		ACC	Left	0.01	0.13	0.06	9.55E-01	1.91E+01	115.00	0.86
			Right	0.23	0.13	1.79	7.62E-02	1.52E+00	115.00	0.87
		AAA	Left	-0.08	0.06	-1.42	1.57E-01	3.14E+00	115.00	0.60
			Right	-0.10	0.06	-1.87	6.37E-02	1.27E+00	115.00	0.65
		MED	Left	0.04	0.06	0.60	5.52E-01	1.10E+01	115.00	0.14
			Right	-0.01	0.06	-0.16	8.70E-01	1.74E+01	115.00	0.26
		COR	Left	0.01	0.04	0.33	7.40E-01	1.48E+01	115.00	0.38
			Right	-0.02	0.03	-0.54	5.92E-01	1.18E+01	115.00	0.54
		CAT	Left	-0.38	0.11	-3.56	5.46E-04	1.09E-02	115.00	0.81
			Right	-0.13	0.11	-1.17	2.44E-01	4.88E+00	115.00	0.80
		PAR	Left	-0.03	0.03	-1.16	2.48E-01	4.96E+00	115.00	0.83
			Right	-0.02	0.03	-0.80	4.25E-01	8.49E+00	115.00	0.81

		TOT	Left	0.88	1.54	0.57	5.68E-01	1.14E+01	116.00	0.42
			Right	0.79	1.56	0.51	6.13E-01	1.23E+01	116.00	0.44
PTSD subscale: Arousal (CAPS-IV)	PTSD cases (n=124) and controls (n=118)	CEN	Left	0.12	0.07	1.70	9.00E-02	1.80E+00	232.00	0.48
			Right	0.12	0.07	1.72	8.69E-02	1.74E+00	232.00	0.43
		LAT	Left	-0.62	0.28	-2.17	3.07E-02	6.14E-01	232.00	0.87
			Right	-0.90	0.29	-3.13	1.94E-03	3.88E-02	232.00	0.88
		BAS	Left	-0.09	0.13	-0.71	4.77E-01	9.54E+00	232.00	0.95
			Right	0.08	0.13	0.60	5.48E-01	1.10E+01	232.00	0.95
		ACC	Left	0.25	0.14	1.81	7.13E-02	1.43E+00	232.00	0.85
			Right	0.31	0.13	2.31	2.19E-02	4.38E-01	232.00	0.87
		AAA	Left	0.03	0.05	0.65	5.18E-01	1.04E+01	232.00	0.63
			Right	0.06	0.06	1.02	3.07E-01	6.13E+00	232.00	0.61
		MED	Left	0.12	0.06	1.85	6.55E-02	1.31E+00	232.00	0.27
			Right	0.12	0.06	2.06	4.05E-02	8.10E-01	232.00	0.29
		COR	Left	0.06	0.04	1.52	1.31E-01	2.62E+00	232.00	0.43
			Right	0.02	0.03	0.68	4.98E-01	9.96E+00	232.00	0.51
		CAT	Left	0.15	0.12	1.30	1.95E-01	3.91E+00	232.00	0.79
			Right	0.19	0.12	1.61	1.09E-01	2.19E+00	232.00	0.80
		PAR	Left	-0.02	0.03	-0.59	5.58E-01	1.12E+01	232.00	0.81
			Right	-0.02	0.03	-0.71	4.81E-01	9.62E+00	232.00	0.81
		TOT	Left	0.90	1.62	0.55	5.81E-01	1.16E+01	233.00	0.40
			Right	-0.42	1.64	-0.25	8.00E-01	1.60E+01	233.00	0.42
	PTSD cases (n=124)	CEN	Left	0.07	0.12	0.55	5.86E-01	1.17E+01	115.00	0.40
			Right	0.02	0.12	0.19	8.52E-01	1.70E+01	115.00	0.44
		LAT	Left	-0.58	0.48	-1.21	2.27E-01	4.55E+00	115.00	0.87
			Right	-1.08	0.48	-2.25	2.62E-02	5.24E-01	115.00	0.88
		BAS	Left	0.20	0.20	1.00	3.20E-01	6.40E+00	115.00	0.96
			Right	0.32	0.21	1.52	1.31E-01	2.61E+00	115.00	0.95
		ACC	Left	0.00	0.23	-0.02	9.84E-01	1.97E+01	115.00	0.86
			Right	0.27	0.23	1.20	2.34E-01	4.68E+00	115.00	0.87
		AAA	Left	0.09	0.10	0.95	3.42E-01	6.84E+00	115.00	0.59
			Right	0.15	0.10	1.59	1.16E-01	2.31E+00	115.00	0.65
		MED	Left	0.12	0.11	1.11	2.71E-01	5.42E+00	115.00	0.15
			Right	0.04	0.10	0.42	6.73E-01	1.35E+01	115.00	0.26
		COR	Left	0.01	0.07	0.22	8.23E-01	1.65E+01	115.00	0.38
			Right	-0.02	0.06	-0.43	6.70E-01	1.34E+01	115.00	0.54
		CAT	Left	0.06	0.20	0.29	7.70E-01	1.54E+01	115.00	0.78
			Right	0.24	0.19	1.28	2.02E-01	4.03E+00	115.00	0.81
		PAR	Left	0.04	0.05	0.87	3.84E-01	7.68E+00	115.00	0.83
			Right	0.02	0.05	0.40	6.87E-01	1.37E+01	115.00	0.81
		TOT	Left	-2.93	2.68	-1.09	2.76E-01	5.52E+00	116.00	0.43
			Right	-1.71	2.72	-0.63	5.30E-01	1.06E+01	116.00	0.44
Emotional Abuse	PTSD cases (n=187) and controls (n=246)	CEN	Left	0.16	0.06	2.48	1.34E-02	2.67E-01	421	0.48
			Right	0.07	0.07	1.04	2.97E-01	5.94E+00	421	0.50
		LAT	Left	0.03	0.27	0.09	9.25E-01	1.85E+01	421	0.87
			Right	-0.22	0.26	-0.85	3.94E-01	7.88E+00	421	0.89
		BAS	Left	-0.12	0.11	-1.02	3.10E-01	6.19E+00	421	0.96
			Right	-0.06	0.12	-0.50	6.21E-01	1.24E+01	421	0.95

Emotional Abuse	PTSD cases (n=187) and controls (n=246)	ACC	Left	-0.03	0.13	-0.24	8.12E-01	1.62E+01	421	0.86
			Right	0.06	0.12	0.52	6.03E-01	1.21E+01	421	0.88
		AAA	Left	0.02	0.05	0.45	6.54E-01	1.31E+01	421	0.61
			Right	-0.05	0.05	-1.04	2.98E-01	5.95E+00	421	0.64
		MED	Left	0.10	0.06	1.81	7.13E-02	1.43E+00	421	0.26
			Right	0.09	0.06	1.61	1.08E-01	2.15E+00	421	0.29
		COR	Left	0.01	0.03	0.42	6.75E-01	1.35E+01	421	0.49
			Right	0.01	0.03	0.46	6.46E-01	1.29E+01	421	0.55
		CAT	Left	-0.10	0.11	-0.90	3.70E-01	7.40E+00	421	0.78
			Right	0.10	0.10	0.94	3.50E-01	7.00E+00	421	0.81
		PAR	Left	-0.05	0.03	-1.71	8.86E-02	1.77E+00	421	0.82
			Right	0.01	0.03	0.25	7.99E-01	1.60E+01	421	0.78
		TOT	Left	1.50	1.44	1.04	2.98E-01	5.96E+00	422.00	0.37
			Right	-0.14	1.49	-0.09	9.26E-01	1.85E+01	422.00	0.37
Physical Abuse	PTSD cases (n=187) and controls (n=246)	CEN	Left	0.24	0.07	3.54	4.53E-04	9.06E-03	421	0.49
			Right	0.07	0.07	1.04	2.97E-01	5.94E+00	421	0.50
		LAT	Left	-0.14	0.29	-0.48	6.32E-01	1.26E+01	421	0.87
			Right	-0.22	0.26	-0.85	3.94E-01	7.88E+00	421	0.89
		BAS	Left	-0.14	0.12	-1.08	2.79E-01	5.57E+00	421	0.96
			Right	-0.06	0.12	-0.50	6.21E-01	1.24E+01	421	0.95
		ACC	Left	0.12	0.14	0.84	4.00E-01	8.01E+00	421	0.86
			Right	0.06	0.12	0.52	6.03E-01	1.21E+01	421	0.88
		AAA	Left	0.05	0.06	0.84	4.00E-01	8.01E+00	421	0.61
			Right	-0.05	0.05	-1.04	2.98E-01	5.95E+00	421	0.64
		MED	Left	0.03	0.06	0.57	5.72E-01	1.14E+01	421	0.25
			Right	0.09	0.06	1.61	1.08E-01	2.15E+00	421	0.29
		COR	Left	0.00	0.04	0.09	9.27E-01	1.85E+01	421	0.49
			Right	0.01	0.03	0.46	6.46E-01	1.29E+01	421	0.55
		CAT	Left	-0.05	0.12	-0.41	6.86E-01	1.37E+01	421	0.77
			Right	0.10	0.10	0.94	3.50E-01	7.00E+00	421	0.81
		PAR	Left	-0.06	0.03	-2.21	2.76E-02	5.51E-01	421	0.82
			Right	0.01	0.03	0.25	7.99E-01	1.60E+01	421	0.78
		TOT	Left	1.73	1.57	1.10	2.71E-01	5.42E+00	422.00	0.37
			Right	-0.53	1.62	-0.32	7.46E-01	1.49E+01	422.00	0.37
Sexual Abuse	PTSD cases (n=187) and controls (n=246)	CEN	Left	0.01	0.06	0.13	8.96E-01	1.79E+01	421	0.47
			Right	-0.02	0.06	-0.39	6.94E-01	1.39E+01	421	0.50
		LAT	Left	-0.04	0.24	-0.16	8.75E-01	1.75E+01	421	0.87
			Right	-0.10	0.23	-0.41	6.79E-01	1.36E+01	421	0.89
		BAS	Left	0.07	0.10	0.68	4.96E-01	9.92E+00	421	0.96
			Right	0.03	0.11	0.30	7.65E-01	1.53E+01	421	0.95
		ACC	Left	0.00	0.12	0.01	9.91E-01	1.98E+01	421	0.86
			Right	-0.01	0.11	-0.13	9.01E-01	1.80E+01	421	0.88
		AAA	Left	0.12	0.05	2.77	5.77E-03	1.15E-01	421	0.62
			Right	0.02	0.05	0.48	6.32E-01	1.26E+01	421	0.63
		MED	Left	-0.02	0.05	-0.42	6.78E-01	1.36E+01	421	0.25
			Right	0.00	0.05	-0.04	9.72E-01	1.94E+01	421	0.29
		COR	Left	-0.03	0.03	-0.88	3.80E-01	7.60E+00	421	0.49
			Right	0.00	0.03	-0.03	9.79E-01	1.96E+01	421	0.55

		CAT	Left	-0.07	0.10	-0.69	4.89E-01	9.79E+00	421	0.78
			Right	0.06	0.09	0.65	5.16E-01	1.03E+01	421	0.81
		PAR	Left	-0.01	0.02	-0.61	5.43E-01	1.09E+01	421	0.82
			Right	0.01	0.03	0.29	7.73E-01	1.55E+01	421	0.78
		TOT	Left	0.59	1.29	0.46	6.47E-01	1.29E+01	422.00	0.37
			Right	-0.40	1.34	-0.30	7.62E-01	1.52E+01	422.00	0.37
Emotional Neglect	PTSD cases (n=187) and controls (n=246)	CEN	Left	0.00	0.06	0.01	9.96E-01	1.99E+01	421	0.47
			Right	0.06	0.06	0.93	3.55E-01	7.11E+00	421	0.50
		LAT	Left	0.39	0.25	1.57	1.17E-01	2.34E+00	421	0.87
			Right	0.18	0.24	0.74	4.60E-01	9.20E+00	421	0.89
		BAS	Left	-0.03	0.10	-0.29	7.69E-01	1.54E+01	421	0.96
			Right	0.03	0.11	0.24	8.09E-01	1.62E+01	421	0.95
		ACC	Left	-0.08	0.12	-0.65	5.19E-01	1.04E+01	421	0.86
			Right	-0.02	0.11	-0.20	8.43E-01	1.69E+01	421	0.88
		AAA	Left	0.00	0.05	0.10	9.18E-01	1.84E+01	421	0.61
			Right	-0.05	0.05	-0.99	3.22E-01	6.45E+00	421	0.64
		MED	Left	-0.07	0.05	-1.38	1.68E-01	3.36E+00	421	0.26
			Right	-0.02	0.05	-0.36	7.18E-01	1.44E+01	421	0.29
		COR	Left	-0.04	0.03	-1.35	1.77E-01	3.55E+00	421	0.49
			Right	-0.04	0.03	-1.34	1.80E-01	3.60E+00	421	0.55
		CAT	Left	-0.14	0.10	-1.39	1.66E-01	3.33E+00	421	0.78
			Right	-0.11	0.09	-1.22	2.24E-01	4.49E+00	421	0.81
		PAR	Left	-0.01	0.02	-0.49	6.23E-01	1.25E+01	421	0.82
			Right	0.01	0.03	0.38	7.06E-01	1.41E+01	421	0.78
		TOT	Left	0.41	1.32	0.31	7.55E-01	1.51E+01	422.00	0.37
			Right	-0.08	1.36	-0.06	9.52E-01	1.90E+01	422.00	0.37
Physical Neglect	PTSD cases (n=187) and controls (n=246)	CEN	Left	0.23	0.09	2.54	1.13E-02	2.27E-01	421	0.48
			Right	0.29	0.10	3.04	2.48E-03	4.96E-02	421	0.51
		LAT	Left	-0.15	0.39	-0.38	7.04E-01	1.41E+01	421	0.87
			Right	-0.62	0.37	-1.66	9.69E-02	1.94E+00	421	0.89
		BAS	Left	0.07	0.16	0.46	6.49E-01	1.30E+01	421	0.96
			Right	0.06	0.17	0.34	7.31E-01	1.46E+01	421	0.95
		ACC	Left	0.09	0.18	0.48	6.30E-01	1.26E+01	421	0.86
			Right	0.31	0.18	1.74	8.29E-02	1.66E+00	421	0.89
		AAA	Left	0.04	0.07	0.52	6.07E-01	1.21E+01	421	0.61
			Right	-0.08	0.08	-1.10	2.72E-01	5.44E+00	421	0.64
		MED	Left	0.03	0.08	0.37	7.11E-01	1.42E+01	421	0.25
			Right	0.08	0.08	1.00	3.17E-01	6.35E+00	421	0.29
		COR	Left	-0.04	0.05	-0.79	4.29E-01	8.58E+00	421	0.49
			Right	-0.01	0.04	-0.15	8.81E-01	1.76E+01	421	0.55
		CAT	Left	-0.21	0.16	-1.30	1.95E-01	3.90E+00	421	0.78
			Right	0.01	0.15	0.05	9.60E-01	1.92E+01	421	0.81
		PAR	Left	-0.04	0.04	-0.92	3.58E-01	7.15E+00	421	0.82
			Right	0.00	0.04	-0.01	9.89E-01	1.98E+01	421	0.78
		TOT	Left	-0.30	2.06	-0.14	8.86E-01	1.77E+01	422.00	0.37
			Right	-2.02	2.13	-0.95	3.43E-01	6.85E+00	422.00	0.38
Total CTQ score	PTSD cases (n=187) and controls (n=246)	CEN	Left	0.03	0.02	1.64	1.01E-01	2.03E+00	421	0.48
			Right	0.02	0.02	1.19	2.33E-01	4.66E+00	421	0.50

Total CTQ score	PTSD cases (n=187) and controls (n=246)	LAT	Left	0.03	0.08	0.43	6.69E-01	1.34E+01	421	0.87
			Right	-0.02	0.07	-0.23	8.22E-01	1.64E+01	421	0.89
		BAS	Left	-0.01	0.03	-0.23	8.22E-01	1.64E+01	421	0.96
			Right	0.00	0.03	0.12	9.02E-01	1.80E+01	421	0.95
		ACC	Left	-0.01	0.04	-0.29	7.74E-01	1.55E+01	421	0.86
			Right	0.00	0.03	-0.07	9.43E-01	1.89E+01	421	0.88
		AAA	Left	0.02	0.01	1.67	9.67E-02	1.93E+00	421	0.62
			Right	-0.01	0.01	-0.83	4.05E-01	8.09E+00	421	0.64
		MED	Left	0.00	0.02	-0.14	8.93E-01	1.79E+01	421	0.25
			Right	0.01	0.02	0.49	6.26E-01	1.25E+01	421	0.29
		COR	Left	-0.01	0.01	-1.18	2.38E-01	4.76E+00	421	0.49
			Right	-0.01	0.01	-0.94	3.47E-01	6.93E+00	421	0.55
		CAT	Left	-0.04	0.03	-1.19	2.37E-01	4.74E+00	421	0.78
			Right	0.00	0.03	-0.04	9.66E-01	1.93E+01	421	0.81
		PAR	Left	-0.01	0.01	-1.27	2.03E-01	4.06E+00	421	0.82
			Right	0.00	0.01	0.63	5.31E-01	1.06E+01	421	0.78
		TOT	Left	0.30	0.41	0.74	4.62E-01	9.24E+00	422.00	0.37
			Right	-0.27	0.42	-0.64	5.25E-01	1.05E+01	422.00	0.37
Combined Abuse	PTSD cases (n=187) and controls (n=246)	CEN	Left	0.06	0.03	2.37	1.85E-02	3.70E-01	421	0.48
			Right	0.03	0.03	1.05	2.94E-01	5.88E+00	421	0.50
		LAT	Left	-0.02	0.11	-0.21	8.33E-01	1.67E+01	421	0.87
			Right	-0.01	0.10	-0.13	8.99E-01	1.80E+01	421	0.89
		BAS	Left	-0.02	0.05	-0.51	6.14E-01	1.23E+01	421	0.96
			Right	-0.03	0.05	-0.68	4.99E-01	9.98E+00	421	0.95
		ACC	Left	0.01	0.05	0.22	8.25E-01	1.65E+01	421	0.86
			Right	0.00	0.05	-0.04	9.68E-01	1.94E+01	421	0.88
		AAA	Left	0.04	0.02	1.74	8.22E-02	1.64E+00	421	0.62
			Right	-0.01	0.02	-0.34	7.31E-01	1.46E+01	421	0.63
		MED	Left	0.02	0.02	0.75	4.53E-01	9.07E+00	421	0.25
			Right	0.02	0.02	0.67	5.04E-01	1.01E+01	421	0.29
		COR	Left	0.00	0.01	-0.19	8.47E-01	1.69E+01	421	0.49
			Right	0.00	0.01	-0.23	8.20E-01	1.64E+01	421	0.55
		CAT	Left	-0.04	0.05	-0.83	4.10E-01	8.19E+00	421	0.78
			Right	0.02	0.04	0.39	7.00E-01	1.40E+01	421	0.81
		PAR	Left	-0.02	0.01	-1.78	7.49E-02	1.50E+00	421	0.82
			Right	0.00	0.01	0.05	9.64E-01	1.93E+01	421	0.78
		TOT	Left	0.61	0.58	1.04	3.00E-01	6.00E+00	422.00	0.37
			Right	-0.18	0.60	-0.29	7.68E-01	1.54E+01	422.00	0.37
Combined Neglect	PTSD cases (n=187) and controls (n=246)	CEN	Left	0.04	0.04	1.10	2.71E-01	5.43E+00	421	0.48
			Right	0.08	0.04	1.94	5.29E-02	1.06E+00	421	0.51
		LAT	Left	0.15	0.17	0.90	3.69E-01	7.37E+00	421	0.87
			Right	-0.03	0.16	-0.22	8.30E-01	1.66E+01	421	0.89
		BAS	Left	0.00	0.07	0.00	9.98E-01	2.00E+01	421	0.96
			Right	0.02	0.08	0.31	7.54E-01	1.51E+01	421	0.95
		ACC	Left	-0.02	0.08	-0.23	8.19E-01	1.64E+01	421	0.86
			Right	0.05	0.08	0.62	5.39E-01	1.08E+01	421	0.88
		AAA	Left	0.01	0.03	0.29	7.69E-01	1.54E+01	421	0.61
			Right	-0.04	0.03	-1.15	2.51E-01	5.01E+00	421	0.64

Combined Neglect	PTSD cases (n=187) and controls (n=246)	MED	Left	-0.03	0.03	-0.78	4.39E-01	8.77E+00	421	0.25
			Right	0.01	0.03	0.19	8.52E-01	1.70E+01	421	0.29
		COR	Left	-0.03	0.02	-1.26	2.08E-01	4.15E+00	421	0.49
			Right	-0.02	0.02	-0.98	3.28E-01	6.56E+00	421	0.55
		CAT	Left	-0.10	0.07	-1.51	1.33E-01	2.65E+00	421	0.78
			Right	-0.05	0.06	-0.81	4.21E-01	8.42E+00	421	0.81
		PAR	Left	-0.01	0.02	-0.73	4.63E-01	9.26E+00	421	0.82
			Right	0.00	0.02	0.25	8.02E-01	1.60E+01	421	0.78
		TOT	Left	0.13	0.90	0.15	8.81E-01	1.76E+01	422.00	0.37
			Right	-0.42	0.93	-0.45	6.50E-01	1.30E+01	422.00	0.37
* red highlight = P<0.05										
